# National and sub-national burden and trend of type 1 diabetes in 31 provinces of Iran, 1990–2019

**DOI:** 10.1038/s41598-023-31096-8

**Published:** 2023-03-14

**Authors:** Fatemeh Bandarian, Yeganeh Sharifnejad Tehrani, Maryam Peimani, Nazli Namazi, Sahar Saeedi Moghaddam, Shahnaz Esmaeili, Mohammad-Mahdi Rashidi, Ensieh Nasli Esfahani, Masoud Masinaei, Negar Rezaei, Nazila Rezaei, Farshad Farzadfar, Bagher Larijani

**Affiliations:** 1 Present Address: Metabolomics and Genomics Research Center, Endocrinology and Metabolism Molecular- Cellular Sciences Institute, Tehran University of Medical Sciences, Tehran, Iran; 2 Non-Communicable Diseases Research Center, Endocrinology and Metabolism Population Sciences Institute, Tehran University of Medical Sciences, Tehran, Iran; 3 Elderly Health Research Center, Endocrinology and Metabolism Population Sciences Institute, Tehran University of Medical Sciences, Tehran, Iran; 4 Metabolic Disorders Research Center, Endocrinology and Metabolism Molecular -Cellular Sciences Institute, Tehran University of Medical Sciences, Tehran, Iran; 5 Kiel Institute for the World Economy, Kiel, Germany; 6 Evidence Based Medicine Research Center, Endocrinology and Metabolism Clinical Sciences Institute, Tehran University of Medical Sciences, Tehran, Iran; 7 Diabetes Research Center, Endocrinology and Metabolism Clinical Sciences Institute, Tehran University of Medical Sciences, 5th Flat, Diabetes Clinic, Cross Heyat Ave., Shahrivar Ave., North Kargar St., Tehran, 1411715851 Iran; 8 Endocrinology and Metabolism Research Center, Endocrinology and Metabolism Clinical Sciences Institute, Tehran University of Medical Sciences, Tehran, Iran

**Keywords:** Medical research, Endocrinology, Endocrine system and metabolic diseases, Health care, Public health

## Abstract

The aim of the study was to report the burden of type one diabetes mellitus (T1DM) by sex, age, year, and province in Iran over the past 30 years, according to data provided by the global burden of disease (GBD) study. Incidence, prevalence, death, disability-adjusted life-years (DALYs), years of life lost, and years lived with disability due to T1DM by age groups and sex was reported for 31 provinces of Iran from 1990 to 2019 with their 95% uncertainty intervals (UI). In 2019, national age-standardized incidence (11.0 (95% UI: 8.9–13.5)), prevalence (388.9 (306.1–482.1)), death (0.7 (0.6–0.8)), and DALYs (51.7 (40.9–65.1)) rates per 100,000 wre higher than 1990 except for death. Also, the mortality to incidence ratio reduced in all provinces over time particularly after 2014 as well. GBD data analysis showed that age-standardized incidence and prevalence rates of T1DM have increased, the death rate reduced, and DALYs remained unchanged during the past 30 years in Iran and its 31 provinces. death rate reduced and DALYs remained unchanged during the past 30 years in Iran and its 31 provinces.

## Introduction

Currently, diabetes mellitus is the 5th leading cause of death in Iran, as previously predicted by World Health Organization (WHO)^1^, but it has reached this point sooner than expected. According to WHO projection, diabetes will be the 5th leading cause of death worldwide by 2030^1^.

Type 1 diabetes mellitus (T1DM), known as juvenile diabetes or insulin-dependent diabetes, is the second most common type of diabetes and makes up around 5–10% of diabetes cases^2^. Pathogenesis of T1DM is different from type 2 diabetes. T1DM is an organ-specific autoimmune disease that causes T cell-mediated destruction of insulin-producing β -cells of islets of Langerhans, which results in almost complete ablation of β-cell secretory function that leads to complete or partial insulin deficiency^3,4^.

Risk factors of T1DM are less known. However, positive family history of T1DM, genetics, race/ethnicity, and environmental factors such as geography, nutrition, and some viral infections and vaccinations have been linked to the development of T1DM^2,5^.

According to the latest report, the proportion of T1DM among all types of diabetes in Iran was approximately 11.4% in 2016^6^. The incidence rate of T1DM increased 3–4% annually in European countries from 1989 to 2013, indicating a doubling incidence rate over 20 years^7^.

Similarly, an increasing annual rate of 1.8% in the United States (2002–2012)^8^ and 2.8% worldwide (1990–1999) for T1DM has been reported as well^9^. The highest estimated number of T1DM patients (0–19 y) in 2019 reported by the International Diabetes Federation (IDF) was in Europe, with 296.5 in 1000 s^2^. South and East Asia and the Middle East and North Africa (MENA) regions were in the 3rd and 4th rank of T1DM numbers in the world, respectively^2^. The number of T1DM (0–19 y) in Iran reported by IDF in 2021 was 8.2 in 1000 s^10^.

As the onset of T1DM is usually in childhood and early adulthood (younger than type 2 diabetes) and patients need regular insulin injections for glycemic control for the long term which are expensive with an invasive application, it reduces the quality of life and imposes a significant burden to the society and health system^11^.

The objective of this study was to present new results for the burden of T1DM (including incidence, prevalence, death and DALYs) in 31 provinces of Iran over the period of 1990 to 2019.

## Materials and methods

The general methods of the global burden of disease (GBD), including estimation of incidence, prevalence, and death rates of non-communicable diseases (NCDs), such as T1DM, and their protocols and updates, were published previously elsewhere^12–16^. The detailed methodology of GBD 2019 for estimating the burden of disease and risk factors and their changes from GBD 2017 have been described elsewhere^15,16^. Briefly, to estimate the prevalence of T1DM, obesity prevalence per province was used as a covariate in the DisMod-MR, a Bayesian meta-regression tool. Years lived with disability (YLDs) were calculated as the product of prevalence by age, sex, year and province times the diabetes-specific disability weight. To estimate the death due to T1DM, healthcare access and quality index, education years per capita, age-standardised fertility rate, latitude, age-standardised underweight (weight-forage) summary exposure variable, percentage of births occurring in women > 35 years old, percentage of births occurring in women > 40 years old, Socio-demographic Index (SDI), age-standardised stunting (height-for-age) summary exposure variable, and mean birth weight were considered as the covariates. Years of life lost (YLLs) were calculated from age-sex-province-specific estimates of death due to T1DM by life expectancy at each age. Disability-adjusted life-years (DALYs) were calculated as the summation of YLDs and YLLs.

In GBD 2019, death due to T1DM was calculated using mapping of the 10th revision of the International Statistical Classification of Diseases and Related Health Problems (ICD-10) codes, including E10–E10.1, E10.3–E10.9, and P70.2^15^. In addition, E10–E10.11 and E10.3–E10.9 were used to map new cases of T1DM^17^. Decomposition analysis was applied to extract and identify the most effective factors in trends and changes in incidence rates due to T1DM in different provinces from 1990 to 2019^18^. Also, SDI was calculated for all 31 provinces. SDI is a measure that indicates the range of development of countries or other geographical areas. SDI is a composite average of the rankings of the gross domestic product per capita, average educational attainment among individuals aged older than 15 years, and fertility rates among females under the age of 25 years and is expressed on a scale of 0–1^19,20^. The SDI of each province was provided in Supplementary Table 1. All estimates were reported as point estimations with their 95% uncertainty intervals (UI).

In addition, the mortality to incidence ratio (MIR), which is an index of quality of care and indicator of survival, was calculated and compared in 31 provinces as well as the whole country^21^. GBD 2019 data analysis was performed by Stata version 13 and R version 3.5.0^22,23^.

## Results

### The level and trend of the burden of T1DM in Iran from 1990 to 2019

**Figure 1:**
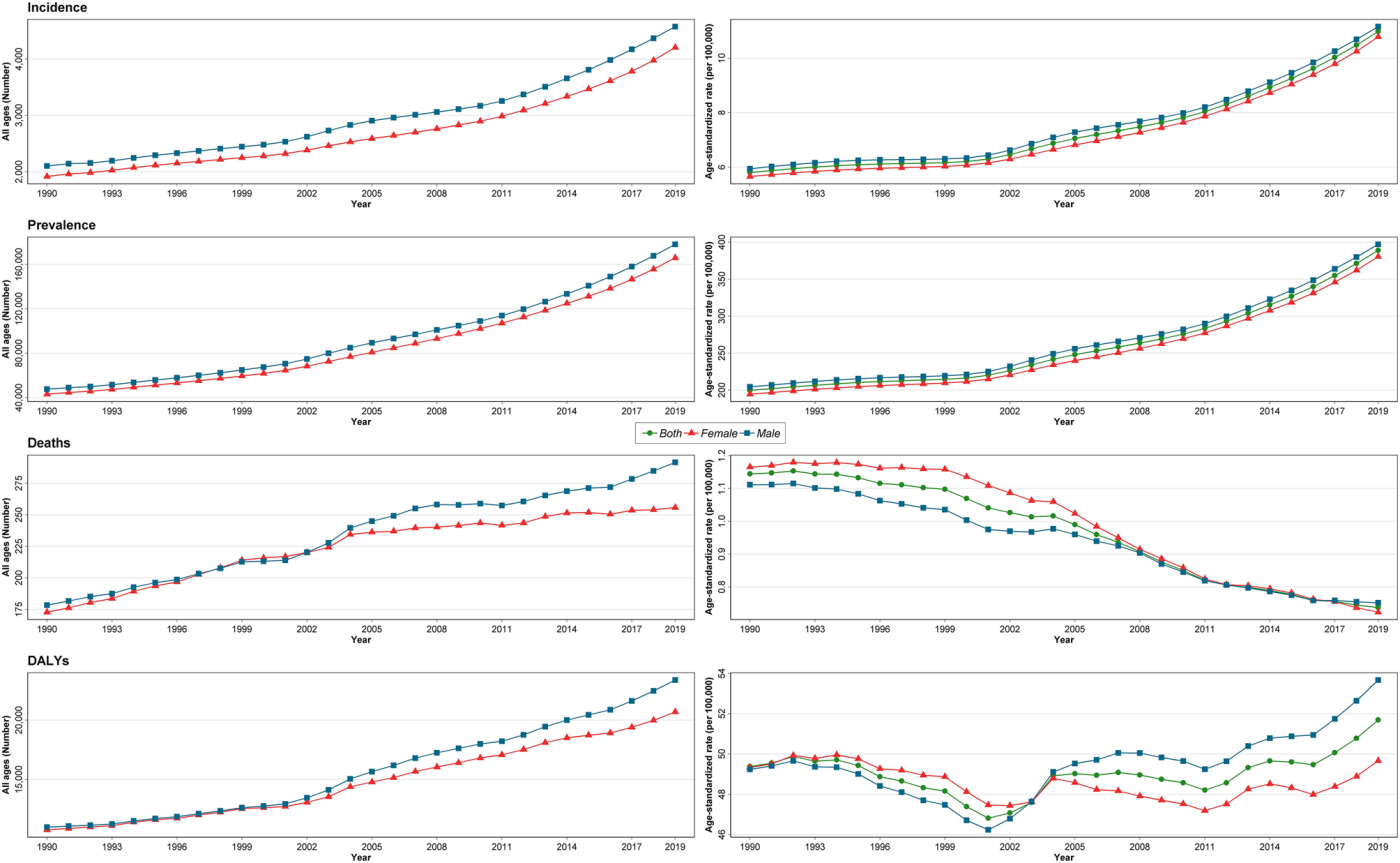
Time trend of T1DM burden by all ages number and age-standardized rate by sex in the country from 1990 to 2019.

All age numbers and age-standardized rates of incidence and prevalence of T1DM increased from 1990 to 2019 in the country in males, females, and both sexes. While the number of deaths had a similar increasing trend, the death rates decreased from 1990 to 2019 (Fig. 1). The age-standardized incidence rate of T1DM for both sexes in Iran increased from 5.8 (95% UI: 4.7–7.2) in 1990 to 11.0 (8.9–13.5) per 100,000 in 2019 (Table 1, Supplementary Table 2, and Fig. 1). The prevalence rate of T1DM in both sexes increased from 199.6 (157.2–250.0) in 1990 to 388.9 (306.1–482.1) per 100,000 in 2019 (Table 1, Supplementary Table 2, and Fig. 1). During the same period, the death rate of T1DM in both sexes decreased from 1.1 (0.8–1.3) to 0.7 (0.6–0.8) per 100,000 (Table 1, Supplementary Table 2, and Fig. 1).

**Table 1:** All ages number and age-standardized rate (per 100,000), Iran.

Measure	Metric	Year						% Change (1990–2019)		
		1990			2019					
		Both	Female	Male	Both	Female	Male	Both	Female	Male
Incidence	All ages number	4020 (3151–5047)	1917 (1496–2409)	2103 (1658–2641)	8780 (6990–10,897)	4205 (3363–5206)	4575 (3639–5669)	118.4 (98.9–142.8)	119.4 (99.6–144.5)	117.5 (98.5–141.9)
	Age-standardized rate (per 100,000)	5.8 (4.7–7.2)	5.7 (4.6–7)	5.9 (4.8–7.3)	11 (8.9–13.5)	10.8 (8.7–13.3)	11.2 (9–13.7)	89.3 (84.8–93.8)	90.6 (85.5–95.9)	87.9 (82.9–93.2)
Prevalence	All ages number	90,635 (70,205–114,686)	43,082 (33,297–54,832)	47,552 (36,960–59,912)	344,242 (270,789–427,806)	166,040 (130,459–207,901)	178,202 (139,793–221,505)	279.8 (260.5–300.7)	285.4 (264.2–308.9)	274.7 (253.6–294.9)
	Age-standardized rate (per 100,000)	199.6 (157.2–250)	194.5 (151.8–244.3)	204.3 (161.2–254.3)	388.9 (306.1–482.1)	380.4 (297.9–473.8)	397.1 (312–489.2)	94.9 (89.5–99.9)	95.5 (89.7–101.2)	94.4 (88.7–100.5)
Deaths	All ages number	351 (275–402)	173 (129–208)	178 (139–216)	547 (441–602)	256 (192–300)	292 (230–333)	55.9 (29.4–96.6)	48 (15.4–102.1)	63.5 (25–106)
	Age-standardized rate (per 100,000)	1.1 (0.8–1.3)	1.2 (0.8–1.4)	1.1 (0.8–1.4)	0.7 (0.6–0.8)	0.7 (0.6–0.9)	0.8 (0.6–0.9)	− 35.6 (− 46.7–− 13.2)	− 37.9 (− 52–− 7.9)	− 32.3 (− 49.3–− 7.7)
DALYs	All ages number	21,730 (18,358–25,532)	10,753 (8780–12,785)	10,977 (9195–13,292)	44,086 (34,612–56,048)	20,699 (15,941–26,479)	23,387 (18,341–29,594)	102.9 (66.5–137.3)	92.5 (56.2–132.1)	113.1 (72.5–148.3)
	Age-standardized rate (per 100,000)	49.4 (40.1–58.1)	49.3 (39–59.4)	49.2 (39.9–59.5)	51.7 (40.9–65.1)	49.7 (39–63.3)	53.7 (42.3–67.5)	4.7 (− 11.2–23.3)	0.7 (− 17.4–24.6)	9 (− 9.7–28.7)
YLLs	All ages number	15,673 (13,325–18,055)	7851 (6286–9317)	7822 (6527–9609)	19,375 (15,395–21,137)	8608 (5871–9828)	10,766 (8672–12,058)	23.6 (− 5.1–49.5)	9.6 (− 23–39.4)	37.6 (2–66.7)
	Age-standardized rate (per 100,000)	34.7 (27.3–39.6)	34.9 (25.9–42)	34.4 (26.9–41.6)	23.5 (18.9–25.6)	21.8 (15.5–24.8)	25.1 (20–28.2)	− 32.4 (− 44.9–− 14.8)	− 37.6 (− 53.3–− 15.4)	− 26.9 (− 43.4 to − 8.1)
YLDs	All ages number	6057 (3977–8944)	2902 (1905–4278)	3155 (2071–4649)	24,711 (16,183–36,137)	12,091 (7822–17,663)	12,620 (8199–18,732)	308 (283.3–331)	316.7 (288.9–345.6)	300 (275.4–325)
	Age-standardized rate (per 100,000)	14.7 (9.5–21.5)	14.4 (9.5–21.1)	14.9 (9.7–21.9)	28.2 (18.6–41.4)	27.9 (18.3–40.7)	28.6 (18.7–42.2)	92.5 (86.4–98.9)	93.3 (85.6–101.7)	92.1 (84.3–100.5)

Data in parentheses are 95% UIs.

The trend of age-standardized DALYs rate was relatively descending while its absolute number was ascending (Fig. 1, Table 1, and Supplementary Table 2).

T1DM YLDs in the country in both sexes increased from 14.7 (9.5–21.5) in 1990 to 28.2 (18.6–41.4) per 100,000 in 2019, with three steps upward climb in the ranking (Table 1, Supplementary Table 2, and Supplementary Fig. 1A).

### The sub-national burden of T1DM from 1990 to 2019

**Figure 2:**
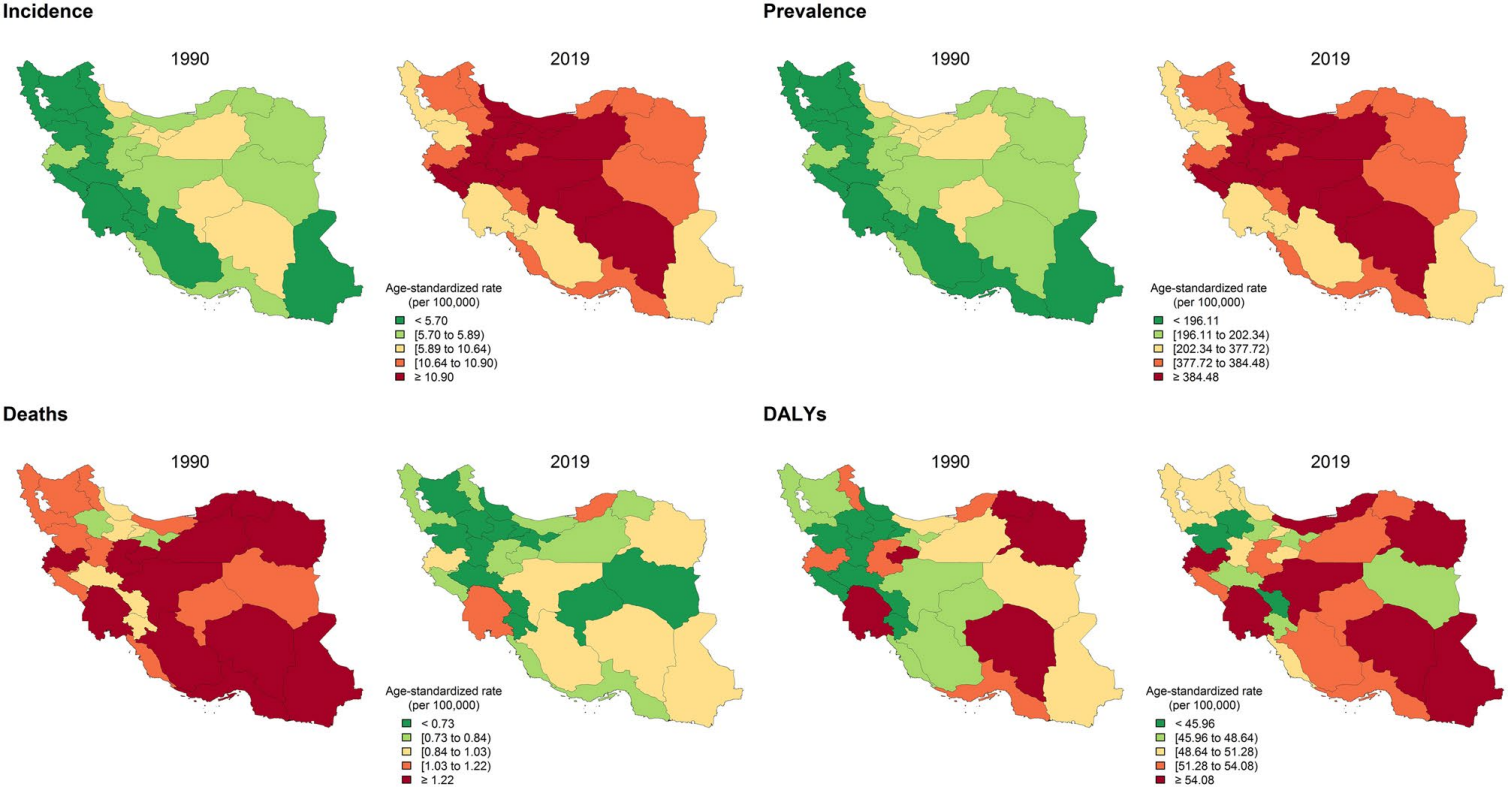
The map of Iran by the measures of incidence, prevalence, deaths, and DALYs of T1DM in 1990 and 2019 in 31 provinces.

The trend of T1DM prevalence in all 31 provinces ascended during 1990–2019. The highest age-standardized prevalence rate of T1DM in both sexes from 1990 to 2019 was in Tehran province (212.7 [167.3–264.6] in 1990 and 414.4 [328.8–515.8] in 2019) constantly, and the lowest rate in 1990 and 2019 was in Fars (179.8 [138.7–228.4] in 1990) and Sistan and Baluchistan (349.2 [274.2–433.5] in 2019), respectively (Supplementary Fig. 1B). The map of Iran by the measures of incidence, prevalence, deaths, and DALYs of T1DM in 1990 and 2019 in 31 provinces is depicted in Fig. 2.

The trend of age-standardized death rate descended in the country from 1990 to 2019. The highest death rate due to T1DM in both sexes from 1990 to 2019 was in Khuzestan province, with a fixed first rank and descending number (1.6 [0.8–2.2] in 1990 and 1.2 [0.6–1.5] in 2019) and the lowest was in Tehran province again with a fixed rank and descending number (0.8 [0.4–1] in 1990 and 0.5 [0.2–0.6] in 2019) (Supplementary Fig. 1C).

Regarding the T1DM age-standardized DALYs rate from 1990 to 2019, among 31 provinces, the highest rate of DALYs in T1DM in both sexes from 1990 to 2019 was in Khuzestan province, with the fixed first rank (64.8 [42.2–82.2] in 1990 and 66.2 [45.5–83] in 2019). Conversely, the lowest rates were in Zanjan (36.7 [29.3–50.7] in 1990 and 41.9 [31.6–55] in 2019) and Chaharmahal Bakhtiari (39.9 [32–50.1] in 1990 and 44.4 [34.3–58.3] in 2019) provinces with fixed last ranks (Supplementary Fig. 1D).

Among 31 provinces, the greatest T1DM age-standardized rate for YLDs in both sexes was in Alborz from 1990 to 2019, while the least YLDs rates were in Fars (1990) and Sistan and Baluchistan (2019) (Supplementary Fig. 1A).

### The burden of T1DM by socio-demographic index (SDI) from 1990 to 2019

**Figure 3:**
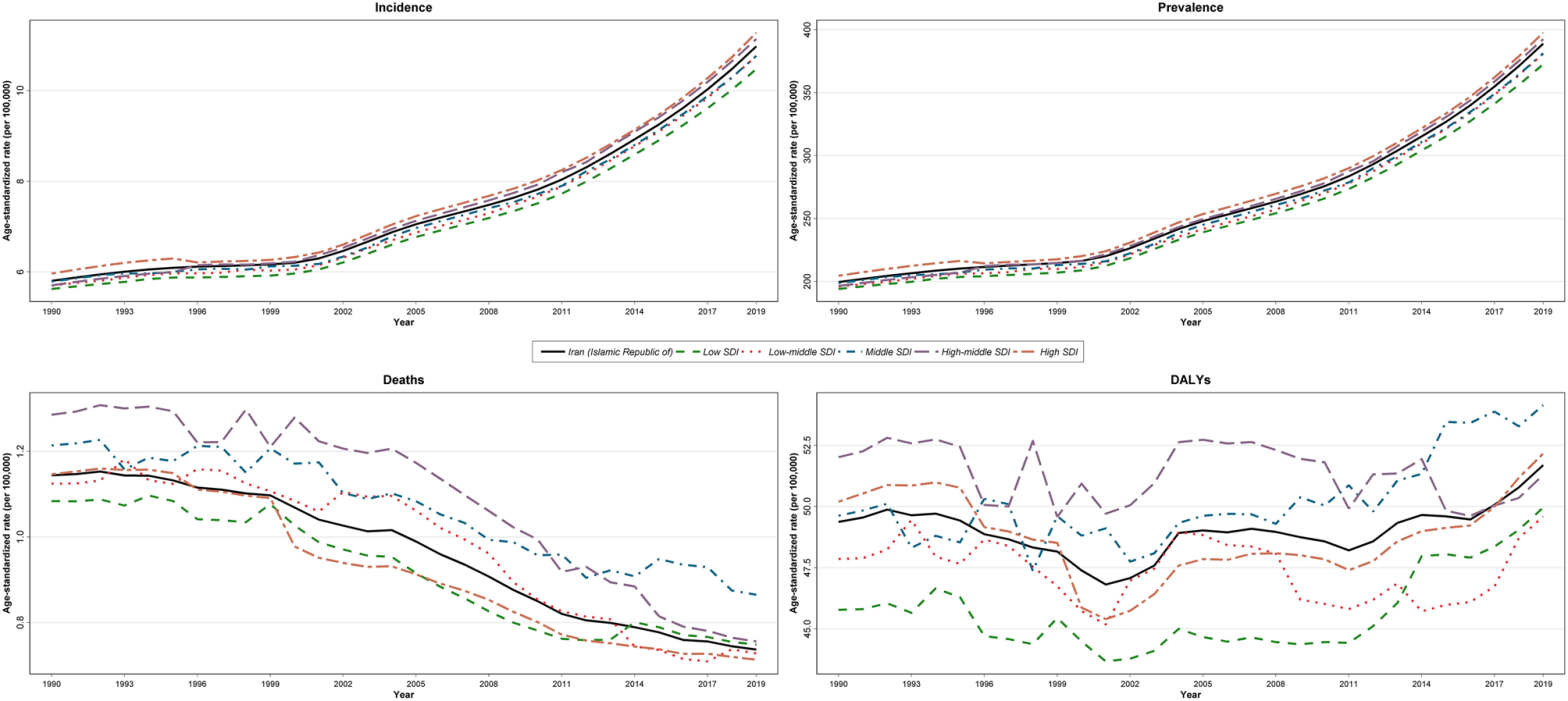
Time trend of age-standardized T1DM burden by SDI quintiles in the country from 1990 to 2019.

The trends of age-standardized incidence and prevalence rates of T1DM were ascending in all SDI groups. In low, low-middle, and middle SDI quintiles, incidence and prevalence rates were slightly below, while high-middle and high SDI quintiles were slightly above the average of the country (Fig. 3). The SDI values among 31 provinces were reported in Supplementary Table 1.

The trend of the age-standardized death per in 100,000 of T1DM was descending in all SDI groups particularly since 2002 that reduced rapidly. In low and high SDI death rate was slightly below average and in low-middle, middle and high-middle SDI slightly above the average of the country. The decrease in death rate in high SDI increased sharply since 1999 and dropped below the national average while the death rate in low SDI increased and reached to national average since 2014 (Fig. 3).

The trend of age-standardized DALYs rate was slightly descending till 2000 and after that increased with slight slope until 2004 that experienced a small fall till 2011 and then increased with a steep slope till 2019 with a small fall in 2016. However, it had no steady trend and had frequent fluctuations in average and in all SDI groups (Fig. 3).

### Percent changes

**Figure 4:**
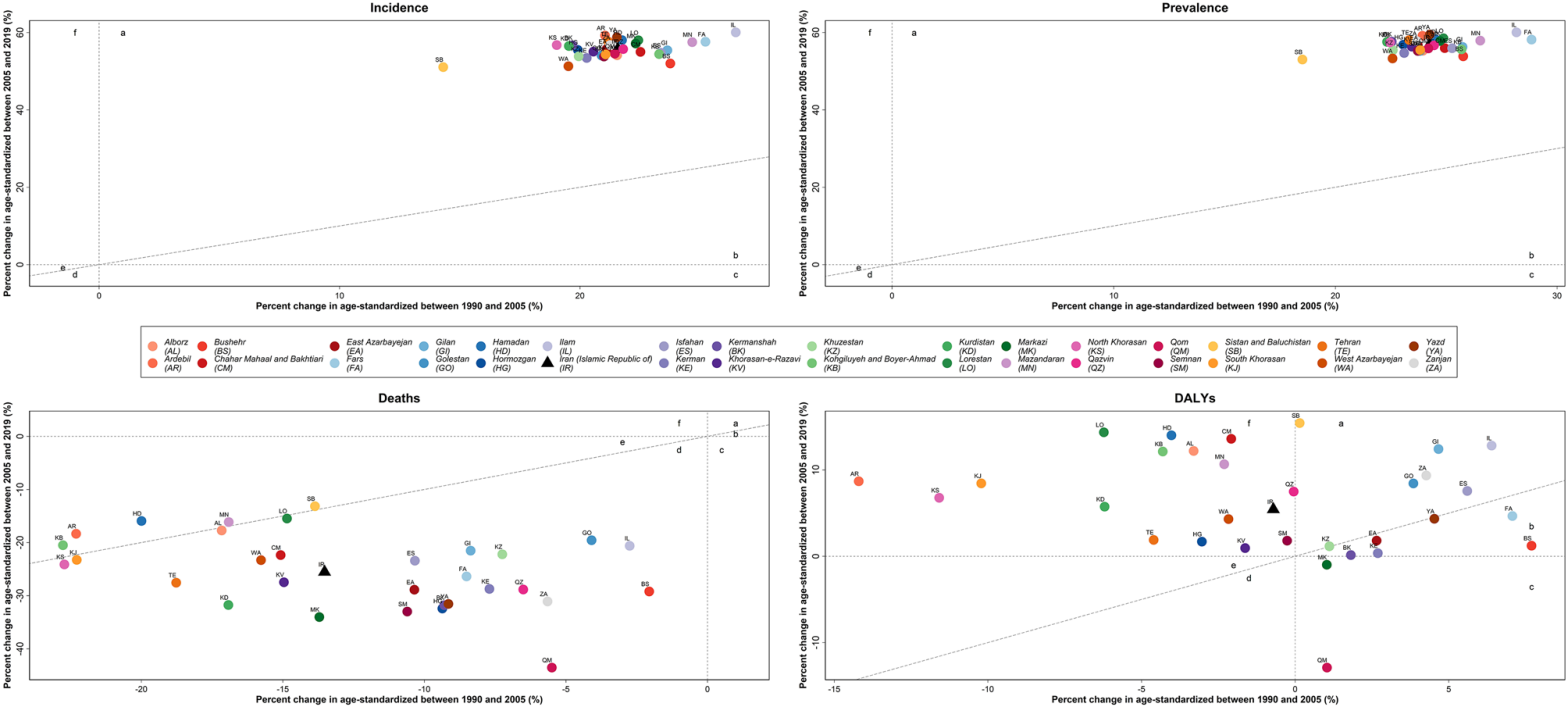
National and sub-national percent change in the age-standardized burden of T1DM in 1990–2005 compared to 2005–2019 (**a**), rates increased more rapidly after vs before 2005. (**b**) Rates increased more slowly after vs before 2005. (**c**) rates increased before 2005 but decreased after 2005. (**d**) rates decreased more rapidly after than before 2005. (**e**) rates decreased more slowly after than before 2005. (**f**) rates decreased before 2005 but increased after 2005.

Percent changes of T1DM age-standardized incidence and prevalence rates in all 31 provinces and national level had the same pattern (zone a: age-standardized rates increased more rapidly after vs before 2005). Assessing the percent changes of age-standardized death rates across provinces revealed that in five provinces rates decreased more slowly after than before 2005 (Sistan and Baluchistan, Mazandaran, Hamadan, Ardebil, and Kermanshah) but in other 26 provinces and national level, rates decreased more rapidly after than before 2005 (zone d). The national and provincial percent changes in the age-standardized DALYs rate had four different patterns but most provinces and national level were included in zone f (rates decreased before 2005 but increased after 2005) (Fig. 4, Supplementary Table 3).

### Age distribution of T1DM

**Figure 5:**
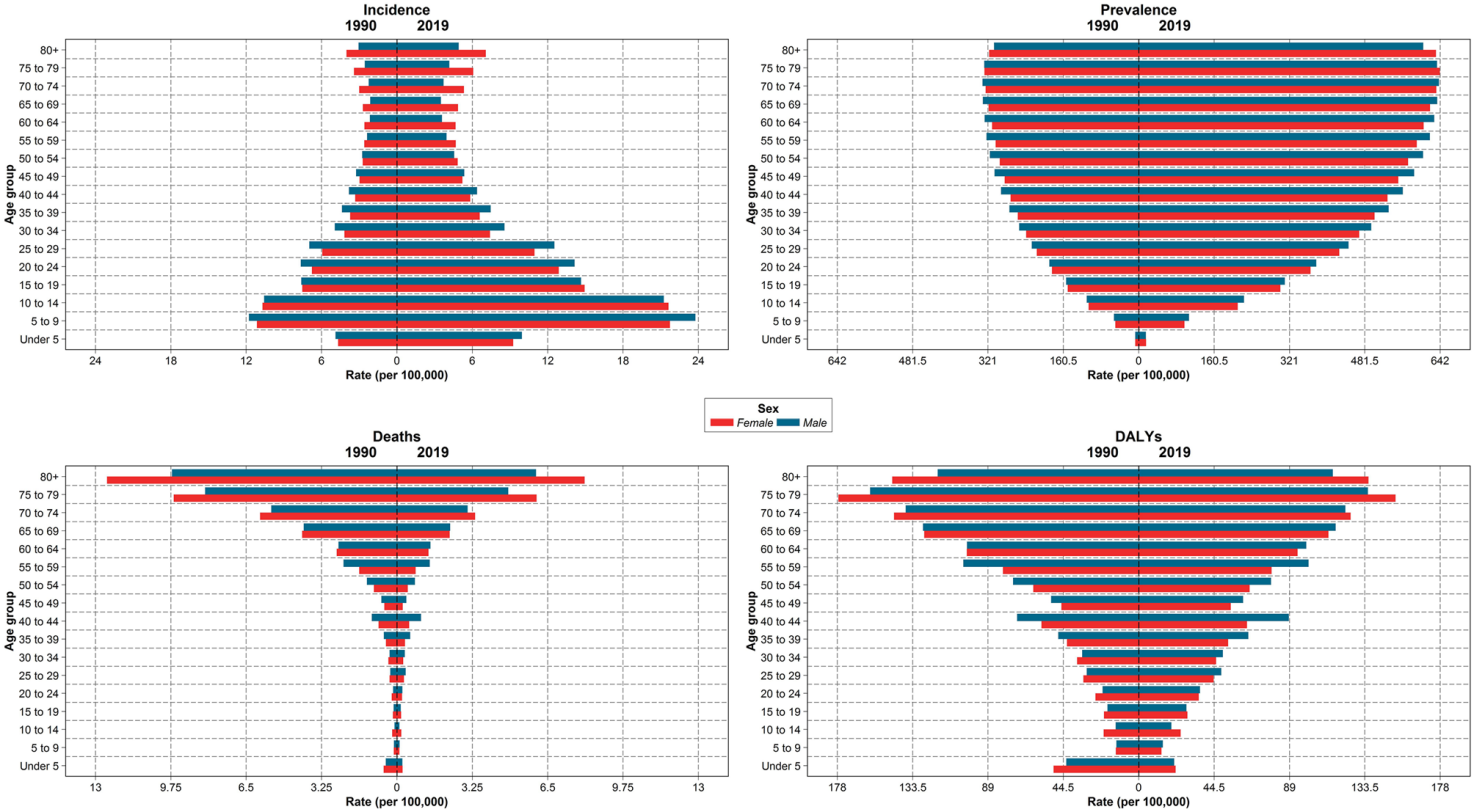
Burden of T1DM in the country by sex and age groups in 1990 and 2019.

The highest incidence rate for T1DM was in the 5–9 years age group in males and females. Also, the highest prevalence, death, and DALYs rates were in 75–79, above 80, and 75–79 years, respectively, in males and females (Fig. 5).

### Factors affecting T1DM burden from 1990 to 2019

By decomposition analysis, factors affecting trends and change in the new cases of T1DM were extracted in different provinces in Iran in males and females. The overall percent change in the incidence of T1DM in Iran was 118.4%. About 44% of this rise in the number of T1DM cases was attributed to population growth, 101.9% to incidence rate change, and − 27.5% to age structure change (Table 2). Decomposition analysis of T1DM new cases at national and sub-national levels by sex has been shown in Supplementary Table 4.

**Table 2:** Decomposition and the percent changes in T1D incidence number and its causes in 31 provinces in both genders.

Location	New cases		Expected new cases in 2019		% 1990–2019 new cases change cause			% 1990–2019 new cases overall change (%)
	1990	2019	Population growth	Population growth + Aging	Population growth (%)	Age structure change (%)	Incidence rate change (%)	
Iran (Islamic Republic of)	4020	8780	5789	4682	44	− 27.5	101.9	118.4
Subnational								
Alborz	109	314	214	170	95.4	− 40	131.8	187.2
Ardebil	81	131	89	68	10.9	− 26.1	77.4	62.2
Bushehr	50	136	86	73	71.4	− 27	126.9	171.3
Chahar Mahaal and Bakhtiari	51	105	70	56	35.3	− 27	96.3	104.6
East Azarbayejan	235	410	278	220	18.2	− 24.7	81	74.5
Fars	235	472	319	244	35.9	− 32.1	97.1	100.9
Gilan	162	263	181	139	11.6	− 25.5	76.7	62.7
Golestan	95	207	137	111	43.9	− 26.5	100.5	117.9
Hamadan	114	176	118	93	3.4	− 22.6	73.2	53.9
Hormozgan	64	210	132	113	105	− 29.6	151.8	227.2
Ilam	32	65	42	32	32	− 30.5	103.2	104.7
Isfahan	268	546	368	290	37.1	− 29.1	95.5	103.5
Kerman	134	369	239	200	78.3	− 29.1	125.4	174.6
Kermanshah	119	199	138	107	16.3	− 26.3	76.8	66.8
Khorasan-e-Razavi	334	724	472	389	41.4	− 24.7	100.4	117.1
Khuzestan	227	521	345	283	52.2	− 27.3	105	129.9
Kohgiluyeh and Boyer-Ahmad	34	79	52	42	51.9	− 29.6	109.6	132
Kurdistan	85	164	114	89	33.8	− 29.6	88.4	92.6
Lorestan	108	187	123	97	14.3	− 24.3	83.3	73.3
Markazi	84	148	100	78	19.2	− 26.6	83.5	76.1
Mazandaran	182	361	239	186	31.5	− 29.4	96.2	98.3
North Khorasan	43	94	59	50	37.5	− 21.1	100.7	117.1
Qazvin	67	136	93	73	39.9	− 31	95.5	104.4
Qom	52	145	96	78	84.1	− 34.5	129.4	179
Semnan	34	80	53	44	54.9	− 26.6	106.9	135.2
Sistan and Baluchistan	103	339	208	191	101.9	− 16.9	143.7	228.8
South Khorasan	46	92	57	49	24.2	− 17.7	92.4	98.9
Tehran	601	1521	985	808	63.8	− 29.4	118.5	152.9
West Azarbayejan	159	342	233	190	46.6	− 26.9	95.4	115.2
Yazd	49	133	82	69	69.2	− 26.9	131.4	173.6
Zanjan	62	110	75	58	21.6	− 27.4	83	77.1

### Quality of T1DM care

**Figure 6:**
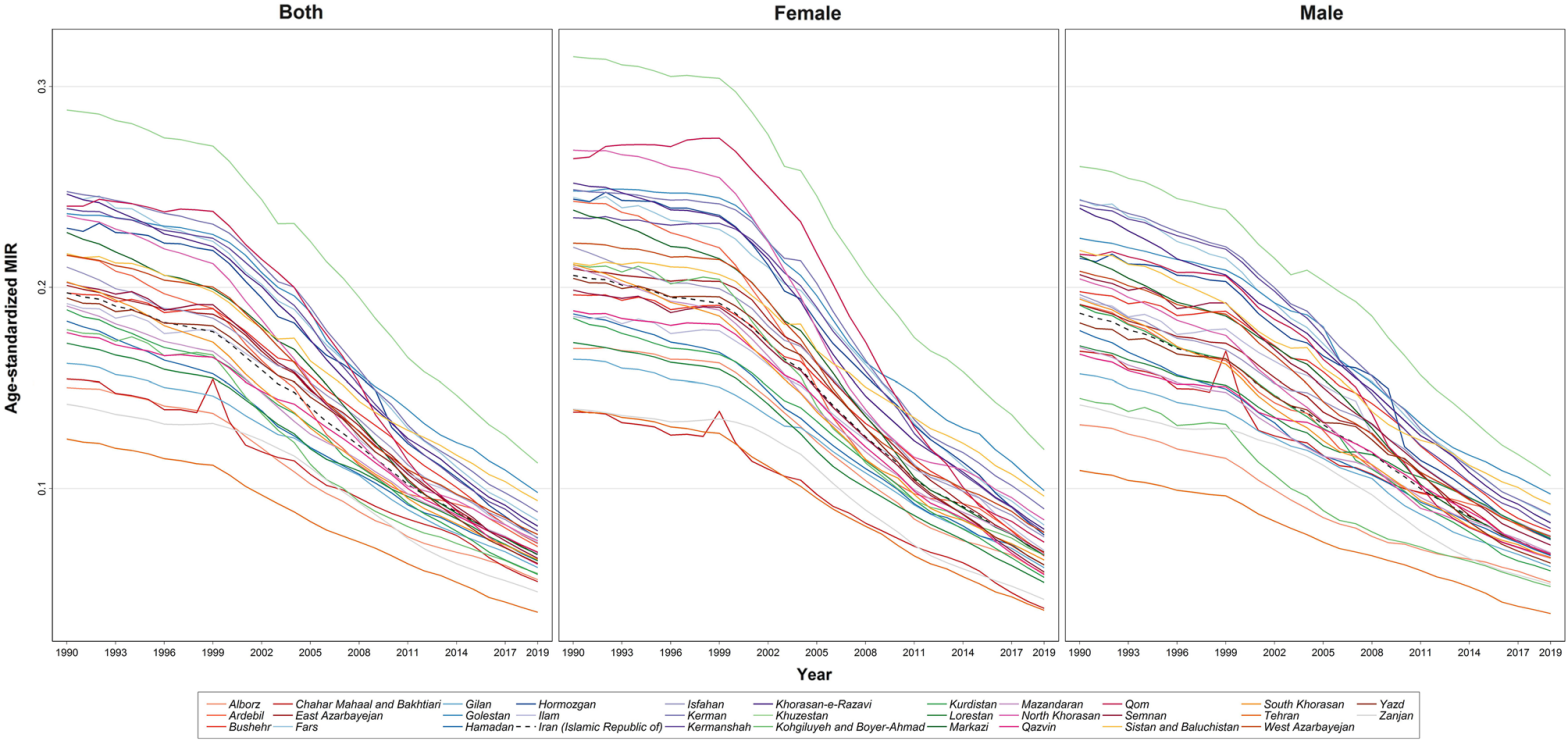
Time trend of age-standardized MIR due to T1DM in the country from 1990 to 2019.

**Figure 7:**
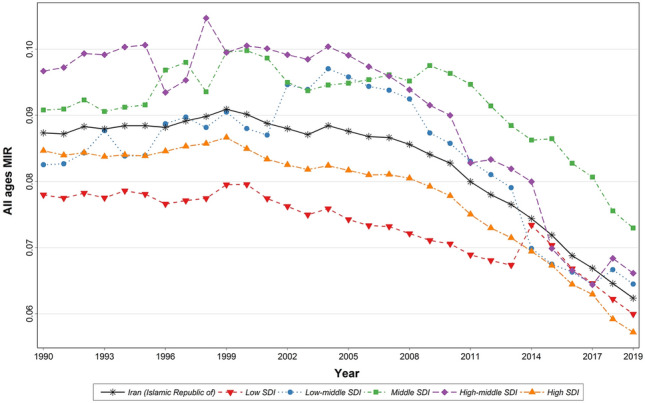
Time trend of all ages MIR due to T1DM by SDI quintiles in the country from 1990 to 2019.

According to GBD data, the MIR number reduced in all provinces in both males and females from 0.087 in 1990 to 0.062 in 2019, which indicates the improving quality of T1DM care and survival during the study period (Fig. 6). Another analysis showed that MIR has reduced in all SDI groups during the past 30 years as well. However, MIR in low and high SDI provinces was below the country’s average curve. The MIR curve remained constant and relatively plateau until 2002, and after that, it decreased with a moderate slope until 2012, and then it dropped again with a steeper slope (Fig. 7).

## Discussion

This study showed the status and trend of the T1DM burden in 31 provinces of Iran during the past 30 years (1990–2019). The age-standardized rates and all ages numbers of incidence and prevalence of T1DM in most provinces, as well as the whole country, increased dramatically in recent ten years. The increasing trend of incidence and prevalence of diabetes were also reported in other studies^24,25^. However, during the same period, death rates were reduced in all provinces except for one. Age-standardized DALYs rate reduced in most provinces and increased in some provinces and had no similar trend. According to the IDF, the rates of new cases of T1DM in 0–14 years old children in Iran were similar without changes in 2010 and 2019 (0.7)^2^, and the estimated rate of T1DM in 0–19 years old children was 8.2 per 100,000 in 2021^10^. While according to the GBD’s results, the rate of new cases of T1DM was 5.8 [4.7–7.2] in 1990 and 11 [8.9–13.5] in 2019.

GBD data showed that the incidence rate of T1DM has approximately doubled from 1990 to 2019 in Iran and in most of its provinces, while during the same time death rate reduced by one-third. In a previous study, the incidence rate of type 1 and 2 diabetes was increased by 102%^24^. However, when T1DM was analyzed separately, although the number of incident cases increased by about one-third, the age-standardized incidence rate was constant with no changes^24^. Despite such changes in our analysis, DALYs increased during the study period, especially after 2011 and 2016.

Gale’s study indicated that from the 1960s–1990s incidence of T1DM has increased by 2–4% each year^26^, which is similar to 1990–2019 in the current study that the incidence has doubled during the past 30 years.

The cause of such increases in the incidence and prevalence may be due to the improved health care coverage that leads to better registration and recording of T1DM cases.

Several maternal and perinatal risk factors, including increased maternal age^27,28^, tea drinking during pregnancy^29^, maternal pre-eclampsia^28–30^ and infections^29^, higher birth weight^30,31^, delivery by Caesarean section^31,32^, premature rupture of membranes^31^, maternal urinary tract infection during pregnancy^31^, gestational diabetes^30^, preterm birth^28^ and neonatal infectious^28^, have been identified for TIDM. Although, there is no consistency among different studies about these risk factors and some other studies did not confirm them^31,33^. Among the mentioned risk factors, a small percentage of the increase in the incidence of childhood T1DM in the past decades may be attributed to the increase in maternal age, as was shown in Ayati et al. study^30^. According to the latest study in our country, gestational diabetes, pre-eclampsia, and birth weight of more than 4 kg, but not maternal age, were identified as risk factors for developing T1DM in the offspring in the future^30^. According to the decomposition analysis (Table 2), 44% of new cases increment during the study period (1990–2019) was due to the population growth.

In addition, stress which is one of the trigger factors of T1DM onset has increased in the past decades, which may cause increased incidence and prevalence of T1DM. Also, the increase in other environmental risk factors such as new viruses, more prevalent cow's milk and formula consumption, and processed foods with higher amounts of advanced glycation end-products and nitrites or N-nitroso compounds in processed meat products, may have contributed to such increased incidence and prevalence of T1DM^34–37^.

Regarding the age distribution of T1DM, as it was predicted, the greatest incidence rate was in ages 5–9 years. The highest prevalence of T1DM was observed in ages between 75 and 79 years due to improved diabetes care, availability of insulin, and increased survival of T1DM patients, that cause most patients to reach older ages.

In the current study, age-standardized incidence rates of T1DM in high and high-middle SDI were higher than in the low and low-middle SDI groups, which is similar to the worldwide population in Liu et al. study^24^. In our country, the increasing incidence of T1DM after 2000 continued with a steeper slope, and it was similar in all SDI groups. But in Liu's study, age-standardized incidence rates increased across all four SDI regions, with the greatest increase in the high SDI region and was stable in the low SDI regions^24^. This rapid growing incidence of T1DM may be attributed to changing diagnostic criteria for T1DM in 1997^38^ that reduced FBS threshold criteria from 140 to 126 mg/dl and shifting from the oral glucose tolerance test to fasting plasma glucose or may be due to improving health care and T1DM patients detection in the country.

It seems that in deprived areas (low SDI), by increasing incidence and prevalence of T1DM, due to limited access to medications and treatment, the number of YLLs and consequently DALYs has increased, which is contradictory to the reduced death rates. It may be explained that by death reduction, YLDs increase and affect DALYs more than reduced YLLs. In other provinces, although incidence and prevalence of T1DM have increased, due to adequate access to treatment and health care facilities, death and consequently DALYs has reduced and DALYs has affected more by reduced YLLs than increased YLDs. In Qom province with the fastest death rate reduction, also the highest speed of DALYs reduction was observed.

Higher YLLs than YLDs in 1990 were due to the higher rate of T1DM deaths, and the higher YLDs than YLLs in 2019 were due to the reduction of the T1DM deaths rate. The T1DM death rate reduction was the result of the improvement of T1DM care by the introduction and increased availability of insulin pens and its application in the treatment of T1DM during the last five years. However, YLLs decreased, and YLDs increased in all provinces except for three provinces. The cause of higher YLLs in Sistan and Baluchestan, Khuzestan, and Golestan than YLDs was the higher death rates in these provinces (first, second, and fourth death rates in 2019). Sistan and Baluchistan was categorized as a low SDI province, but Khuzestan and Golestan had higher SDIs in 2019.

For the age groups under 20, both in 1990 and 2019, the YLLs were higher than the YLDs, but in 1990 due to the higher death rate, YLLs were higher than YLDs, and in 2019 due to the improvement of T1DM treatment, YLDs increased although yet it was lower than the YLLs.

For ages above 20, YLLs were more than YLDs in 1990, but in 2019 YLDs were higher than YLLs except for a few provinces, including Sistan and Baluchistan, Khuzestan, and Golestan.

As the onset of T1DM is in childhood and early adulthood, YLLs are more because of death in childhood results in a higher loss of life years. During the study period although YLLs reduced due to the reduction of T1DM death rate DALYs increased. This indicates that DALYs have moved in the same direction as YLDs and have been affected more by YLDs instead of YLLs.

Considering population growth and aging, the DALYs count was increasing during the study period, but age-standardized DALYs were almost steady during the past 30 years, and only a few small fluctuations were observed in the trend of DALYs that may be attributed to political changes in the country and changes in health care policy and strategies.

In this study, MIR reduced from 2002 to 2019, which may be due to either improvement in survival (decreasing mortality) or improvements in the diagnosis of T1DM (increasing in the reported incidence), or a combination of both. Death rates and MIRs in the low SDI group increased after 2014 and reached the country average, but at the same time, death rates and MIRs in the low-middle SDI and high-middle SDI group fell below the country average after 2014. However, after 2017 the curve for low-middle and high-middle SDI groups returned to above the country average (Fig. 7). After 2014 the distance between all SDI curves reduced, and the curves came close together. In 2019, the least MIR, which was near zero, was observed in the high-SDI group, and the highest MIR (between 0.007 and 0.08) was in the middle SDI group as well. Such MIR reduction in the high SDI group of T1DM patients may be due to improving diabetes care, accessibility, and increased insurance coverage and availability of drugs such as insulin pens in the country. However, at the same time, MIRs increased in low SDI groups unexpectedly (while it was expected to reduce, like high SDI group), which can be explained with improved diabetes care coverage and accessibility (mainly due to establishment and increased number of health houses throughout the country) that caused improved case registry and records especially in low-income and remote areas.

After 2014, DALYs increased or remained unchanged in most SDI groups but only reduced in low-middle and high-middle SDI groups because the death rate in these groups reduced after 2014, significantly which caused YLLs and DALYs reduction. Also, during the same time distance between the trend of number and age-standardized rate of T1DM death and DALYs reduced significantly and reached each other.

In all provinces, age structure changes and population aging had a noticeable effect on the incidence of T1DM. Also, the contribution of population growth in the change of T1DM incidence rate was lower than the incidence rate change in all provinces according to decomposition analysis. Change in incidence rate may be indicative of improved health care coverage in the country that caused improved case findings. Age structure change may be due to the migration or the aging of the population.

This study has limitations. The overall quality of burden estimates were according to the accuracy of data sources used in the modeling. In addition, a revision on the T1DM risk factors’ selection is highly recommended as in the current format, only high fasting plasms glucose and non-optimal temperature were included, while the list can be improved.

## Conclusion

In conclusion, the analysis of GBD data in Iran with 31 provinces showed that the age-standardized incidence and prevalence rate of T1DM increased from 1990 to 2019, while the death rate decreased and the DALYs rate was steady during the same period.

MIR has reduced all over the country in all SDI groups since 2002, especially from 2014 to 2019. This means that the quality of T1DM care has improved in the country since 2014, significantly.

## Supplementary Information

Supplementary Information 1.

Supplementary Information 1.

Supplementary Information 2.

Supplementary Information 3.

Supplementary Information 4.

Supplementary Information 6.

Supplementary Information 7.

Supplementary Information 8.

Supplementary Information 9.
