# Supplementary material for: National and sub-national burden and trend of type 1 diabetes in 31 provinces of Iran, 1990–2019
Source: Sci Rep. 2023 Mar 14;13:4210. doi: 10.1038/s41598-023-31096-8 (PMC10014831; doi:10.1038/s41598-023-31096-8)
Supplement: Supplementary file 1 — Supplementary Information 1. [file 41598_2023_31096_MOESM1_ESM.docx]

**Supplementary Figure 1A:** Age-standardized YLDs rate of T1DM at national and sub-national levels, 1990 compared to 2019

**Supplementary Figure 1B:** Age-standardized prevalence rate of T1DM at national and sub-national levels, 1990 compared to 2019

**Supplementary Figure 1C:** Age-standardized death rate of T1DM at national and sub-national levels, 1990 compared to 2019

**Supplementary Figure 1D:** Age-standardized DALYs rate of T1DM at national and sub-national levels, 1990 compared to 2019

**Supplementary Table 1:** SDI values of each province in 1990 and 2019

**Supplementary Table 2:** All ages number and age-standardized rate (per 100,000) of T1DM burden by sex with percent change, Sub-national

**Supplementary Table 3:** National and sub-national age-standardized burden of T1DM in 1990, 2005, and 2019 with percent change in 1990 to 2005 compared to 2005 to 2019

**Supplementary Table 4:** Decomposition analysis of T1DM new cases at national and sub-national levels by sex
