# Supplementary figures and images for: National and sub-national burden and trend of type 1 diabetes in 31 provinces of Iran, 1990–2019
Source: Sci Rep. 2023 Mar 14;13:4210. doi: 10.1038/s41598-023-31096-8 (PMC10014831; doi:10.1038/s41598-023-31096-8)

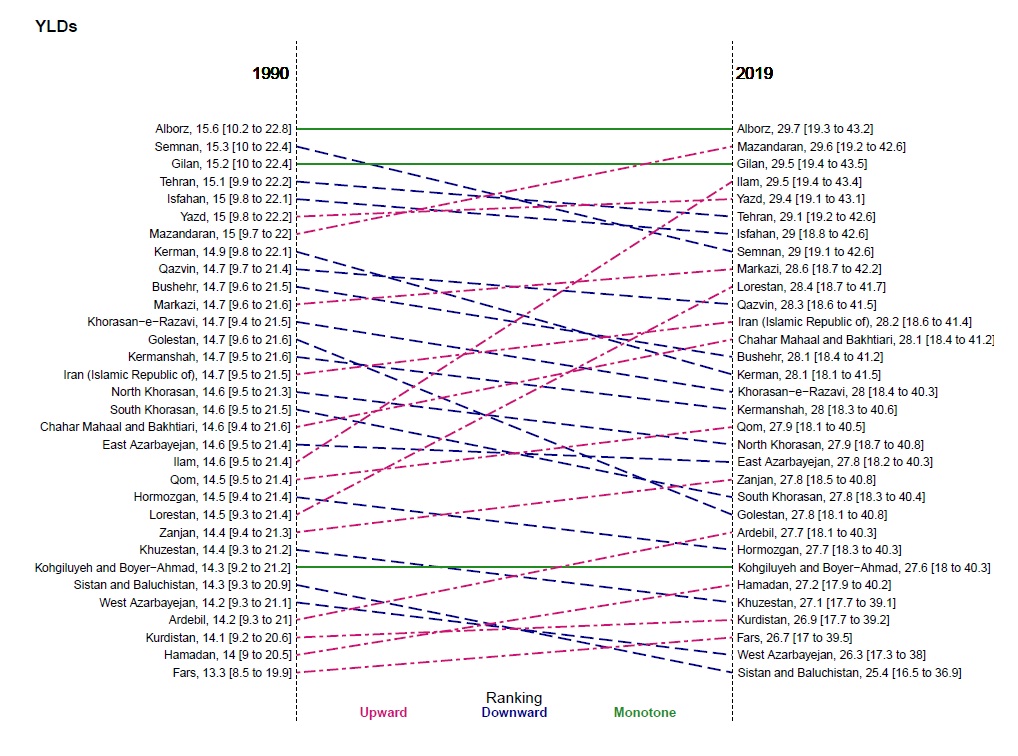

Supplement: Supplementary file 2 — Supplementary Information 1. [file 41598_2023_31096_MOESM2_ESM.jpg]

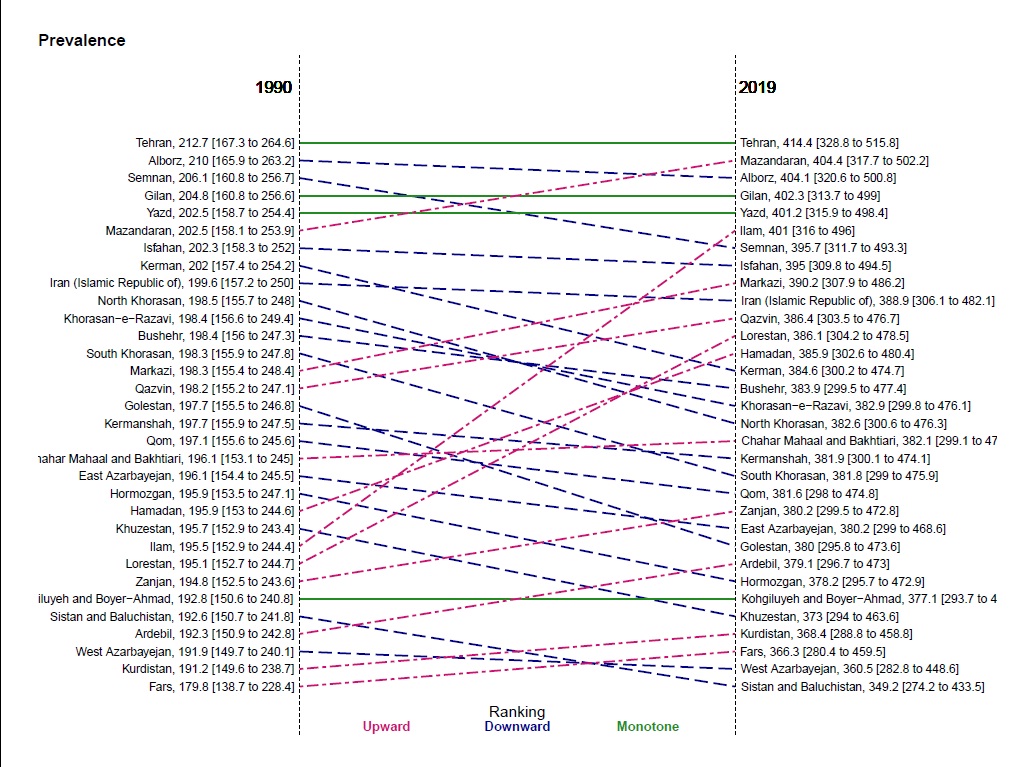

Supplement: Supplementary file 3 — Supplementary Information 2. [file 41598_2023_31096_MOESM3_ESM.jpg]

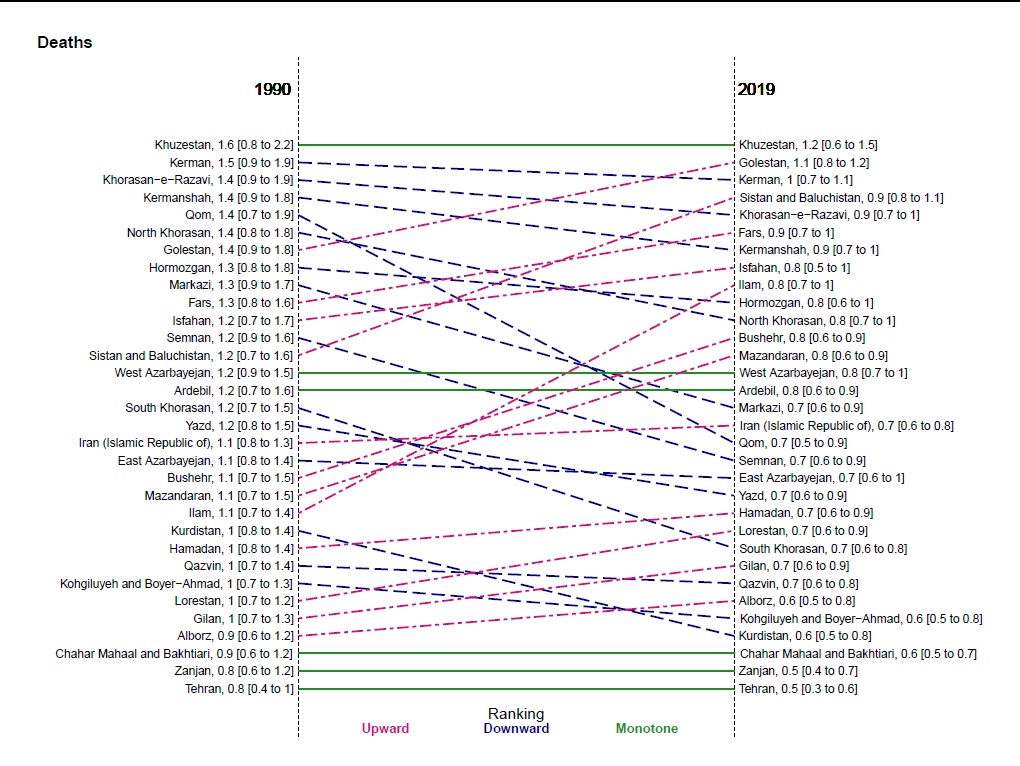

Supplement: Supplementary file 4 — Supplementary Information 3. [file 41598_2023_31096_MOESM4_ESM.jpg]

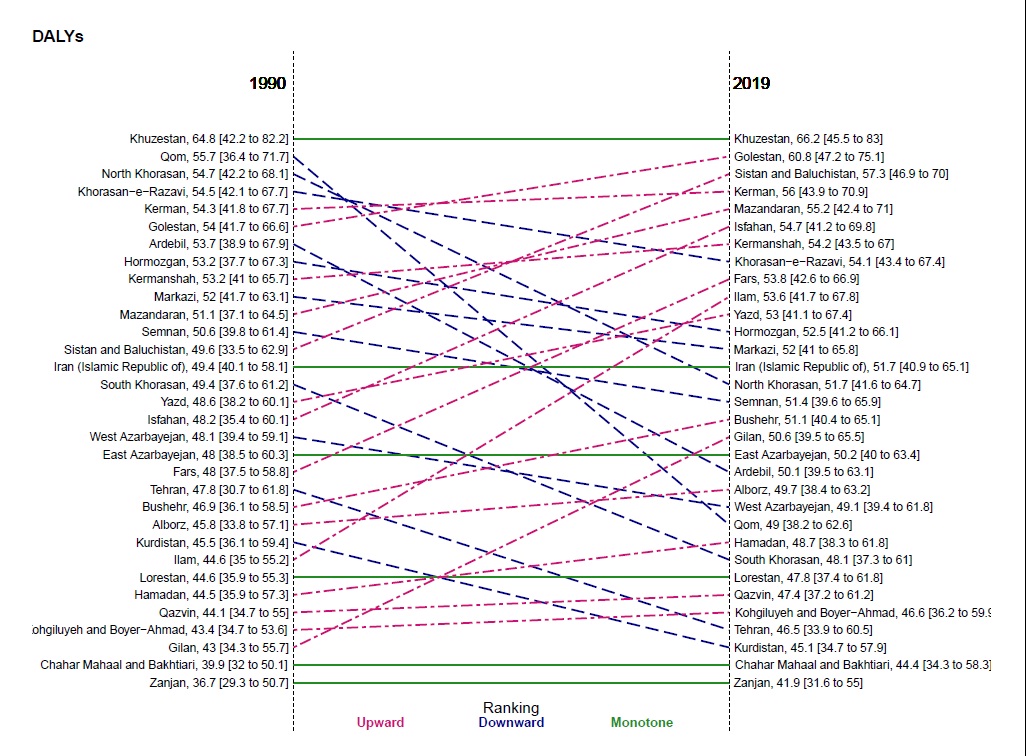

Supplement: Supplementary file 5 — Supplementary Information 4. [file 41598_2023_31096_MOESM5_ESM.jpg]
