# Supplementary material for: National and sub-national burden and trend of type 1 diabetes in 31 provinces of Iran, 1990–2019
Source: Sci Rep. 2023 Mar 14;13:4210. doi: 10.1038/s41598-023-31096-8 (PMC10014831; doi:10.1038/s41598-023-31096-8)
Supplement: Supplementary file 6 — Supplementary Information 6. [file 41598_2023_31096_MOESM6_ESM.docx]

| **Province name** | **Year** | **SDI** | **SDI quintiles** |
| --- | --- | --- | --- |
| Alborz | 1990 | 0.503 | 5 |
| Alborz | 1991 | 0.510 | 5 |
| Alborz | 1992 | 0.514 | 5 |
| Alborz | 1993 | 0.526 | 5 |
| Alborz | 1994 | 0.528 | 5 |
| Alborz | 1995 | 0.536 | 5 |
| Alborz | 1996 | 0.551 | 5 |
| Alborz | 1997 | 0.554 | 5 |
| Alborz | 1998 | 0.559 | 5 |
| Alborz | 1999 | 0.559 | 5 |
| Alborz | 2000 | 0.578 | 5 |
| Alborz | 2001 | 0.603 | 5 |
| Alborz | 2002 | 0.614 | 5 |
| Alborz | 2003 | 0.626 | 5 |
| Alborz | 2004 | 0.637 | 5 |
| Alborz | 2005 | 0.652 | 5 |
| Alborz | 2006 | 0.661 | 5 |
| Alborz | 2007 | 0.663 | 5 |
| Alborz | 2008 | 0.670 | 5 |
| Alborz | 2009 | 0.676 | 5 |
| Alborz | 2010 | 0.682 | 5 |
| Alborz | 2011 | 0.689 | 5 |
| Alborz | 2012 | 0.693 | 5 |
| Alborz | 2013 | 0.697 | 5 |
| Alborz | 2014 | 0.703 | 5 |
| Alborz | 2015 | 0.712 | 5 |
| Alborz | 2016 | 0.719 | 5 |
| Alborz | 2017 | 0.725 | 5 |
| Alborz | 2018 | 0.732 | 5 |
| Alborz | 2019 | 0.740 | 5 |

| **Province name** | **Year** | **SDI** | **SDI quintiles** |
| --- | --- | --- | --- |
| Ardebil | 1990 | 0.376 | 2 |
| Ardebil | 1991 | 0.384 | 2 |
| Ardebil | 1992 | 0.388 | 2 |
| Ardebil | 1993 | 0.402 | 2 |
| Ardebil | 1994 | 0.403 | 2 |
| Ardebil | 1995 | 0.413 | 2 |
| Ardebil | 1996 | 0.432 | 2 |
| Ardebil | 1997 | 0.438 | 2 |
| Ardebil | 1998 | 0.445 | 2 |
| Ardebil | 1999 | 0.446 | 2 |
| Ardebil | 2000 | 0.471 | 2 |
| Ardebil | 2001 | 0.501 | 2 |
| Ardebil | 2002 | 0.516 | 2 |
| Ardebil | 2003 | 0.531 | 2 |
| Ardebil | 2004 | 0.545 | 2 |
| Ardebil | 2005 | 0.563 | 2 |
| Ardebil | 2006 | 0.573 | 2 |
| Ardebil | 2007 | 0.577 | 2 |
| Ardebil | 2008 | 0.586 | 2 |
| Ardebil | 2009 | 0.593 | 2 |
| Ardebil | 2010 | 0.602 | 2 |
| Ardebil | 2011 | 0.609 | 2 |
| Ardebil | 2012 | 0.614 | 2 |
| Ardebil | 2013 | 0.620 | 2 |
| Ardebil | 2014 | 0.627 | 2 |
| Ardebil | 2015 | 0.637 | 2 |
| Ardebil | 2016 | 0.645 | 2 |
| Ardebil | 2017 | 0.650 | 2 |
| Ardebil | 2018 | 0.659 | 2 |
| Ardebil | 2019 | 0.667 | 2 |

| **Province name** | **Year** | **SDI** | **SDI quintiles** |
| --- | --- | --- | --- |
| Bushehr | 1990 | 0.407 | 3 |
| Bushehr | 1991 | 0.417 | 3 |
| Bushehr | 1992 | 0.421 | 3 |
| Bushehr | 1993 | 0.438 | 3 |
| Bushehr | 1994 | 0.430 | 3 |
| Bushehr | 1995 | 0.442 | 3 |
| Bushehr | 1996 | 0.471 | 4 |
| Bushehr | 1997 | 0.476 | 4 |
| Bushehr | 1998 | 0.479 | 3 |
| Bushehr | 1999 | 0.482 | 3 |
| Bushehr | 2000 | 0.514 | 4 |
| Bushehr | 2001 | 0.544 | 4 |
| Bushehr | 2002 | 0.558 | 4 |
| Bushehr | 2003 | 0.571 | 4 |
| Bushehr | 2004 | 0.585 | 4 |
| Bushehr | 2005 | 0.601 | 4 |
| Bushehr | 2006 | 0.610 | 4 |
| Bushehr | 2007 | 0.613 | 4 |
| Bushehr | 2008 | 0.620 | 4 |
| Bushehr | 2009 | 0.627 | 4 |
| Bushehr | 2010 | 0.634 | 4 |
| Bushehr | 2011 | 0.640 | 4 |
| Bushehr | 2012 | 0.644 | 4 |
| Bushehr | 2013 | 0.648 | 4 |
| Bushehr | 2014 | 0.656 | 4 |
| Bushehr | 2015 | 0.668 | 4 |
| Bushehr | 2016 | 0.678 | 4 |
| Bushehr | 2017 | 0.684 | 4 |
| Bushehr | 2018 | 0.693 | 4 |
| Bushehr | 2019 | 0.701 | 4 |

| **Province name** | **Year** | **SDI** | **SDI quintiles** |
| --- | --- | --- | --- |
| Chahar Mahaal and Bakhtiari | 1990 | 0.320 | 1 |
| Chahar Mahaal and Bakhtiari | 1991 | 0.316 | 1 |
| Chahar Mahaal and Bakhtiari | 1992 | 0.306 | 1 |
| Chahar Mahaal and Bakhtiari | 1993 | 0.322 | 1 |
| Chahar Mahaal and Bakhtiari | 1994 | 0.306 | 1 |
| Chahar Mahaal and Bakhtiari | 1995 | 0.306 | 1 |
| Chahar Mahaal and Bakhtiari | 1996 | 0.324 | 1 |
| Chahar Mahaal and Bakhtiari | 1997 | 0.302 | 1 |
| Chahar Mahaal and Bakhtiari | 1998 | 0.285 | 1 |
| Chahar Mahaal and Bakhtiari | 1999 | 0.154 | 1 |
| Chahar Mahaal and Bakhtiari | 2000 | 0.276 | 1 |
| Chahar Mahaal and Bakhtiari | 2001 | 0.389 | 1 |
| Chahar Mahaal and Bakhtiari | 2002 | 0.406 | 1 |
| Chahar Mahaal and Bakhtiari | 2003 | 0.425 | 1 |
| Chahar Mahaal and Bakhtiari | 2004 | 0.445 | 1 |
| Chahar Mahaal and Bakhtiari | 2005 | 0.486 | 1 |
| Chahar Mahaal and Bakhtiari | 2006 | 0.493 | 1 |
| Chahar Mahaal and Bakhtiari | 2007 | 0.467 | 1 |
| Chahar Mahaal and Bakhtiari | 2008 | 0.467 | 1 |
| Chahar Mahaal and Bakhtiari | 2009 | 0.467 | 1 |
| Chahar Mahaal and Bakhtiari | 2010 | 0.471 | 1 |
| Chahar Mahaal and Bakhtiari | 2011 | 0.473 | 1 |
| Chahar Mahaal and Bakhtiari | 2012 | 0.469 | 1 |
| Chahar Mahaal and Bakhtiari | 2013 | 0.469 | 1 |
| Chahar Mahaal and Bakhtiari | 2014 | 0.481 | 1 |
| Chahar Mahaal and Bakhtiari | 2015 | 0.516 | 1 |
| Chahar Mahaal and Bakhtiari | 2016 | 0.541 | 1 |
| Chahar Mahaal and Bakhtiari | 2017 | 0.549 | 1 |
| Chahar Mahaal and Bakhtiari | 2018 | 0.562 | 1 |
| Chahar Mahaal and Bakhtiari | 2019 | 0.572 | 1 |

| **Province name** | **Year** | **SDI** | **SDI quintiles** |
| --- | --- | --- | --- |
| East Azarbayejan | 1990 | 0.408 | 3 |
| East Azarbayejan | 1991 | 0.416 | 3 |
| East Azarbayejan | 1992 | 0.422 | 3 |
| East Azarbayejan | 1993 | 0.435 | 3 |
| East Azarbayejan | 1994 | 0.443 | 3 |
| East Azarbayejan | 1995 | 0.453 | 3 |
| East Azarbayejan | 1996 | 0.465 | 3 |
| East Azarbayejan | 1997 | 0.472 | 3 |
| East Azarbayejan | 1998 | 0.482 | 3 |
| East Azarbayejan | 1999 | 0.483 | 4 |
| East Azarbayejan | 2000 | 0.499 | 3 |
| East Azarbayejan | 2001 | 0.523 | 3 |
| East Azarbayejan | 2002 | 0.535 | 3 |
| East Azarbayejan | 2003 | 0.547 | 3 |
| East Azarbayejan | 2004 | 0.559 | 2 |
| East Azarbayejan | 2005 | 0.574 | 2 |
| East Azarbayejan | 2006 | 0.583 | 2 |
| East Azarbayejan | 2007 | 0.585 | 2 |
| East Azarbayejan | 2008 | 0.592 | 2 |
| East Azarbayejan | 2009 | 0.599 | 2 |
| East Azarbayejan | 2010 | 0.605 | 2 |
| East Azarbayejan | 2011 | 0.611 | 2 |
| East Azarbayejan | 2012 | 0.616 | 2 |
| East Azarbayejan | 2013 | 0.620 | 2 |
| East Azarbayejan | 2014 | 0.627 | 2 |
| East Azarbayejan | 2015 | 0.637 | 2 |
| East Azarbayejan | 2016 | 0.646 | 2 |
| East Azarbayejan | 2017 | 0.653 | 2 |
| East Azarbayejan | 2018 | 0.663 | 2 |
| East Azarbayejan | 2019 | 0.672 | 2 |

| **Province name** | **Year** | **SDI** | **SDI quintiles** |
| --- | --- | --- | --- |
| Fars | 1990 | 0.427 | 4 |
| Fars | 1991 | 0.436 | 4 |
| Fars | 1992 | 0.442 | 4 |
| Fars | 1993 | 0.458 | 4 |
| Fars | 1994 | 0.465 | 4 |
| Fars | 1995 | 0.477 | 4 |
| Fars | 1996 | 0.494 | 5 |
| Fars | 1997 | 0.502 | 5 |
| Fars | 1998 | 0.513 | 5 |
| Fars | 1999 | 0.515 | 5 |
| Fars | 2000 | 0.536 | 5 |
| Fars | 2001 | 0.564 | 5 |
| Fars | 2002 | 0.578 | 5 |
| Fars | 2003 | 0.591 | 5 |
| Fars | 2004 | 0.603 | 5 |
| Fars | 2005 | 0.619 | 5 |
| Fars | 2006 | 0.628 | 5 |
| Fars | 2007 | 0.631 | 5 |
| Fars | 2008 | 0.638 | 5 |
| Fars | 2009 | 0.644 | 5 |
| Fars | 2010 | 0.651 | 5 |
| Fars | 2011 | 0.658 | 5 |
| Fars | 2012 | 0.662 | 5 |
| Fars | 2013 | 0.666 | 5 |
| Fars | 2014 | 0.672 | 5 |
| Fars | 2015 | 0.680 | 5 |
| Fars | 2016 | 0.688 | 5 |
| Fars | 2017 | 0.693 | 5 |
| Fars | 2018 | 0.702 | 5 |
| Fars | 2019 | 0.709 | 5 |

| **Province name** | **Year** | **SDI** | **SDI quintiles** |
| --- | --- | --- | --- |
| Gilan | 1990 | 0.431 | 4 |
| Gilan | 1991 | 0.440 | 4 |
| Gilan | 1992 | 0.445 | 4 |
| Gilan | 1993 | 0.461 | 4 |
| Gilan | 1994 | 0.465 | 4 |
| Gilan | 1995 | 0.476 | 4 |
| Gilan | 1996 | 0.494 | 4 |
| Gilan | 1997 | 0.501 | 4 |
| Gilan | 1998 | 0.510 | 4 |
| Gilan | 1999 | 0.513 | 4 |
| Gilan | 2000 | 0.534 | 5 |
| Gilan | 2001 | 0.559 | 5 |
| Gilan | 2002 | 0.571 | 5 |
| Gilan | 2003 | 0.584 | 5 |
| Gilan | 2004 | 0.596 | 5 |
| Gilan | 2005 | 0.611 | 5 |
| Gilan | 2006 | 0.621 | 5 |
| Gilan | 2007 | 0.626 | 5 |
| Gilan | 2008 | 0.635 | 5 |
| Gilan | 2009 | 0.642 | 5 |
| Gilan | 2010 | 0.650 | 5 |
| Gilan | 2011 | 0.657 | 5 |
| Gilan | 2012 | 0.661 | 5 |
| Gilan | 2013 | 0.666 | 5 |
| Gilan | 2014 | 0.671 | 5 |
| Gilan | 2015 | 0.679 | 5 |
| Gilan | 2016 | 0.687 | 5 |
| Gilan | 2017 | 0.692 | 5 |
| Gilan | 2018 | 0.701 | 4 |
| Gilan | 2019 | 0.708 | 4 |

| **Province name** | **Year** | **SDI** | **SDI quintiles** |
| --- | --- | --- | --- |
| Golestan | 1990 | 0.437 | 5 |
| Golestan | 1991 | 0.445 | 5 |
| Golestan | 1992 | 0.451 | 5 |
| Golestan | 1993 | 0.462 | 5 |
| Golestan | 1994 | 0.469 | 5 |
| Golestan | 1995 | 0.479 | 5 |
| Golestan | 1996 | 0.491 | 4 |
| Golestan | 1997 | 0.498 | 4 |
| Golestan | 1998 | 0.506 | 4 |
| Golestan | 1999 | 0.509 | 4 |
| Golestan | 2000 | 0.524 | 4 |
| Golestan | 2001 | 0.544 | 3 |
| Golestan | 2002 | 0.554 | 3 |
| Golestan | 2003 | 0.566 | 3 |
| Golestan | 2004 | 0.577 | 3 |
| Golestan | 2005 | 0.592 | 3 |
| Golestan | 2006 | 0.601 | 3 |
| Golestan | 2007 | 0.606 | 4 |
| Golestan | 2008 | 0.614 | 4 |
| Golestan | 2009 | 0.621 | 4 |
| Golestan | 2010 | 0.629 | 4 |
| Golestan | 2011 | 0.636 | 3 |
| Golestan | 2012 | 0.640 | 4 |
| Golestan | 2013 | 0.644 | 4 |
| Golestan | 2014 | 0.650 | 4 |
| Golestan | 2015 | 0.658 | 3 |
| Golestan | 2016 | 0.666 | 3 |
| Golestan | 2017 | 0.671 | 3 |
| Golestan | 2018 | 0.679 | 3 |
| Golestan | 2019 | 0.686 | 3 |

| **Province name** | **Year** | **SDI** | **SDI quintiles** |
| --- | --- | --- | --- |
| Hamadan | 1990 | 0.397 | 2 |
| Hamadan | 1991 | 0.405 | 2 |
| Hamadan | 1992 | 0.411 | 2 |
| Hamadan | 1993 | 0.425 | 3 |
| Hamadan | 1994 | 0.430 | 3 |
| Hamadan | 1995 | 0.441 | 3 |
| Hamadan | 1996 | 0.458 | 3 |
| Hamadan | 1997 | 0.465 | 3 |
| Hamadan | 1998 | 0.474 | 3 |
| Hamadan | 1999 | 0.477 | 3 |
| Hamadan | 2000 | 0.497 | 3 |
| Hamadan | 2001 | 0.521 | 3 |
| Hamadan | 2002 | 0.534 | 3 |
| Hamadan | 2003 | 0.547 | 3 |
| Hamadan | 2004 | 0.560 | 3 |
| Hamadan | 2005 | 0.576 | 3 |
| Hamadan | 2006 | 0.586 | 3 |
| Hamadan | 2007 | 0.591 | 3 |
| Hamadan | 2008 | 0.600 | 3 |
| Hamadan | 2009 | 0.608 | 3 |
| Hamadan | 2010 | 0.615 | 3 |
| Hamadan | 2011 | 0.622 | 3 |
| Hamadan | 2012 | 0.626 | 3 |
| Hamadan | 2013 | 0.630 | 3 |
| Hamadan | 2014 | 0.636 | 3 |
| Hamadan | 2015 | 0.644 | 3 |
| Hamadan | 2016 | 0.651 | 2 |
| Hamadan | 2017 | 0.656 | 2 |
| Hamadan | 2018 | 0.665 | 2 |
| Hamadan | 2019 | 0.673 | 2 |

| **Province name** | **Year** | **SDI** | **SDI quintiles** |
| --- | --- | --- | --- |
| Hormozgan | 1990 | 0.379 | 2 |
| Hormozgan | 1991 | 0.388 | 2 |
| Hormozgan | 1992 | 0.393 | 2 |
| Hormozgan | 1993 | 0.407 | 2 |
| Hormozgan | 1994 | 0.405 | 2 |
| Hormozgan | 1995 | 0.416 | 2 |
| Hormozgan | 1996 | 0.438 | 2 |
| Hormozgan | 1997 | 0.443 | 2 |
| Hormozgan | 1998 | 0.448 | 2 |
| Hormozgan | 1999 | 0.450 | 2 |
| Hormozgan | 2000 | 0.478 | 2 |
| Hormozgan | 2001 | 0.509 | 2 |
| Hormozgan | 2002 | 0.522 | 2 |
| Hormozgan | 2003 | 0.537 | 2 |
| Hormozgan | 2004 | 0.551 | 2 |
| Hormozgan | 2005 | 0.570 | 2 |
| Hormozgan | 2006 | 0.579 | 2 |
| Hormozgan | 2007 | 0.581 | 2 |
| Hormozgan | 2008 | 0.588 | 2 |
| Hormozgan | 2009 | 0.594 | 2 |
| Hormozgan | 2010 | 0.600 | 2 |
| Hormozgan | 2011 | 0.606 | 2 |
| Hormozgan | 2012 | 0.609 | 2 |
| Hormozgan | 2013 | 0.613 | 2 |
| Hormozgan | 2014 | 0.621 | 2 |
| Hormozgan | 2015 | 0.634 | 2 |
| Hormozgan | 2016 | 0.645 | 2 |
| Hormozgan | 2017 | 0.653 | 2 |
| Hormozgan | 2018 | 0.662 | 2 |
| Hormozgan | 2019 | 0.671 | 2 |

| **Province name** | **Year** | **SDI** | **SDI quintiles** |
| --- | --- | --- | --- |
| Ilam | 1990 | 0.341 | 1 |
| Ilam | 1991 | 0.353 | 1 |
| Ilam | 1992 | 0.355 | 1 |
| Ilam | 1993 | 0.375 | 1 |
| Ilam | 1994 | 0.350 | 1 |
| Ilam | 1995 | 0.365 | 1 |
| Ilam | 1996 | 0.411 | 1 |
| Ilam | 1997 | 0.415 | 1 |
| Ilam | 1998 | 0.413 | 1 |
| Ilam | 1999 | 0.421 | 1 |
| Ilam | 2000 | 0.469 | 2 |
| Ilam | 2001 | 0.507 | 2 |
| Ilam | 2002 | 0.527 | 2 |
| Ilam | 2003 | 0.545 | 2 |
| Ilam | 2004 | 0.563 | 3 |
| Ilam | 2005 | 0.582 | 3 |
| Ilam | 2006 | 0.593 | 3 |
| Ilam | 2007 | 0.599 | 3 |
| Ilam | 2008 | 0.609 | 3 |
| Ilam | 2009 | 0.619 | 3 |
| Ilam | 2010 | 0.629 | 3 |
| Ilam | 2011 | 0.636 | 4 |
| Ilam | 2012 | 0.639 | 3 |
| Ilam | 2013 | 0.645 | 4 |
| Ilam | 2014 | 0.652 | 4 |
| Ilam | 2015 | 0.664 | 4 |
| Ilam | 2016 | 0.674 | 4 |
| Ilam | 2017 | 0.679 | 4 |
| Ilam | 2018 | 0.688 | 4 |
| Ilam | 2019 | 0.696 | 4 |

| **Province name** | **Year** | **SDI** | **SDI quintiles** |
| --- | --- | --- | --- |
| Isfahan | 1990 | 0.441 | 5 |
| Isfahan | 1991 | 0.450 | 5 |
| Isfahan | 1992 | 0.456 | 5 |
| Isfahan | 1993 | 0.472 | 5 |
| Isfahan | 1994 | 0.477 | 5 |
| Isfahan | 1995 | 0.488 | 5 |
| Isfahan | 1996 | 0.504 | 5 |
| Isfahan | 1997 | 0.511 | 5 |
| Isfahan | 1998 | 0.520 | 5 |
| Isfahan | 1999 | 0.521 | 5 |
| Isfahan | 2000 | 0.542 | 5 |
| Isfahan | 2001 | 0.568 | 5 |
| Isfahan | 2002 | 0.581 | 5 |
| Isfahan | 2003 | 0.593 | 5 |
| Isfahan | 2004 | 0.605 | 5 |
| Isfahan | 2005 | 0.620 | 5 |
| Isfahan | 2006 | 0.630 | 5 |
| Isfahan | 2007 | 0.635 | 5 |
| Isfahan | 2008 | 0.643 | 5 |
| Isfahan | 2009 | 0.651 | 5 |
| Isfahan | 2010 | 0.658 | 5 |
| Isfahan | 2011 | 0.665 | 5 |
| Isfahan | 2012 | 0.669 | 5 |
| Isfahan | 2013 | 0.673 | 5 |
| Isfahan | 2014 | 0.678 | 5 |
| Isfahan | 2015 | 0.685 | 5 |
| Isfahan | 2016 | 0.691 | 5 |
| Isfahan | 2017 | 0.695 | 5 |
| Isfahan | 2018 | 0.703 | 5 |
| Isfahan | 2019 | 0.710 | 5 |

| **Province name** | **Year** | **SDI** | **SDI quintiles** |
| --- | --- | --- | --- |
| Kerman | 1990 | 0.441 | 5 |
| Kerman | 1991 | 0.448 | 5 |
| Kerman | 1992 | 0.454 | 5 |
| Kerman | 1993 | 0.466 | 5 |
| Kerman | 1994 | 0.474 | 5 |
| Kerman | 1995 | 0.483 | 5 |
| Kerman | 1996 | 0.496 | 5 |
| Kerman | 1997 | 0.503 | 5 |
| Kerman | 1998 | 0.512 | 5 |
| Kerman | 1999 | 0.514 | 5 |
| Kerman | 2000 | 0.530 | 4 |
| Kerman | 2001 | 0.553 | 4 |
| Kerman | 2002 | 0.565 | 4 |
| Kerman | 2003 | 0.577 | 4 |
| Kerman | 2004 | 0.589 | 4 |
| Kerman | 2005 | 0.605 | 4 |
| Kerman | 2006 | 0.613 | 4 |
| Kerman | 2007 | 0.616 | 4 |
| Kerman | 2008 | 0.623 | 4 |
| Kerman | 2009 | 0.628 | 4 |
| Kerman | 2010 | 0.634 | 4 |
| Kerman | 2011 | 0.638 | 4 |
| Kerman | 2012 | 0.641 | 4 |
| Kerman | 2013 | 0.644 | 3 |
| Kerman | 2014 | 0.648 | 3 |
| Kerman | 2015 | 0.655 | 3 |
| Kerman | 2016 | 0.661 | 3 |
| Kerman | 2017 | 0.666 | 3 |
| Kerman | 2018 | 0.673 | 3 |
| Kerman | 2019 | 0.680 | 3 |

| **Province name** | **Year** | **SDI** | **SDI quintiles** |
| --- | --- | --- | --- |
| Kermanshah | 1990 | 0.398 | 3 |
| Kermanshah | 1991 | 0.406 | 3 |
| Kermanshah | 1992 | 0.411 | 3 |
| Kermanshah | 1993 | 0.425 | 2 |
| Kermanshah | 1994 | 0.429 | 3 |
| Kermanshah | 1995 | 0.440 | 3 |
| Kermanshah | 1996 | 0.455 | 2 |
| Kermanshah | 1997 | 0.462 | 2 |
| Kermanshah | 1998 | 0.470 | 3 |
| Kermanshah | 1999 | 0.471 | 2 |
| Kermanshah | 2000 | 0.490 | 2 |
| Kermanshah | 2001 | 0.514 | 2 |
| Kermanshah | 2002 | 0.527 | 2 |
| Kermanshah | 2003 | 0.540 | 2 |
| Kermanshah | 2004 | 0.554 | 2 |
| Kermanshah | 2005 | 0.571 | 2 |
| Kermanshah | 2006 | 0.582 | 2 |
| Kermanshah | 2007 | 0.587 | 2 |
| Kermanshah | 2008 | 0.597 | 2 |
| Kermanshah | 2009 | 0.605 | 3 |
| Kermanshah | 2010 | 0.614 | 3 |
| Kermanshah | 2011 | 0.621 | 3 |
| Kermanshah | 2012 | 0.626 | 3 |
| Kermanshah | 2013 | 0.631 | 3 |
| Kermanshah | 2014 | 0.637 | 3 |
| Kermanshah | 2015 | 0.646 | 3 |
| Kermanshah | 2016 | 0.654 | 3 |
| Kermanshah | 2017 | 0.659 | 3 |
| Kermanshah | 2018 | 0.667 | 2 |
| Kermanshah | 2019 | 0.675 | 2 |

| **Province name** | **Year** | **SDI** | **SDI quintiles** |
| --- | --- | --- | --- |
| Khorasan-e-Razavi | 1990 | 0.407 | 3 |
| Khorasan-e-Razavi | 1991 | 0.415 | 3 |
| Khorasan-e-Razavi | 1992 | 0.420 | 3 |
| Khorasan-e-Razavi | 1993 | 0.433 | 3 |
| Khorasan-e-Razavi | 1994 | 0.435 | 3 |
| Khorasan-e-Razavi | 1995 | 0.445 | 3 |
| Khorasan-e-Razavi | 1996 | 0.461 | 3 |
| Khorasan-e-Razavi | 1997 | 0.467 | 3 |
| Khorasan-e-Razavi | 1998 | 0.473 | 3 |
| Khorasan-e-Razavi | 1999 | 0.474 | 3 |
| Khorasan-e-Razavi | 2000 | 0.495 | 3 |
| Khorasan-e-Razavi | 2001 | 0.519 | 3 |
| Khorasan-e-Razavi | 2002 | 0.531 | 2 |
| Khorasan-e-Razavi | 2003 | 0.543 | 2 |
| Khorasan-e-Razavi | 2004 | 0.554 | 2 |
| Khorasan-e-Razavi | 2005 | 0.570 | 2 |
| Khorasan-e-Razavi | 2006 | 0.578 | 2 |
| Khorasan-e-Razavi | 2007 | 0.581 | 2 |
| Khorasan-e-Razavi | 2008 | 0.587 | 2 |
| Khorasan-e-Razavi | 2009 | 0.593 | 2 |
| Khorasan-e-Razavi | 2010 | 0.600 | 2 |
| Khorasan-e-Razavi | 2011 | 0.605 | 2 |
| Khorasan-e-Razavi | 2012 | 0.609 | 2 |
| Khorasan-e-Razavi | 2013 | 0.613 | 2 |
| Khorasan-e-Razavi | 2014 | 0.619 | 1 |
| Khorasan-e-Razavi | 2015 | 0.629 | 1 |
| Khorasan-e-Razavi | 2016 | 0.638 | 1 |
| Khorasan-e-Razavi | 2017 | 0.644 | 1 |
| Khorasan-e-Razavi | 2018 | 0.653 | 1 |
| Khorasan-e-Razavi | 2019 | 0.661 | 1 |

| **Province name** | **Year** | **SDI** | **SDI quintiles** |
| --- | --- | --- | --- |
| Khuzestan | 1990 | 0.411 | 4 |
| Khuzestan | 1991 | 0.420 | 4 |
| Khuzestan | 1992 | 0.426 | 4 |
| Khuzestan | 1993 | 0.440 | 4 |
| Khuzestan | 1994 | 0.445 | 4 |
| Khuzestan | 1995 | 0.455 | 4 |
| Khuzestan | 1996 | 0.470 | 3 |
| Khuzestan | 1997 | 0.475 | 3 |
| Khuzestan | 1998 | 0.483 | 4 |
| Khuzestan | 1999 | 0.482 | 3 |
| Khuzestan | 2000 | 0.502 | 3 |
| Khuzestan | 2001 | 0.530 | 3 |
| Khuzestan | 2002 | 0.543 | 3 |
| Khuzestan | 2003 | 0.556 | 3 |
| Khuzestan | 2004 | 0.568 | 3 |
| Khuzestan | 2005 | 0.585 | 3 |
| Khuzestan | 2006 | 0.594 | 3 |
| Khuzestan | 2007 | 0.596 | 3 |
| Khuzestan | 2008 | 0.603 | 3 |
| Khuzestan | 2009 | 0.609 | 3 |
| Khuzestan | 2010 | 0.616 | 3 |
| Khuzestan | 2011 | 0.622 | 3 |
| Khuzestan | 2012 | 0.626 | 3 |
| Khuzestan | 2013 | 0.630 | 3 |
| Khuzestan | 2014 | 0.637 | 3 |
| Khuzestan | 2015 | 0.648 | 3 |
| Khuzestan | 2016 | 0.657 | 3 |
| Khuzestan | 2017 | 0.663 | 3 |
| Khuzestan | 2018 | 0.672 | 3 |
| Khuzestan | 2019 | 0.680 | 3 |

| **Province name** | **Year** | **SDI** | **SDI quintiles** |
| --- | --- | --- | --- |
| Kohgiluyeh and Boyer-Ahmad | 1990 | 0.336 | 1 |
| Kohgiluyeh and Boyer-Ahmad | 1991 | 0.345 | 1 |
| Kohgiluyeh and Boyer-Ahmad | 1992 | 0.346 | 1 |
| Kohgiluyeh and Boyer-Ahmad | 1993 | 0.363 | 1 |
| Kohgiluyeh and Boyer-Ahmad | 1994 | 0.336 | 1 |
| Kohgiluyeh and Boyer-Ahmad | 1995 | 0.348 | 1 |
| Kohgiluyeh and Boyer-Ahmad | 1996 | 0.393 | 1 |
| Kohgiluyeh and Boyer-Ahmad | 1997 | 0.394 | 1 |
| Kohgiluyeh and Boyer-Ahmad | 1998 | 0.388 | 1 |
| Kohgiluyeh and Boyer-Ahmad | 1999 | 0.391 | 1 |
| Kohgiluyeh and Boyer-Ahmad | 2000 | 0.440 | 1 |
| Kohgiluyeh and Boyer-Ahmad | 2001 | 0.479 | 1 |
| Kohgiluyeh and Boyer-Ahmad | 2002 | 0.497 | 1 |
| Kohgiluyeh and Boyer-Ahmad | 2003 | 0.514 | 1 |
| Kohgiluyeh and Boyer-Ahmad | 2004 | 0.531 | 1 |
| Kohgiluyeh and Boyer-Ahmad | 2005 | 0.551 | 1 |
| Kohgiluyeh and Boyer-Ahmad | 2006 | 0.561 | 1 |
| Kohgiluyeh and Boyer-Ahmad | 2007 | 0.564 | 1 |
| Kohgiluyeh and Boyer-Ahmad | 2008 | 0.572 | 1 |
| Kohgiluyeh and Boyer-Ahmad | 2009 | 0.579 | 1 |
| Kohgiluyeh and Boyer-Ahmad | 2010 | 0.589 | 1 |
| Kohgiluyeh and Boyer-Ahmad | 2011 | 0.597 | 1 |
| Kohgiluyeh and Boyer-Ahmad | 2012 | 0.601 | 1 |
| Kohgiluyeh and Boyer-Ahmad | 2013 | 0.609 | 1 |
| Kohgiluyeh and Boyer-Ahmad | 2014 | 0.620 | 2 |
| Kohgiluyeh and Boyer-Ahmad | 2015 | 0.638 | 2 |
| Kohgiluyeh and Boyer-Ahmad | 2016 | 0.651 | 2 |
| Kohgiluyeh and Boyer-Ahmad | 2017 | 0.659 | 2 |
| Kohgiluyeh and Boyer-Ahmad | 2018 | 0.669 | 3 |
| Kohgiluyeh and Boyer-Ahmad | 2019 | 0.677 | 3 |

| **Province name** | **Year** | **SDI** | **SDI quintiles** |
| --- | --- | --- | --- |
| Kurdistan | 1990 | 0.358 | 1 |
| Kurdistan | 1991 | 0.367 | 1 |
| Kurdistan | 1992 | 0.373 | 1 |
| Kurdistan | 1993 | 0.385 | 1 |
| Kurdistan | 1994 | 0.391 | 1 |
| Kurdistan | 1995 | 0.401 | 1 |
| Kurdistan | 1996 | 0.415 | 1 |
| Kurdistan | 1997 | 0.422 | 1 |
| Kurdistan | 1998 | 0.430 | 1 |
| Kurdistan | 1999 | 0.434 | 1 |
| Kurdistan | 2000 | 0.452 | 1 |
| Kurdistan | 2001 | 0.474 | 1 |
| Kurdistan | 2002 | 0.487 | 1 |
| Kurdistan | 2003 | 0.501 | 1 |
| Kurdistan | 2004 | 0.515 | 1 |
| Kurdistan | 2005 | 0.532 | 1 |
| Kurdistan | 2006 | 0.544 | 1 |
| Kurdistan | 2007 | 0.551 | 1 |
| Kurdistan | 2008 | 0.560 | 1 |
| Kurdistan | 2009 | 0.569 | 1 |
| Kurdistan | 2010 | 0.578 | 1 |
| Kurdistan | 2011 | 0.586 | 1 |
| Kurdistan | 2012 | 0.592 | 1 |
| Kurdistan | 2013 | 0.598 | 1 |
| Kurdistan | 2014 | 0.605 | 1 |
| Kurdistan | 2015 | 0.614 | 1 |
| Kurdistan | 2016 | 0.622 | 1 |
| Kurdistan | 2017 | 0.628 | 1 |
| Kurdistan | 2018 | 0.636 | 1 |
| Kurdistan | 2019 | 0.645 | 1 |

| **Province name** | **Year** | **SDI** | **SDI quintiles** |
| --- | --- | --- | --- |
| Lorestan | 1990 | 0.403 | 3 |
| Lorestan | 1991 | 0.412 | 3 |
| Lorestan | 1992 | 0.418 | 3 |
| Lorestan | 1993 | 0.431 | 3 |
| Lorestan | 1994 | 0.437 | 3 |
| Lorestan | 1995 | 0.447 | 3 |
| Lorestan | 1996 | 0.463 | 3 |
| Lorestan | 1997 | 0.470 | 3 |
| Lorestan | 1998 | 0.478 | 3 |
| Lorestan | 1999 | 0.481 | 3 |
| Lorestan | 2000 | 0.500 | 3 |
| Lorestan | 2001 | 0.522 | 3 |
| Lorestan | 2002 | 0.535 | 3 |
| Lorestan | 2003 | 0.549 | 3 |
| Lorestan | 2004 | 0.562 | 3 |
| Lorestan | 2005 | 0.579 | 3 |
| Lorestan | 2006 | 0.589 | 3 |
| Lorestan | 2007 | 0.595 | 3 |
| Lorestan | 2008 | 0.603 | 3 |
| Lorestan | 2009 | 0.611 | 3 |
| Lorestan | 2010 | 0.619 | 3 |
| Lorestan | 2011 | 0.626 | 3 |
| Lorestan | 2012 | 0.631 | 3 |
| Lorestan | 2013 | 0.636 | 3 |
| Lorestan | 2014 | 0.642 | 3 |
| Lorestan | 2015 | 0.651 | 3 |
| Lorestan | 2016 | 0.659 | 3 |
| Lorestan | 2017 | 0.664 | 3 |
| Lorestan | 2018 | 0.673 | 3 |
| Lorestan | 2019 | 0.681 | 3 |

| **Province name** | **Year** | **SDI** | **SDI quintiles** |
| --- | --- | --- | --- |
| Markazi | 1990 | 0.428 | 4 |
| Markazi | 1991 | 0.436 | 4 |
| Markazi | 1992 | 0.443 | 4 |
| Markazi | 1993 | 0.455 | 4 |
| Markazi | 1994 | 0.461 | 4 |
| Markazi | 1995 | 0.471 | 4 |
| Markazi | 1996 | 0.486 | 4 |
| Markazi | 1997 | 0.494 | 4 |
| Markazi | 1998 | 0.503 | 4 |
| Markazi | 1999 | 0.507 | 4 |
| Markazi | 2000 | 0.525 | 4 |
| Markazi | 2001 | 0.545 | 4 |
| Markazi | 2002 | 0.557 | 4 |
| Markazi | 2003 | 0.569 | 4 |
| Markazi | 2004 | 0.581 | 4 |
| Markazi | 2005 | 0.595 | 4 |
| Markazi | 2006 | 0.605 | 4 |
| Markazi | 2007 | 0.610 | 4 |
| Markazi | 2008 | 0.617 | 4 |
| Markazi | 2009 | 0.624 | 4 |
| Markazi | 2010 | 0.632 | 4 |
| Markazi | 2011 | 0.638 | 4 |
| Markazi | 2012 | 0.642 | 4 |
| Markazi | 2013 | 0.646 | 4 |
| Markazi | 2014 | 0.652 | 4 |
| Markazi | 2015 | 0.659 | 4 |
| Markazi | 2016 | 0.666 | 4 |
| Markazi | 2017 | 0.671 | 4 |
| Markazi | 2018 | 0.679 | 4 |
| Markazi | 2019 | 0.686 | 4 |

| **Province name** | **Year** | **SDI** | **SDI quintiles** |
| --- | --- | --- | --- |
| Mazandaran | 1990 | 0.438 | 5 |
| Mazandaran | 1991 | 0.447 | 5 |
| Mazandaran | 1992 | 0.453 | 5 |
| Mazandaran | 1993 | 0.469 | 5 |
| Mazandaran | 1994 | 0.474 | 5 |
| Mazandaran | 1995 | 0.486 | 5 |
| Mazandaran | 1996 | 0.502 | 5 |
| Mazandaran | 1997 | 0.509 | 5 |
| Mazandaran | 1998 | 0.519 | 5 |
| Mazandaran | 1999 | 0.522 | 5 |
| Mazandaran | 2000 | 0.543 | 5 |
| Mazandaran | 2001 | 0.570 | 5 |
| Mazandaran | 2002 | 0.584 | 5 |
| Mazandaran | 2003 | 0.599 | 5 |
| Mazandaran | 2004 | 0.614 | 5 |
| Mazandaran | 2005 | 0.632 | 5 |
| Mazandaran | 2006 | 0.643 | 5 |
| Mazandaran | 2007 | 0.650 | 5 |
| Mazandaran | 2008 | 0.659 | 5 |
| Mazandaran | 2009 | 0.667 | 5 |
| Mazandaran | 2010 | 0.674 | 5 |
| Mazandaran | 2011 | 0.681 | 5 |
| Mazandaran | 2012 | 0.685 | 5 |
| Mazandaran | 2013 | 0.689 | 5 |
| Mazandaran | 2014 | 0.694 | 5 |
| Mazandaran | 2015 | 0.701 | 5 |
| Mazandaran | 2016 | 0.708 | 5 |
| Mazandaran | 2017 | 0.713 | 5 |
| Mazandaran | 2018 | 0.721 | 5 |
| Mazandaran | 2019 | 0.729 | 5 |

| **Province name** | **Year** | **SDI** | **SDI quintiles** |
| --- | --- | --- | --- |
| North Khorasan | 1990 | 0.375 | 2 |
| North Khorasan | 1991 | 0.383 | 2 |
| North Khorasan | 1992 | 0.388 | 2 |
| North Khorasan | 1993 | 0.400 | 2 |
| North Khorasan | 1994 | 0.403 | 1 |
| North Khorasan | 1995 | 0.412 | 1 |
| North Khorasan | 1996 | 0.429 | 2 |
| North Khorasan | 1997 | 0.435 | 2 |
| North Khorasan | 1998 | 0.441 | 2 |
| North Khorasan | 1999 | 0.443 | 1 |
| North Khorasan | 2000 | 0.465 | 1 |
| North Khorasan | 2001 | 0.492 | 1 |
| North Khorasan | 2002 | 0.505 | 1 |
| North Khorasan | 2003 | 0.519 | 1 |
| North Khorasan | 2004 | 0.533 | 1 |
| North Khorasan | 2005 | 0.551 | 1 |
| North Khorasan | 2006 | 0.561 | 1 |
| North Khorasan | 2007 | 0.566 | 1 |
| North Khorasan | 2008 | 0.575 | 1 |
| North Khorasan | 2009 | 0.583 | 1 |
| North Khorasan | 2010 | 0.592 | 1 |
| North Khorasan | 2011 | 0.600 | 1 |
| North Khorasan | 2012 | 0.605 | 1 |
| North Khorasan | 2013 | 0.611 | 1 |
| North Khorasan | 2014 | 0.618 | 1 |
| North Khorasan | 2015 | 0.629 | 1 |
| North Khorasan | 2016 | 0.639 | 1 |
| North Khorasan | 2017 | 0.646 | 1 |
| North Khorasan | 2018 | 0.655 | 1 |
| North Khorasan | 2019 | 0.664 | 1 |

| **Province name** | **Year** | **SDI** | **SDI quintiles** |
| --- | --- | --- | --- |
| Qazvin | 1990 | 0.396 | 2 |
| Qazvin | 1991 | 0.404 | 2 |
| Qazvin | 1992 | 0.408 | 2 |
| Qazvin | 1993 | 0.423 | 2 |
| Qazvin | 1994 | 0.423 | 2 |
| Qazvin | 1995 | 0.433 | 2 |
| Qazvin | 1996 | 0.452 | 2 |
| Qazvin | 1997 | 0.456 | 2 |
| Qazvin | 1998 | 0.461 | 2 |
| Qazvin | 1999 | 0.458 | 2 |
| Qazvin | 2000 | 0.485 | 2 |
| Qazvin | 2001 | 0.518 | 2 |
| Qazvin | 2002 | 0.532 | 3 |
| Qazvin | 2003 | 0.546 | 3 |
| Qazvin | 2004 | 0.560 | 3 |
| Qazvin | 2005 | 0.579 | 3 |
| Qazvin | 2006 | 0.589 | 3 |
| Qazvin | 2007 | 0.590 | 3 |
| Qazvin | 2008 | 0.597 | 3 |
| Qazvin | 2009 | 0.603 | 2 |
| Qazvin | 2010 | 0.611 | 2 |
| Qazvin | 2011 | 0.617 | 2 |
| Qazvin | 2012 | 0.620 | 2 |
| Qazvin | 2013 | 0.625 | 2 |
| Qazvin | 2014 | 0.632 | 2 |
| Qazvin | 2015 | 0.644 | 2 |
| Qazvin | 2016 | 0.654 | 3 |
| Qazvin | 2017 | 0.660 | 3 |
| Qazvin | 2018 | 0.670 | 3 |
| Qazvin | 2019 | 0.678 | 3 |

| **Province name** | **Year** | **SDI** | **SDI quintiles** |
| --- | --- | --- | --- |
| Qom | 1990 | 0.430 | 4 |
| Qom | 1991 | 0.438 | 4 |
| Qom | 1992 | 0.443 | 4 |
| Qom | 1993 | 0.457 | 4 |
| Qom | 1994 | 0.457 | 4 |
| Qom | 1995 | 0.467 | 4 |
| Qom | 1996 | 0.486 | 4 |
| Qom | 1997 | 0.492 | 4 |
| Qom | 1998 | 0.497 | 4 |
| Qom | 1999 | 0.500 | 4 |
| Qom | 2000 | 0.522 | 4 |
| Qom | 2001 | 0.545 | 4 |
| Qom | 2002 | 0.557 | 4 |
| Qom | 2003 | 0.568 | 4 |
| Qom | 2004 | 0.579 | 4 |
| Qom | 2005 | 0.594 | 4 |
| Qom | 2006 | 0.602 | 4 |
| Qom | 2007 | 0.605 | 3 |
| Qom | 2008 | 0.612 | 3 |
| Qom | 2009 | 0.619 | 3 |
| Qom | 2010 | 0.626 | 3 |
| Qom | 2011 | 0.632 | 3 |
| Qom | 2012 | 0.636 | 3 |
| Qom | 2013 | 0.641 | 3 |
| Qom | 2014 | 0.649 | 3 |
| Qom | 2015 | 0.659 | 4 |
| Qom | 2016 | 0.669 | 4 |
| Qom | 2017 | 0.675 | 4 |
| Qom | 2018 | 0.684 | 4 |
| Qom | 2019 | 0.692 | 4 |

| **Province name** | **Year** | **SDI** | **SDI quintiles** |
| --- | --- | --- | --- |
| Semnan | 1990 | 0.398 | 3 |
| Semnan | 1991 | 0.409 | 3 |
| Semnan | 1992 | 0.412 | 3 |
| Semnan | 1993 | 0.430 | 3 |
| Semnan | 1994 | 0.412 | 2 |
| Semnan | 1995 | 0.425 | 2 |
| Semnan | 1996 | 0.463 | 3 |
| Semnan | 1997 | 0.467 | 3 |
| Semnan | 1998 | 0.469 | 2 |
| Semnan | 1999 | 0.475 | 3 |
| Semnan | 2000 | 0.513 | 3 |
| Semnan | 2001 | 0.545 | 4 |
| Semnan | 2002 | 0.560 | 4 |
| Semnan | 2003 | 0.574 | 4 |
| Semnan | 2004 | 0.588 | 4 |
| Semnan | 2005 | 0.604 | 4 |
| Semnan | 2006 | 0.612 | 4 |
| Semnan | 2007 | 0.616 | 4 |
| Semnan | 2008 | 0.623 | 4 |
| Semnan | 2009 | 0.630 | 4 |
| Semnan | 2010 | 0.638 | 4 |
| Semnan | 2011 | 0.644 | 4 |
| Semnan | 2012 | 0.647 | 4 |
| Semnan | 2013 | 0.653 | 4 |
| Semnan | 2014 | 0.660 | 4 |
| Semnan | 2015 | 0.673 | 4 |
| Semnan | 2016 | 0.683 | 4 |
| Semnan | 2017 | 0.689 | 4 |
| Semnan | 2018 | 0.697 | 4 |
| Semnan | 2019 | 0.704 | 4 |

| **Province name** | **Year** | **SDI** | **SDI quintiles** |
| --- | --- | --- | --- |
| Sistan and Baluchistan | 1990 | 0.346 | 1 |
| Sistan and Baluchistan | 1991 | 0.353 | 1 |
| Sistan and Baluchistan | 1992 | 0.358 | 1 |
| Sistan and Baluchistan | 1993 | 0.370 | 1 |
| Sistan and Baluchistan | 1994 | 0.371 | 1 |
| Sistan and Baluchistan | 1995 | 0.379 | 1 |
| Sistan and Baluchistan | 1996 | 0.394 | 1 |
| Sistan and Baluchistan | 1997 | 0.396 | 1 |
| Sistan and Baluchistan | 1998 | 0.398 | 1 |
| Sistan and Baluchistan | 1999 | 0.392 | 1 |
| Sistan and Baluchistan | 2000 | 0.414 | 1 |
| Sistan and Baluchistan | 2001 | 0.442 | 1 |
| Sistan and Baluchistan | 2002 | 0.452 | 1 |
| Sistan and Baluchistan | 2003 | 0.462 | 1 |
| Sistan and Baluchistan | 2004 | 0.474 | 1 |
| Sistan and Baluchistan | 2005 | 0.492 | 1 |
| Sistan and Baluchistan | 2006 | 0.499 | 1 |
| Sistan and Baluchistan | 2007 | 0.497 | 1 |
| Sistan and Baluchistan | 2008 | 0.503 | 1 |
| Sistan and Baluchistan | 2009 | 0.508 | 1 |
| Sistan and Baluchistan | 2010 | 0.516 | 1 |
| Sistan and Baluchistan | 2011 | 0.522 | 1 |
| Sistan and Baluchistan | 2012 | 0.526 | 1 |
| Sistan and Baluchistan | 2013 | 0.531 | 1 |
| Sistan and Baluchistan | 2014 | 0.540 | 1 |
| Sistan and Baluchistan | 2015 | 0.555 | 1 |
| Sistan and Baluchistan | 2016 | 0.568 | 1 |
| Sistan and Baluchistan | 2017 | 0.575 | 1 |
| Sistan and Baluchistan | 2018 | 0.584 | 1 |
| Sistan and Baluchistan | 2019 | 0.592 | 1 |

| **Province name** | **Year** | **SDI** | **SDI quintiles** |
| --- | --- | --- | --- |
| South Khorasan | 1990 | 0.370 | 1 |
| South Khorasan | 1991 | 0.378 | 1 |
| South Khorasan | 1992 | 0.383 | 1 |
| South Khorasan | 1993 | 0.396 | 1 |
| South Khorasan | 1994 | 0.399 | 1 |
| South Khorasan | 1995 | 0.409 | 1 |
| South Khorasan | 1996 | 0.426 | 1 |
| South Khorasan | 1997 | 0.433 | 1 |
| South Khorasan | 1998 | 0.440 | 1 |
| South Khorasan | 1999 | 0.443 | 2 |
| South Khorasan | 2000 | 0.464 | 1 |
| South Khorasan | 2001 | 0.489 | 1 |
| South Khorasan | 2002 | 0.502 | 1 |
| South Khorasan | 2003 | 0.516 | 1 |
| South Khorasan | 2004 | 0.530 | 1 |
| South Khorasan | 2005 | 0.546 | 1 |
| South Khorasan | 2006 | 0.557 | 1 |
| South Khorasan | 2007 | 0.561 | 1 |
| South Khorasan | 2008 | 0.569 | 1 |
| South Khorasan | 2009 | 0.577 | 1 |
| South Khorasan | 2010 | 0.585 | 1 |
| South Khorasan | 2011 | 0.593 | 1 |
| South Khorasan | 2012 | 0.598 | 1 |
| South Khorasan | 2013 | 0.604 | 1 |
| South Khorasan | 2014 | 0.612 | 1 |
| South Khorasan | 2015 | 0.624 | 1 |
| South Khorasan | 2016 | 0.635 | 1 |
| South Khorasan | 2017 | 0.642 | 1 |
| South Khorasan | 2018 | 0.652 | 1 |
| South Khorasan | 2019 | 0.660 | 1 |

| **Province name** | **Year** | **SDI** | **SDI quintiles** |
| --- | --- | --- | --- |
| Tehran | 1990 | 0.503 | 5 |
| Tehran | 1991 | 0.512 | 5 |
| Tehran | 1992 | 0.517 | 5 |
| Tehran | 1993 | 0.533 | 5 |
| Tehran | 1994 | 0.537 | 5 |
| Tehran | 1995 | 0.547 | 5 |
| Tehran | 1996 | 0.564 | 5 |
| Tehran | 1997 | 0.569 | 5 |
| Tehran | 1998 | 0.577 | 5 |
| Tehran | 1999 | 0.576 | 5 |
| Tehran | 2000 | 0.597 | 5 |
| Tehran | 2001 | 0.626 | 5 |
| Tehran | 2002 | 0.638 | 5 |
| Tehran | 2003 | 0.650 | 5 |
| Tehran | 2004 | 0.661 | 5 |
| Tehran | 2005 | 0.677 | 5 |
| Tehran | 2006 | 0.685 | 5 |
| Tehran | 2007 | 0.687 | 5 |
| Tehran | 2008 | 0.694 | 5 |
| Tehran | 2009 | 0.700 | 5 |
| Tehran | 2010 | 0.707 | 5 |
| Tehran | 2011 | 0.713 | 5 |
| Tehran | 2012 | 0.717 | 5 |
| Tehran | 2013 | 0.721 | 5 |
| Tehran | 2014 | 0.727 | 5 |
| Tehran | 2015 | 0.736 | 5 |
| Tehran | 2016 | 0.744 | 5 |
| Tehran | 2017 | 0.749 | 5 |
| Tehran | 2018 | 0.757 | 5 |
| Tehran | 2019 | 0.764 | 5 |

| **Province name** | **Year** | **SDI** | **SDI quintiles** |
| --- | --- | --- | --- |
| West Azarbayejan | 1990 | 0.375 | 1 |
| West Azarbayejan | 1991 | 0.382 | 1 |
| West Azarbayejan | 1992 | 0.387 | 1 |
| West Azarbayejan | 1993 | 0.399 | 1 |
| West Azarbayejan | 1994 | 0.403 | 2 |
| West Azarbayejan | 1995 | 0.413 | 2 |
| West Azarbayejan | 1996 | 0.428 | 1 |
| West Azarbayejan | 1997 | 0.433 | 1 |
| West Azarbayejan | 1998 | 0.440 | 1 |
| West Azarbayejan | 1999 | 0.440 | 1 |
| West Azarbayejan | 2000 | 0.460 | 1 |
| West Azarbayejan | 2001 | 0.486 | 1 |
| West Azarbayejan | 2002 | 0.498 | 1 |
| West Azarbayejan | 2003 | 0.511 | 1 |
| West Azarbayejan | 2004 | 0.524 | 1 |
| West Azarbayejan | 2005 | 0.541 | 1 |
| West Azarbayejan | 2006 | 0.551 | 1 |
| West Azarbayejan | 2007 | 0.554 | 1 |
| West Azarbayejan | 2008 | 0.561 | 1 |
| West Azarbayejan | 2009 | 0.567 | 1 |
| West Azarbayejan | 2010 | 0.573 | 1 |
| West Azarbayejan | 2011 | 0.579 | 1 |
| West Azarbayejan | 2012 | 0.581 | 1 |
| West Azarbayejan | 2013 | 0.585 | 1 |
| West Azarbayejan | 2014 | 0.590 | 1 |
| West Azarbayejan | 2015 | 0.600 | 1 |
| West Azarbayejan | 2016 | 0.609 | 1 |
| West Azarbayejan | 2017 | 0.614 | 1 |
| West Azarbayejan | 2018 | 0.623 | 1 |
| West Azarbayejan | 2019 | 0.631 | 1 |

| **Province name** | **Year** | **SDI** | **SDI quintiles** |
| --- | --- | --- | --- |
| Yazd | 1990 | 0.434 | 4 |
| Yazd | 1991 | 0.441 | 4 |
| Yazd | 1992 | 0.445 | 4 |
| Yazd | 1993 | 0.459 | 4 |
| Yazd | 1994 | 0.456 | 4 |
| Yazd | 1995 | 0.467 | 4 |
| Yazd | 1996 | 0.489 | 4 |
| Yazd | 1997 | 0.494 | 4 |
| Yazd | 1998 | 0.499 | 4 |
| Yazd | 1999 | 0.502 | 4 |
| Yazd | 2000 | 0.528 | 4 |
| Yazd | 2001 | 0.556 | 4 |
| Yazd | 2002 | 0.569 | 4 |
| Yazd | 2003 | 0.581 | 4 |
| Yazd | 2004 | 0.594 | 4 |
| Yazd | 2005 | 0.610 | 4 |
| Yazd | 2006 | 0.618 | 4 |
| Yazd | 2007 | 0.621 | 4 |
| Yazd | 2008 | 0.628 | 4 |
| Yazd | 2009 | 0.634 | 4 |
| Yazd | 2010 | 0.641 | 4 |
| Yazd | 2011 | 0.647 | 4 |
| Yazd | 2012 | 0.650 | 4 |
| Yazd | 2013 | 0.655 | 4 |
| Yazd | 2014 | 0.662 | 4 |
| Yazd | 2015 | 0.674 | 4 |
| Yazd | 2016 | 0.685 | 4 |
| Yazd | 2017 | 0.692 | 4 |
| Yazd | 2018 | 0.702 | 5 |
| Yazd | 2019 | 0.710 | 5 |

| **Province name** | **Year** | **SDI** | **SDI quintiles** |
| --- | --- | --- | --- |
| Zanjan | 1990 | 0.384 | 2 |
| Zanjan | 1991 | 0.392 | 2 |
| Zanjan | 1992 | 0.397 | 2 |
| Zanjan | 1993 | 0.409 | 2 |
| Zanjan | 1994 | 0.410 | 2 |
| Zanjan | 1995 | 0.419 | 2 |
| Zanjan | 1996 | 0.437 | 2 |
| Zanjan | 1997 | 0.443 | 2 |
| Zanjan | 1998 | 0.449 | 2 |
| Zanjan | 1999 | 0.452 | 2 |
| Zanjan | 2000 | 0.474 | 2 |
| Zanjan | 2001 | 0.498 | 2 |
| Zanjan | 2002 | 0.512 | 2 |
| Zanjan | 2003 | 0.526 | 2 |
| Zanjan | 2004 | 0.540 | 2 |
| Zanjan | 2005 | 0.557 | 2 |
| Zanjan | 2006 | 0.569 | 2 |
| Zanjan | 2007 | 0.575 | 2 |
| Zanjan | 2008 | 0.584 | 2 |
| Zanjan | 2009 | 0.592 | 2 |
| Zanjan | 2010 | 0.600 | 2 |
| Zanjan | 2011 | 0.607 | 2 |
| Zanjan | 2012 | 0.611 | 2 |
| Zanjan | 2013 | 0.615 | 2 |
| Zanjan | 2014 | 0.622 | 2 |
| Zanjan | 2015 | 0.631 | 2 |
| Zanjan | 2016 | 0.640 | 2 |
| Zanjan | 2017 | 0.646 | 2 |
| Zanjan | 2018 | 0.655 | 2 |
| Zanjan | 2019 | 0.664 | 2 |
