# Supplementary material for: National and sub-national burden and trend of type 1 diabetes in 31 provinces of Iran, 1990–2019
Source: Sci Rep. 2023 Mar 14;13:4210. doi: 10.1038/s41598-023-31096-8 (PMC10014831; doi:10.1038/s41598-023-31096-8)
Supplement: Supplementary file 7 — Supplementary Information 7. [file 41598_2023_31096_MOESM7_ESM.docx]

| **Province** | **Measure** | **Age-standardized rate (per 100,000)** | | | | | | **% Change (1990 to 2019)** | | |
| --- | --- | --- | --- | --- | --- | --- | --- | --- | --- | --- |
|  |  | **1990** | | | **2019** | | |  |  |  |
|  |  | **Both** | **Female** | **Male** | **Both** | **Female** | **Male** | **Both** | **Female** | **Male** |
| **Alborz** | **Incidence** | 6.14 (4.94 to 7.56) | 5.99 (4.81 to 7.37) | 6.28 (5.06 to 7.65) | 11.51 (9.28 to 14.05) | 11.31 (9.12 to 13.84) | 11.71 (9.39 to 14.31) | 87.3 (80.1 to 94.7) | 88.7 (78.2 to 99.8) | 86.4 (77.1 to 97) |
|  | **Prevalence** | 209.98 (165.85 to 263.15) | 204.47 (161.34 to 255.31) | 214.8 (169.76 to 267.51) | 404.14 (320.61 to 500.76) | 394.86 (311.11 to 489.18) | 413.23 (323.98 to 510.13) | 92.5 (84 to 101.2) | 93.1 (81.2 to 106) | 92.4 (81.5 to 104.7) |
|  | **Deaths** | 0.92 (0.63 to 1.19) | 1.02 (0.6 to 1.45) | 0.83 (0.55 to 1.12) | 0.63 (0.51 to 0.76) | 0.65 (0.45 to 0.86) | 0.63 (0.47 to 0.82) | -31.8 (-50.8 to 1.9) | -35.7 (-58.6 to 13.2) | -24.4 (-50 to 20.5) |
|  | **DALYs** | 45.83 (33.76 to 57.14) | 48.06 (32.71 to 63.7) | 43.63 (32.34 to 55.53) | 49.72 (38.41 to 63.22) | 48.23 (36.3 to 63.23) | 51.32 (39.38 to 65.6) | 8.5 (-13.6 to 43) | 0.4 (-27 to 41.9) | 17.6 (-9 to 52) |
|  | **YLLs** | 30.2 (20.35 to 39.06) | 32.68 (18.31 to 47.22) | 27.79 (18.65 to 36.71) | 19.99 (16.08 to 23.63) | 18.91 (11.62 to 24.22) | 21.19 (16.54 to 26.69) | -33.8 (-51.2 to 3.4) | -42.1 (-61.3 to -2.3) | -23.7 (-49.1 to 18.3) |
|  | **YLDs** | 15.62 (10.18 to 22.84) | 15.38 (9.84 to 22.79) | 15.84 (10.3 to 23.11) | 29.73 (19.32 to 43.2) | 29.32 (18.9 to 42.6) | 30.13 (19.53 to 43.76) | 90.3 (77.1 to 105.2) | 90.7 (70.3 to 113.4) | 90.2 (71.2 to 111.5) |
| **Ardebil** | **Incidence** | 5.55 (4.45 to 6.9) | 5.41 (4.32 to 6.71) | 5.7 (4.54 to 7.1) | 10.7 (8.63 to 13.27) | 10.49 (8.49 to 12.96) | 10.88 (8.67 to 13.62) | 92.7 (84.5 to 100.8) | 93.9 (82.5 to 106.8) | 90.8 (80.8 to 101.1) |
|  | **Prevalence** | 192.26 (150.9 to 242.81) | 187.24 (146.89 to 235.59) | 197.05 (153.76 to 248.28) | 379.13 (296.72 to 473) | 370.56 (291.54 to 460.97) | 387.24 (301.13 to 484.91) | 97.2 (88 to 106.5) | 97.9 (85.3 to 112.7) | 96.5 (85.8 to 109) |
|  | **Deaths** | 1.2 (0.72 to 1.6) | 1.31 (0.68 to 1.92) | 1.09 (0.65 to 1.62) | 0.76 (0.64 to 0.9) | 0.71 (0.57 to 0.88) | 0.81 (0.64 to 1.02) | -36.6 (-55.3 to 4.8) | -46 (-65.3 to 3.9) | -25.7 (-53.8 to 25.9) |
|  | **DALYs** | 53.75 (38.89 to 67.91) | 57.8 (38.15 to 78.69) | 49.96 (36.46 to 66.25) | 50.12 (39.48 to 63.15) | 48.06 (37.58 to 61.62) | 52.18 (41.01 to 66.18) | -6.8 (-28.3 to 24.5) | -16.8 (-41.6 to 22.7) | 4.4 (-26.8 to 40.3) |
|  | **YLLs** | 39.51 (26.46 to 52.2) | 43.81 (25.66 to 63.97) | 35.51 (23.69 to 50.65) | 22.38 (18.78 to 25.96) | 20.62 (16.18 to 24.99) | 24.13 (19.21 to 29.83) | -43.4 (-59 to -14.1) | -52.9 (-68.5 to -20.1) | -32 (-57.3 to 7.4) |
|  | **YLDs** | 14.24 (9.26 to 20.99) | 13.98 (9.11 to 20.79) | 14.46 (9.3 to 21.09) | 27.74 (18.07 to 40.27) | 27.44 (17.68 to 40.09) | 28.04 (17.88 to 40.76) | 94.8 (79.9 to 111) | 96.2 (74.9 to 120.4) | 94 (73.6 to 115.8) |
| **Bushehr** | **Incidence** | 5.76 (4.65 to 7.12) | 5.63 (4.52 to 6.92) | 5.89 (4.71 to 7.29) | 10.83 (8.7 to 13.42) | 10.64 (8.57 to 13.27) | 10.98 (8.73 to 13.6) | 88.1 (80.1 to 97.2) | 89.2 (77.9 to 101.2) | 86.4 (77 to 96) |
|  | **Prevalence** | 198.42 (155.98 to 247.33) | 193.64 (151.78 to 239.9) | 202.99 (159.6 to 253.38) | 383.88 (299.49 to 477.41) | 375.64 (294.04 to 469.22) | 391.09 (303.72 to 484.21) | 93.5 (84.5 to 104.1) | 94 (80.8 to 107.8) | 92.7 (81.8 to 105.2) |
|  | **Deaths** | 1.14 (0.71 to 1.49) | 1.1 (0.67 to 1.58) | 1.17 (0.66 to 1.64) | 0.79 (0.61 to 0.93) | 0.71 (0.51 to 0.91) | 0.86 (0.6 to 1.08) | -30.7 (-49 to 7) | -35.9 (-60.2 to 9) | -25.9 (-50.3 to 26.8) |
|  | **DALYs** | 46.89 (36.07 to 58.52) | 45.9 (33.06 to 60.04) | 47.77 (34.22 to 61.22) | 51.11 (40.39 to 65.06) | 48.63 (37.1 to 61.92) | 53.41 (41.35 to 68.98) | 9 (-13.8 to 41.9) | 5.9 (-20.5 to 43.2) | 11.8 (-15.8 to 47.7) |
|  | **YLLs** | 32.17 (22.17 to 41.1) | 31.41 (20.08 to 43.68) | 32.85 (21.13 to 44.49) | 23.01 (17.78 to 27.07) | 20.81 (12.98 to 25.88) | 25.04 (18.81 to 30.89) | -28.5 (-47.2 to 5.8) | -33.7 (-61.5 to 6.1) | -23.8 (-48.7 to 20.7) |
|  | **YLDs** | 14.72 (9.58 to 21.48) | 14.5 (9.45 to 21.19) | 14.92 (9.72 to 21.7) | 28.1 (18.39 to 41.2) | 27.82 (18.07 to 40.33) | 28.37 (18.59 to 41.86) | 90.9 (76.5 to 106.3) | 91.9 (71 to 116.2) | 90.1 (70.5 to 111.1) |
| **Chahar Mahaal and Bakhtiari** | **Incidence** | 5.66 (4.53 to 7.01) | 5.52 (4.4 to 6.82) | 5.8 (4.65 to 7.18) | 10.75 (8.67 to 13.17) | 10.54 (8.5 to 12.98) | 10.93 (8.77 to 13.43) | 89.9 (81.2 to 98.7) | 91.1 (81.3 to 104.1) | 88.4 (77.5 to 102) |
|  | **Prevalence** | 196.12 (153.08 to 244.95) | 190.92 (147.89 to 239.21) | 200.95 (157.36 to 250.63) | 382.11 (299.14 to 473.5) | 373.41 (293.87 to 466.72) | 390.39 (305.72 to 483.82) | 94.8 (84.5 to 105.3) | 95.6 (83.8 to 110.6) | 94.3 (82 to 109.7) |
|  | **Deaths** | 0.87 (0.64 to 1.21) | 0.76 (0.46 to 1.22) | 0.98 (0.7 to 1.34) | 0.58 (0.46 to 0.75) | 0.43 (0.31 to 0.67) | 0.73 (0.54 to 0.95) | -34.1 (-52.4 to -5.8) | -44.1 (-63.7 to -4.8) | -24.9 (-51.1 to 12.9) |
|  | **DALYs** | 39.92 (31.95 to 50.06) | 36.3 (27.4 to 50.07) | 43.19 (33.13 to 54.57) | 44.41 (34.27 to 58.31) | 39.81 (29.94 to 53) | 49.03 (37.74 to 63.44) | 11.2 (-10.9 to 35.3) | 9.7 (-16.5 to 42.6) | 13.5 (-13.5 to 44.9) |
|  | **YLLs** | 25.32 (19.57 to 34.64) | 21.91 (14.53 to 33.59) | 28.4 (20.69 to 38.24) | 16.3 (13.31 to 20.32) | 12 (8.94 to 17.09) | 20.62 (15.62 to 26.13) | -35.6 (-52.9 to -9.4) | -45.2 (-64 to -12.8) | -27.4 (-52.2 to 9.7) |
|  | **YLDs** | 14.6 (9.44 to 21.59) | 14.39 (9.44 to 21.3) | 14.79 (9.54 to 21.73) | 28.11 (18.42 to 41.23) | 27.81 (17.92 to 40.5) | 28.41 (18.75 to 41.67) | 92.5 (77.9 to 108.8) | 93.3 (71.4 to 116.5) | 92.1 (71.7 to 118.2) |
| **East Azarbayejan** | **Incidence** | 5.69 (4.62 to 7.02) | 5.56 (4.45 to 6.87) | 5.82 (4.71 to 7.15) | 10.73 (8.62 to 13.19) | 10.53 (8.52 to 12.98) | 10.91 (8.74 to 13.33) | 88.6 (81.7 to 97.8) | 89.4 (79.7 to 100.5) | 87.4 (78.3 to 99.5) |
|  | **Prevalence** | 196.1 (154.42 to 245.48) | 191.47 (149.31 to 240.76) | 200.51 (157.57 to 248.99) | 380.18 (299.05 to 468.55) | 371.81 (292.79 to 463.96) | 388.23 (305.17 to 479.56) | 93.9 (86 to 104.8) | 94.2 (83 to 106.8) | 93.6 (83.3 to 107.6) |
|  | **Deaths** | 1.14 (0.84 to 1.45) | 1.16 (0.74 to 1.64) | 1.11 (0.8 to 1.46) | 0.73 (0.6 to 0.96) | 0.72 (0.56 to 1.05) | 0.74 (0.56 to 1.01) | -36.3 (-53.3 to -8.9) | -38.1 (-57.8 to 1.2) | -33.8 (-56 to 3) |
|  | **DALYs** | 48.01 (38.55 to 60.33) | 48.01 (35.88 to 62.24) | 47.87 (37.17 to 60.48) | 50.17 (39.98 to 63.38) | 48.69 (37.89 to 62.45) | 51.65 (40.3 to 66.16) | 4.5 (-16.9 to 28.3) | 1.4 (-26.5 to 31.8) | 7.9 (-18 to 36.3) |
|  | **YLLs** | 33.43 (25.75 to 43.52) | 33.64 (23.3 to 47.73) | 33.12 (24.08 to 44.14) | 22.32 (18.77 to 28.02) | 21.14 (16.46 to 27.7) | 23.49 (18.16 to 31.35) | -33.2 (-50.7 to -8.2) | -37.1 (-58.4 to -1.8) | -29.1 (-51.8 to 4.2) |
|  | **YLDs** | 14.57 (9.48 to 21.37) | 14.37 (9.13 to 21.26) | 14.75 (9.66 to 21.63) | 27.85 (18.24 to 40.27) | 27.54 (17.73 to 39.98) | 28.15 (18.32 to 41.02) | 91.1 (76.6 to 108.6) | 91.7 (72.7 to 116.3) | 90.8 (71.3 to 114.2) |
| **Fars** | **Incidence** | 5.15 (4.05 to 6.5) | 5.07 (4 to 6.36) | 5.22 (4.09 to 6.55) | 10.16 (7.9 to 12.64) | 9.95 (7.7 to 12.32) | 10.34 (7.98 to 12.91) | 97.3 (79.6 to 111.8) | 96.3 (75.4 to 112.4) | 98.3 (80.4 to 114.3) |
|  | **Prevalence** | 179.76 (138.72 to 228.42) | 176.52 (136.79 to 224.08) | 182.77 (140.85 to 233.19) | 366.26 (280.36 to 459.47) | 357.84 (272.58 to 449) | 374.38 (285.25 to 473.1) | 103.7 (86.6 to 117.1) | 102.7 (82.5 to 120.5) | 104.8 (85.9 to 122.9) |
|  | **Deaths** | 1.27 (0.83 to 1.64) | 1.24 (0.75 to 1.74) | 1.27 (0.77 to 1.81) | 0.85 (0.67 to 1.04) | 0.81 (0.59 to 1.04) | 0.9 (0.65 to 1.16) | -32.6 (-50.5 to -2.6) | -34.4 (-57.3 to 6.8) | -29.6 (-54.3 to 10.5) |
|  | **DALYs** | 48 (37.5 to 58.78) | 46.35 (34.55 to 60.04) | 49.31 (35.93 to 62.89) | 53.79 (42.57 to 66.88) | 50.45 (38.95 to 64.51) | 57.05 (43.82 to 70.99) | 12.1 (-12.6 to 42.1) | 8.8 (-20 to 47.1) | 15.7 (-14.9 to 52.2) |
|  | **YLLs** | 34.71 (25.89 to 44.16) | 33.13 (22.58 to 44.81) | 35.96 (23.9 to 49.1) | 27.13 (21.76 to 32.16) | 24.09 (17.33 to 30.55) | 30.08 (22.8 to 37.9) | -21.9 (-42 to 6.5) | -27.3 (-53.4 to 13.8) | -16.4 (-44.5 to 26.3) |
|  | **YLDs** | 13.29 (8.52 to 19.94) | 13.22 (8.53 to 19.62) | 13.35 (8.48 to 20.04) | 26.66 (17.02 to 39.48) | 26.35 (16.58 to 38.33) | 26.97 (17.11 to 40.45) | 100.6 (80.3 to 121.7) | 99.4 (72.2 to 129) | 102 (74.6 to 128.7) |
| **Gilan** | **Incidence** | 5.98 (4.82 to 7.4) | 5.82 (4.7 to 7.19) | 6.12 (4.92 to 7.61) | 11.48 (9.24 to 14) | 11.29 (9.05 to 13.94) | 11.65 (9.29 to 14.2) | 92.1 (82.4 to 100.4) | 94 (82.2 to 108.3) | 90.3 (79.2 to 101.4) |
|  | **Prevalence** | 204.77 (160.82 to 256.6) | 199.3 (156.06 to 251.27) | 210.1 (164.68 to 263.01) | 402.33 (313.65 to 499.03) | 394.14 (310.03 to 493.22) | 410.44 (318.72 to 506.83) | 96.5 (84.8 to 106.5) | 97.8 (83.9 to 113.4) | 95.4 (81.7 to 108.1) |
|  | **Deaths** | 0.97 (0.73 to 1.32) | 0.96 (0.64 to 1.47) | 0.96 (0.71 to 1.28) | 0.7 (0.57 to 0.9) | 0.68 (0.52 to 0.95) | 0.71 (0.53 to 0.98) | -28.1 (-49.7 to 4.3) | -28.4 (-58.4 to 17.7) | -25.9 (-51.6 to 16.8) |
|  | **DALYs** | 43 (34.34 to 55.65) | 42.43 (32.48 to 58.33) | 43.33 (33.67 to 55.1) | 50.59 (39.46 to 65.52) | 48.58 (36.33 to 63.73) | 52.62 (40.41 to 68.01) | 17.7 (-7.3 to 44.1) | 14.5 (-17.9 to 48.7) | 21.4 (-5.6 to 54.1) |
|  | **YLLs** | 27.75 (21.21 to 36.96) | 27.43 (18.99 to 40.02) | 27.85 (20.58 to 37.83) | 21.06 (17.4 to 26.55) | 19.33 (14.78 to 25.4) | 22.81 (17.43 to 30.91) | -24.1 (-45.8 to 5.4) | -29.5 (-60.1 to 11.3) | -18.1 (-45.1 to 24.1) |
|  | **YLDs** | 15.24 (10.04 to 22.4) | 15 (9.63 to 22.29) | 15.48 (9.93 to 22.84) | 29.53 (19.36 to 43.51) | 29.25 (18.9 to 43.12) | 29.81 (19.16 to 43.71) | 93.7 (78.4 to 108.9) | 95 (74.5 to 117.5) | 92.6 (72.2 to 112.7) |
| **Golestan** | **Incidence** | 5.77 (4.66 to 7.13) | 5.63 (4.54 to 6.98) | 5.93 (4.74 to 7.29) | 10.76 (8.63 to 13.25) | 10.55 (8.46 to 13.03) | 10.95 (8.69 to 13.49) | 86.3 (79 to 94.8) | 87.5 (76.1 to 98.8) | 84.7 (74.5 to 95) |
|  | **Prevalence** | 197.73 (155.52 to 246.79) | 192.56 (150.91 to 241.82) | 202.69 (160.13 to 254.81) | 380.05 (295.78 to 473.57) | 371.37 (291.25 to 468.32) | 388.7 (301.74 to 482.14) | 92.2 (83.5 to 102.3) | 92.9 (78.9 to 105.1) | 91.8 (79.6 to 104.2) |
|  | **Deaths** | 1.37 (0.9 to 1.77) | 1.39 (0.84 to 1.96) | 1.33 (0.79 to 1.91) | 1.05 (0.79 to 1.25) | 1.04 (0.73 to 1.31) | 1.06 (0.75 to 1.35) | -22.9 (-43.6 to 10.6) | -25.1 (-53.9 to 24.1) | -19.9 (-46.5 to 21.6) |
|  | **DALYs** | 53.96 (41.69 to 66.63) | 54.52 (40.48 to 71.49) | 53.32 (38.78 to 69.2) | 60.77 (47.15 to 75.11) | 58.88 (44.52 to 73.05) | 62.66 (48.44 to 78.58) | 12.6 (-12 to 39.9) | 8 (-24.3 to 44.3) | 17.5 (-10.3 to 50.1) |
|  | **YLLs** | 39.3 (28.63 to 50.19) | 40.1 (27.04 to 55.74) | 38.42 (26.09 to 52.23) | 32.96 (24.98 to 38.71) | 31.4 (20.8 to 38.63) | 34.51 (25.65 to 43.22) | -16.1 (-40 to 16.4) | -21.7 (-56.6 to 20.2) | -10.2 (-38.3 to 31.8) |
|  | **YLDs** | 14.67 (9.62 to 21.58) | 14.41 (9.44 to 21.36) | 14.9 (9.69 to 22.12) | 27.8 (18.08 to 40.82) | 27.48 (17.83 to 40.35) | 28.14 (18.16 to 41.12) | 89.6 (74.1 to 105.6) | 90.7 (70.1 to 111.3) | 88.9 (68.9 to 111) |
| **Hamadan** | **Incidence** | 5.69 (4.59 to 7.02) | 5.54 (4.43 to 6.88) | 5.83 (4.67 to 7.22) | 10.94 (8.79 to 13.56) | 10.73 (8.66 to 13.22) | 11.13 (8.86 to 13.81) | 92.4 (82.8 to 101.5) | 93.8 (82.9 to 106.4) | 90.9 (80.3 to 102.4) |
|  | **Prevalence** | 195.86 (153.02 to 244.58) | 190.73 (147.68 to 240.37) | 200.75 (157.24 to 251.64) | 385.87 (302.63 to 480.39) | 377.23 (297.1 to 468.04) | 394.29 (305.48 to 491.22) | 97 (86.7 to 107.2) | 97.8 (85.7 to 112.4) | 96.4 (83.7 to 109.1) |
|  | **Deaths** | 1.04 (0.76 to 1.39) | 1.03 (0.65 to 1.54) | 1.04 (0.75 to 1.42) | 0.7 (0.57 to 0.88) | 0.66 (0.51 to 0.97) | 0.74 (0.57 to 0.97) | -32.7 (-50.1 to -4.8) | -35.7 (-57.1 to 8.5) | -28.8 (-52.5 to 8.3) |
|  | **DALYs** | 44.52 (35.89 to 57.3) | 43.06 (32.64 to 58.85) | 45.87 (35.55 to 59.72) | 48.71 (38.33 to 61.76) | 46.38 (35.84 to 60.07) | 51.02 (39.23 to 65) | 9.4 (-13.9 to 32.4) | 7.7 (-23 to 39.8) | 11.2 (-15.3 to 42.6) |
|  | **YLLs** | 30.55 (23.59 to 41.71) | 29.02 (19.88 to 42.33) | 31.98 (23.33 to 43.44) | 21.47 (17.94 to 26.36) | 18.83 (14.96 to 25.22) | 24.07 (18.63 to 30.82) | -29.7 (-47.6 to -6.5) | -35.1 (-55.8 to 0.2) | -24.8 (-48.5 to 9.9) |
|  | **YLDs** | 13.97 (9 to 20.46) | 14.04 (9.01 to 20.57) | 13.89 (8.9 to 20.39) | 27.24 (17.89 to 40.19) | 27.55 (17.61 to 40.07) | 26.95 (17.64 to 40.47) | 95.1 (80.2 to 112.1) | 96.2 (75.3 to 120.5) | 94.1 (73.2 to 119.2) |
| **Hormozgan** | **Incidence** | 5.72 (4.6 to 7.1) | 5.58 (4.47 to 6.92) | 5.87 (4.68 to 7.3) | 10.67 (8.59 to 13.18) | 10.45 (8.39 to 12.91) | 10.87 (8.72 to 13.49) | 86.5 (79.6 to 94.8) | 87.5 (76.7 to 98.3) | 85.2 (73.9 to 97.3) |
|  | **Prevalence** | 195.94 (153.49 to 247.13) | 190.91 (149.18 to 239.01) | 200.43 (156.66 to 251.28) | 378.16 (295.71 to 472.87) | 369.08 (288.13 to 463.15) | 386.84 (303.07 to 483.85) | 93 (85.2 to 102.5) | 93.3 (79.8 to 105.9) | 93 (80.2 to 106.5) |
|  | **Deaths** | 1.31 (0.78 to 1.77) | 1.36 (0.75 to 2.02) | 1.26 (0.72 to 1.81) | 0.8 (0.59 to 0.96) | 0.8 (0.56 to 1.04) | 0.81 (0.56 to 1.04) | -38.8 (-56.4 to -3.4) | -40.9 (-62.8 to 7.3) | -35.6 (-57.7 to 6.4) |
|  | **DALYs** | 53.23 (37.72 to 67.32) | 54 (36.57 to 74.16) | 52.32 (37.19 to 69.19) | 52.48 (41.16 to 66.07) | 49.51 (37.69 to 63.98) | 55.34 (42.67 to 70.89) | -1.4 (-23.1 to 36.8) | -8.3 (-34.7 to 34.4) | 5.8 (-20.6 to 48.1) |
|  | **YLLs** | 38.71 (24.76 to 50.64) | 39.71 (22.83 to 57.99) | 37.62 (23.14 to 52.64) | 24.83 (19.26 to 29.45) | 22.18 (15.41 to 27.9) | 27.35 (20.9 to 33.55) | -35.9 (-53 to 2.7) | -44.1 (-64.3 to -1.2) | -27.3 (-51.4 to 20.3) |
|  | **YLDs** | 14.51 (9.42 to 21.44) | 14.29 (9.12 to 20.99) | 14.7 (9.42 to 21.76) | 27.66 (18.27 to 40.26) | 27.33 (17.94 to 39.97) | 27.99 (18.39 to 41.38) | 90.6 (75.8 to 106.4) | 91.3 (70.9 to 113.8) | 90.4 (70.5 to 112.8) |
| **Ilam** | **Incidence** | 5.65 (4.55 to 6.98) | 5.52 (4.44 to 6.78) | 5.79 (4.63 to 7.15) | 11.44 (9.19 to 14.12) | 11.27 (9.06 to 13.9) | 11.6 (9.33 to 14.23) | 102.4 (92.9 to 111.3) | 104.1 (92.5 to 116.3) | 100.3 (88.3 to 111.7) |
|  | **Prevalence** | 195.55 (152.93 to 244.36) | 190.67 (148.77 to 238.65) | 199.85 (155.43 to 248.84) | 401.02 (316.02 to 496.04) | 392.97 (307.52 to 491.49) | 408.94 (322.36 to 507.25) | 105.1 (94.8 to 115.2) | 106.1 (92.1 to 119.1) | 104.6 (90.4 to 117.5) |
|  | **Deaths** | 1.08 (0.7 to 1.43) | 1.02 (0.6 to 1.57) | 1.13 (0.68 to 1.58) | 0.84 (0.66 to 0.99) | 0.78 (0.61 to 1.01) | 0.89 (0.63 to 1.11) | -22.8 (-42.8 to 11.9) | -23.9 (-49.9 to 27.8) | -21.4 (-46.7 to 23.6) |
|  | **DALYs** | 44.64 (35 to 55.18) | 42.57 (31.98 to 55.92) | 46.22 (34.68 to 59.17) | 53.58 (41.74 to 67.81) | 50.49 (38.96 to 65.32) | 56.52 (44.36 to 70.88) | 20 (-7.6 to 47.4) | 18.6 (-15 to 54.9) | 22.3 (-6.1 to 58.8) |
|  | **YLLs** | 30.08 (22.09 to 38.9) | 28.26 (18.62 to 41.26) | 31.47 (20.97 to 41.92) | 24.08 (19.98 to 27.96) | 21.26 (17.13 to 26.18) | 26.75 (20.82 to 32.73) | -19.9 (-42.1 to 11.5) | -24.8 (-50.5 to 16) | -15 (-41.1 to 26.7) |
|  | **YLDs** | 14.56 (9.47 to 21.36) | 14.32 (9.2 to 21.16) | 14.75 (9.63 to 21.58) | 29.5 (19.38 to 43.38) | 29.24 (18.82 to 42.91) | 29.77 (19.54 to 43.47) | 102.6 (85.6 to 120.3) | 104.2 (81.6 to 128.6) | 101.9 (80.1 to 127) |
| **Isfahan** | **Incidence** | 5.87 (4.73 to 7.24) | 5.72 (4.57 to 7.07) | 6.01 (4.82 to 7.4) | 11.21 (9.01 to 13.81) | 11.01 (8.88 to 13.6) | 11.4 (9.11 to 14.05) | 90.9 (83.5 to 99.3) | 92.5 (82.3 to 102.9) | 89.8 (78.5 to 101.5) |
|  | **Prevalence** | 202.26 (158.33 to 252) | 197.03 (154.47 to 247.76) | 207.05 (161.73 to 257.86) | 395.05 (309.82 to 494.5) | 386.18 (306.81 to 486.86) | 403.67 (314.29 to 504.67) | 95.3 (86.5 to 104.6) | 96 (83.9 to 108.6) | 95 (81.7 to 108.2) |
|  | **Deaths** | 1.23 (0.73 to 1.66) | 1.26 (0.69 to 1.8) | 1.18 (0.65 to 1.69) | 0.85 (0.55 to 1.03) | 0.84 (0.46 to 1.1) | 0.86 (0.56 to 1.14) | -31.3 (-51.1 to 1.2) | -33.6 (-58.6 to 9.9) | -26.8 (-51.2 to 24.9) |
|  | **DALYs** | 48.18 (35.42 to 60.06) | 47.34 (33.37 to 61.46) | 48.63 (35.4 to 62.67) | 54.73 (41.25 to 69.8) | 52.15 (37.8 to 67.72) | 57.28 (42.71 to 74.79) | 13.6 (-10.1 to 43.5) | 10.2 (-17.9 to 45) | 17.8 (-10.7 to 56.2) |
|  | **YLLs** | 33.14 (22.61 to 43.03) | 32.53 (20.18 to 45.2) | 33.38 (22.16 to 45.66) | 25.72 (17.31 to 30.91) | 23.44 (13.06 to 30.08) | 27.96 (19.25 to 35.67) | -22.4 (-45.3 to 9.1) | -27.9 (-57 to 13.2) | -16.2 (-43.3 to 32.7) |
|  | **YLDs** | 15.04 (9.84 to 22.12) | 14.81 (9.58 to 21.96) | 15.25 (9.95 to 22.49) | 29.01 (18.78 to 42.59) | 28.7 (18.53 to 42.35) | 29.32 (19.06 to 43.31) | 92.9 (78.1 to 108.3) | 93.8 (74.8 to 117) | 92.2 (70.7 to 114) |
| **Kerman** | **Incidence** | 5.91 (4.75 to 7.31) | 5.77 (4.62 to 7.17) | 6.06 (4.84 to 7.46) | 10.91 (8.76 to 13.35) | 10.7 (8.59 to 13.15) | 11.11 (8.83 to 13.68) | 84.5 (76.5 to 92) | 85.4 (73.9 to 96.5) | 83.4 (73.7 to 93.5) |
|  | **Prevalence** | 202.01 (157.44 to 254.18) | 197.12 (153.3 to 250.44) | 206.59 (160.36 to 256.84) | 384.64 (300.22 to 474.73) | 375.79 (294.43 to 466.56) | 392.97 (307.4 to 486.05) | 90.4 (81 to 99.7) | 90.6 (76.2 to 104.6) | 90.2 (79.4 to 102.3) |
|  | **Deaths** | 1.47 (0.94 to 1.94) | 1.43 (0.86 to 2.03) | 1.47 (0.86 to 2.06) | 0.96 (0.71 to 1.15) | 0.96 (0.71 to 1.19) | 0.97 (0.63 to 1.24) | -34.2 (-50.5 to -4.9) | -33 (-55.2 to 9.6) | -34.5 (-55.2 to 0.5) |
|  | **DALYs** | 54.29 (41.81 to 67.7) | 52.52 (38.88 to 69.01) | 55.72 (40.1 to 72.38) | 55.95 (43.93 to 70.89) | 54.42 (42.45 to 68.95) | 57.41 (43.9 to 73) | 3.1 (-17.7 to 27.5) | 3.6 (-24.1 to 35.9) | 3 (-23.7 to 33.8) |
|  | **YLLs** | 39.35 (28.74 to 50.19) | 37.78 (25.26 to 52.33) | 40.58 (26.95 to 55.16) | 27.9 (21.58 to 33.05) | 26.68 (19.75 to 32.89) | 29.04 (21.47 to 36.41) | -29.1 (-47.4 to -3.3) | -29.4 (-53.3 to 7.3) | -28.4 (-51.4 to 6.7) |
|  | **YLDs** | 14.95 (9.77 to 22.11) | 14.74 (9.49 to 22.08) | 15.13 (9.82 to 22.47) | 28.06 (18.1 to 41.46) | 27.74 (17.69 to 40.41) | 28.37 (18.25 to 41.79) | 87.7 (72.6 to 103.7) | 88.2 (67.2 to 111.1) | 87.4 (68 to 109.5) |
| **Kermanshah** | **Incidence** | 5.76 (4.62 to 7.14) | 5.62 (4.47 to 6.97) | 5.91 (4.76 to 7.31) | 10.81 (8.71 to 13.31) | 10.62 (8.54 to 13.16) | 10.99 (8.81 to 13.43) | 87.6 (79.6 to 96.3) | 89 (77.7 to 101.5) | 85.7 (76.8 to 97.7) |
|  | **Prevalence** | 197.72 (155.85 to 247.55) | 192.56 (150.53 to 242.17) | 202.49 (159.56 to 253.24) | 381.88 (300.14 to 474.15) | 374.09 (292.63 to 469.82) | 389.61 (306.15 to 483.23) | 93.1 (84.1 to 103.2) | 94.3 (79.9 to 108.5) | 92.4 (82.4 to 106.7) |
|  | **Deaths** | 1.38 (0.9 to 1.83) | 1.32 (0.77 to 1.94) | 1.43 (0.86 to 2.04) | 0.85 (0.71 to 1.01) | 0.83 (0.65 to 1.08) | 0.88 (0.66 to 1.13) | -38.1 (-55.2 to -7.7) | -37 (-60.1 to 9.3) | -38.3 (-59.9 to -3.4) |
|  | **DALYs** | 53.15 (41.03 to 65.67) | 50.76 (36.58 to 67.22) | 55.16 (41.33 to 71.01) | 54.19 (43.45 to 67.02) | 51.81 (40.29 to 65.63) | 56.65 (44.62 to 71.38) | 2 (-20.1 to 29.2) | 2.1 (-27.1 to 40.5) | 2.7 (-23.1 to 36.4) |
|  | **YLLs** | 38.49 (28.27 to 49.29) | 36.34 (23.48 to 51.45) | 40.31 (27.35 to 55.32) | 26.22 (21.99 to 30.93) | 24.13 (19.1 to 30.41) | 28.38 (22.25 to 35.56) | -31.9 (-50.4 to -3.2) | -33.6 (-56.5 to 8.7) | -29.6 (-53 to 5.3) |
|  | **YLDs** | 14.66 (9.48 to 21.58) | 14.42 (9.29 to 21.15) | 14.85 (9.75 to 21.74) | 27.97 (18.26 to 40.64) | 27.68 (18.21 to 40.27) | 28.27 (18.19 to 41.07) | 90.8 (75.9 to 108.1) | 92 (70.7 to 118.6) | 90.4 (69.9 to 113.7) |
| **Khorasan-e-Razavi** | **Incidence** | 5.8 (4.65 to 7.18) | 5.65 (4.51 to 7.02) | 5.95 (4.76 to 7.34) | 10.84 (8.7 to 13.42) | 10.64 (8.56 to 13.17) | 11.03 (8.82 to 13.62) | 86.9 (80.2 to 94.5) | 88.2 (78.3 to 100.5) | 85.3 (75.2 to 97.3) |
|  | **Prevalence** | 198.45 (156.56 to 249.45) | 193.23 (151.88 to 244.98) | 203.44 (159.83 to 254.21) | 382.94 (299.81 to 476.11) | 374.13 (293.48 to 468.33) | 391.59 (306.05 to 488.48) | 93 (84.9 to 102.7) | 93.6 (81.2 to 106.9) | 92.5 (80.9 to 106.6) |
|  | **Deaths** | 1.43 (0.95 to 1.88) | 1.42 (0.86 to 2.08) | 1.42 (0.88 to 2.04) | 0.88 (0.72 to 1.04) | 0.85 (0.67 to 1.09) | 0.91 (0.66 to 1.16) | -38.3 (-55.7 to -9.5) | -40.4 (-63.5 to 3.3) | -35.8 (-56.9 to -1.7) |
|  | **DALYs** | 54.49 (42.12 to 67.74) | 53.56 (39.2 to 71.07) | 55.24 (41.07 to 71.06) | 54.11 (43.42 to 67.36) | 51.8 (40.28 to 65.13) | 56.39 (43.97 to 70.78) | -0.7 (-23.6 to 24.2) | -3.3 (-31.9 to 32.2) | 2.1 (-23.2 to 34) |
|  | **YLLs** | 39.77 (29.51 to 50.51) | 39.06 (25.97 to 55.26) | 40.34 (27.76 to 54.55) | 26.1 (21.71 to 30.39) | 24.08 (18.86 to 30.23) | 28.1 (21.4 to 34.49) | -34.4 (-53 to -8.6) | -38.4 (-62.1 to -1.8) | -30.3 (-52.1 to 4.3) |
|  | **YLDs** | 14.72 (9.38 to 21.53) | 14.5 (9.16 to 21.28) | 14.9 (9.46 to 21.93) | 28 (18.36 to 40.34) | 27.72 (18.1 to 39.99) | 28.29 (18.55 to 41.59) | 90.3 (76.4 to 105.9) | 91.1 (71.2 to 115.1) | 89.8 (70.8 to 111.4) |
| **Khuzestan** | **Incidence** | 5.67 (4.55 to 7.02) | 5.52 (4.41 to 6.82) | 5.82 (4.64 to 7.22) | 10.47 (8.45 to 12.86) | 10.26 (8.35 to 12.63) | 10.67 (8.5 to 13.11) | 84.5 (76.9 to 93.9) | 85.8 (75.1 to 99.4) | 83.3 (73 to 94.6) |
|  | **Prevalence** | 195.74 (152.87 to 243.43) | 190.37 (147.73 to 237.89) | 200.76 (157.77 to 252.04) | 372.98 (293.99 to 463.59) | 364.23 (288.61 to 456.72) | 381.49 (299.42 to 474.71) | 90.5 (81.8 to 101.2) | 91.3 (79 to 106.9) | 90 (77.9 to 102.8) |
|  | **Deaths** | 1.64 (0.84 to 2.21) | 1.74 (0.7 to 2.54) | 1.51 (0.74 to 2.25) | 1.18 (0.65 to 1.49) | 1.22 (0.45 to 1.62) | 1.14 (0.66 to 1.55) | -27.9 (-45.7 to 6.3) | -29.6 (-53 to 20.7) | -25 (-49.5 to 13.3) |
|  | **DALYs** | 64.77 (42.16 to 82.21) | 67.07 (37.12 to 92.67) | 62.3 (40.56 to 83.21) | 66.24 (45.53 to 83.01) | 65.61 (41.76 to 82.65) | 66.79 (47.62 to 85.69) | 2.3 (-18.2 to 32.5) | -2.2 (-28.7 to 37.6) | 7.2 (-20.9 to 41.9) |
|  | **YLLs** | 50.36 (29.14 to 66.06) | 52.9 (23.69 to 76.49) | 47.67 (27.9 to 68.24) | 39.1 (21.71 to 48.4) | 38.81 (12.99 to 50.5) | 39.32 (24.35 to 52.04) | -22.3 (-41.7 to 8.9) | -26.6 (-51.8 to 16.3) | -17.5 (-44 to 19.6) |
|  | **YLDs** | 14.41 (9.27 to 21.22) | 14.17 (9.21 to 20.98) | 14.63 (9.28 to 21.57) | 27.14 (17.72 to 39.1) | 26.8 (17.46 to 38.84) | 27.47 (17.82 to 40.1) | 88.3 (73.6 to 103.8) | 89.1 (69.2 to 112.2) | 87.8 (68.2 to 108.4) |
| **Kohgiluyeh and Boyer-Ahmad** | **Incidence** | 5.57 (4.47 to 6.88) | 5.42 (4.33 to 6.75) | 5.72 (4.57 to 7.08) | 10.6 (8.5 to 13.15) | 10.4 (8.38 to 12.92) | 10.79 (8.56 to 13.36) | 90.4 (81.9 to 99.8) | 92 (81.2 to 105.6) | 88.9 (77 to 101.4) |
|  | **Prevalence** | 192.8 (150.61 to 240.81) | 187.24 (146.56 to 235.93) | 197.76 (154.77 to 248.27) | 377.07 (293.69 to 467.46) | 368.22 (288.65 to 457.44) | 385.71 (300.6 to 483.13) | 95.6 (85.6 to 106.6) | 96.7 (84.1 to 112.5) | 95 (81.6 to 109.4) |
|  | **Deaths** | 1 (0.65 to 1.34) | 1.14 (0.63 to 1.68) | 0.83 (0.54 to 1.18) | 0.61 (0.49 to 0.8) | 0.7 (0.5 to 1.03) | 0.55 (0.41 to 0.76) | -38.6 (-58.5 to -2.2) | -39.2 (-62.7 to 12.4) | -33.3 (-59.4 to 13.6) |
|  | **DALYs** | 43.43 (34.66 to 53.62) | 46.19 (34.12 to 60.72) | 40.55 (31.1 to 51.8) | 46.6 (36.22 to 59.88) | 46.89 (36.06 to 60.14) | 46.55 (35.24 to 60.73) | 7.3 (-14.3 to 35) | 1.5 (-25.9 to 39.5) | 14.8 (-14.7 to 47.5) |
|  | **YLLs** | 29.17 (21.63 to 37.38) | 32.16 (20.7 to 45.17) | 26.09 (18.49 to 35.08) | 18.97 (15.45 to 23.59) | 19.6 (14.75 to 26.78) | 18.6 (13.88 to 24.78) | -35 (-53.4 to -5.6) | -39.1 (-60 to -2.1) | -28.7 (-54 to 12.1) |
|  | **YLDs** | 14.26 (9.21 to 21.21) | 14.02 (8.93 to 20.72) | 14.46 (9.3 to 22.02) | 27.63 (18.04 to 40.34) | 27.29 (17.63 to 39.87) | 27.95 (18.29 to 41.02) | 93.8 (78.5 to 111) | 94.6 (73.5 to 120.8) | 93.3 (71.6 to 117.6) |
| **Kurdistan** | **Incidence** | 5.52 (4.45 to 6.79) | 5.38 (4.34 to 6.65) | 5.66 (4.55 to 6.96) | 10.33 (8.26 to 12.78) | 10.14 (8.09 to 12.55) | 10.5 (8.37 to 13.09) | 87.1 (78.5 to 95.2) | 88.4 (78 to 99.1) | 85.3 (74.9 to 96.7) |
|  | **Prevalence** | 191.16 (149.64 to 238.7) | 186.36 (145.81 to 234.64) | 195.64 (153.21 to 243.83) | 368.4 (288.82 to 458.79) | 360.8 (281.99 to 452.47) | 375.81 (294.35 to 471.25) | 92.7 (83.4 to 102.6) | 93.6 (80.1 to 106.8) | 92.1 (80 to 105.6) |
|  | **Deaths** | 1.04 (0.78 to 1.37) | 0.99 (0.59 to 1.47) | 1.08 (0.77 to 1.53) | 0.59 (0.48 to 0.79) | 0.57 (0.43 to 0.8) | 0.62 (0.45 to 0.91) | -43.3 (-58 to -14.3) | -43.1 (-62.9 to 1.1) | -42.7 (-63.2 to -9.2) |
|  | **DALYs** | 45.48 (36.09 to 59.36) | 43.52 (32.08 to 58.75) | 47.18 (35.65 to 63.54) | 45.11 (34.67 to 57.88) | 42.65 (32.61 to 56.02) | 47.54 (36.51 to 61.9) | -0.8 (-24 to 22.4) | -2 (-29.5 to 30.1) | 0.8 (-26.2 to 30.6) |
|  | **YLLs** | 31.39 (23.83 to 44.3) | 29.68 (20.3 to 44.91) | 32.88 (23.08 to 47.43) | 18.23 (15.03 to 23.59) | 16 (12.55 to 21.07) | 20.43 (15.42 to 28.02) | -41.9 (-58.4 to -17.8) | -46.1 (-65.1 to -14.5) | -37.8 (-60.7 to -6.9) |
|  | **YLDs** | 14.09 (9.22 to 20.57) | 13.84 (8.99 to 20.36) | 14.3 (9.19 to 21.06) | 26.87 (17.66 to 39.23) | 26.65 (17.17 to 39.52) | 27.11 (17.65 to 39.67) | 90.7 (75.5 to 107.6) | 92.6 (71.5 to 116) | 89.5 (70.1 to 111.9) |
| **Lorestan** | **Incidence** | 5.65 (4.54 to 6.97) | 5.52 (4.42 to 6.88) | 5.79 (4.63 to 7.12) | 10.93 (8.83 to 13.46) | 10.74 (8.74 to 13.24) | 11.1 (8.83 to 13.72) | 93.4 (84 to 102.9) | 94.7 (83.2 to 107.6) | 91.5 (81 to 105.1) |
|  | **Prevalence** | 195.12 (152.69 to 244.72) | 190.23 (148.75 to 239.22) | 199.64 (155.94 to 248.67) | 386.15 (304.24 to 478.45) | 378.18 (298.85 to 472.12) | 393.95 (307.31 to 490.74) | 97.9 (87.1 to 109) | 98.8 (85.5 to 113.6) | 97.3 (84.8 to 112.8) |
|  | **Deaths** | 0.97 (0.69 to 1.25) | 0.95 (0.58 to 1.34) | 0.99 (0.69 to 1.4) | 0.7 (0.56 to 0.87) | 0.57 (0.42 to 0.8) | 0.83 (0.61 to 1.11) | -28 (-46.8 to 4.8) | -40 (-61.8 to 7.8) | -15.8 (-45.2 to 26.4) |
|  | **DALYs** | 44.6 (35.85 to 55.25) | 43.81 (32.45 to 56.4) | 45.23 (34.74 to 57.85) | 47.83 (37.38 to 61.84) | 43.64 (33.29 to 57.47) | 52.11 (40.1 to 67.75) | 7.2 (-14.7 to 32.8) | -0.4 (-25.7 to 34.6) | 15.2 (-13.7 to 49.1) |
|  | **YLLs** | 30.14 (23.26 to 38.28) | 29.57 (19.75 to 41.21) | 30.59 (21.94 to 41.74) | 19.48 (15.86 to 24.14) | 15.53 (11.74 to 21.06) | 23.5 (17.62 to 30.83) | -35.4 (-51.7 to -8.7) | -47.5 (-66.1 to -12.3) | -23.2 (-49.3 to 17.7) |
|  | **YLDs** | 14.46 (9.26 to 21.35) | 14.24 (9.02 to 20.92) | 14.64 (9.4 to 21.85) | 28.35 (18.74 to 41.67) | 28.11 (18.7 to 40.81) | 28.61 (18.55 to 42.45) | 96.1 (80.2 to 114.6) | 97.4 (74.6 to 122.4) | 95.4 (75.6 to 117.1) |
| **Markazi** | **Incidence** | 5.77 (4.64 to 7.16) | 5.62 (4.48 to 7.03) | 5.92 (4.7 to 7.32) | 11.09 (8.93 to 13.63) | 10.88 (8.78 to 13.42) | 11.28 (9.02 to 13.87) | 92.2 (84 to 102.2) | 93.5 (81.6 to 106.3) | 90.5 (78.9 to 101.2) |
|  | **Prevalence** | 198.28 (155.35 to 248.4) | 193.18 (150.11 to 244.14) | 203.4 (158.19 to 254.72) | 390.19 (307.93 to 486.19) | 381.47 (300.4 to 476.93) | 398.61 (312.52 to 496.71) | 96.8 (86.4 to 108.3) | 97.5 (83 to 113.2) | 96 (82 to 107.9) |
|  | **Deaths** | 1.31 (0.9 to 1.7) | 1.34 (0.84 to 1.88) | 1.28 (0.82 to 1.81) | 0.75 (0.57 to 0.9) | 0.73 (0.52 to 0.95) | 0.76 (0.55 to 0.98) | -43.1 (-58.5 to -18.2) | -45.6 (-64.7 to -11.1) | -40.3 (-59.4 to -2.4) |
|  | **DALYs** | 52.02 (41.74 to 63.07) | 51.63 (38.99 to 66.51) | 52.48 (40.6 to 67.28) | 52.04 (40.97 to 65.81) | 49.84 (38.36 to 63.75) | 54.13 (41.42 to 69.1) | 0 (-20.5 to 24.5) | -3.5 (-29.2 to 26.9) | 3.1 (-23 to 33.6) |
|  | **YLLs** | 37.3 (28.65 to 47.07) | 37.15 (25.91 to 50.51) | 37.53 (27.01 to 51.11) | 23.4 (18.67 to 27.67) | 21.51 (14.79 to 27.51) | 25.18 (19.33 to 31.69) | -37.3 (-53.7 to -13.8) | -42.1 (-63.4 to -11) | -32.9 (-54.6 to 0.7) |
|  | **YLDs** | 14.72 (9.59 to 21.57) | 14.48 (9.48 to 21.25) | 14.95 (9.49 to 22.2) | 28.64 (18.68 to 42.16) | 28.33 (18.2 to 41.34) | 28.95 (18.64 to 43.09) | 94.6 (78.3 to 111) | 95.7 (74.1 to 120.2) | 93.7 (74.4 to 117.3) |
| **Mazandaran** | **Incidence** | 5.88 (4.72 to 7.24) | 5.74 (4.62 to 7.09) | 6.01 (4.81 to 7.41) | 11.54 (9.28 to 14.21) | 11.34 (9.16 to 13.95) | 11.71 (9.37 to 14.4) | 96.4 (88.1 to 105) | 97.8 (87.5 to 109.3) | 94.8 (84.6 to 105.9) |
|  | **Prevalence** | 202.45 (158.1 to 253.94) | 197.31 (154.49 to 247.88) | 207.48 (162.33 to 258.25) | 404.41 (317.68 to 502.16) | 395.79 (312.32 to 493.97) | 412.89 (323.99 to 509.71) | 99.8 (90.1 to 110.3) | 100.6 (88.3 to 114.3) | 99 (86.5 to 111.9) |
|  | **Deaths** | 1.12 (0.69 to 1.47) | 1.21 (0.63 to 1.71) | 1.02 (0.6 to 1.46) | 0.78 (0.55 to 0.93) | 0.76 (0.38 to 0.97) | 0.8 (0.57 to 1.02) | -30.3 (-48.3 to 3.9) | -36.7 (-57.2 to 1.3) | -22.1 (-48.8 to 27.6) |
|  | **DALYs** | 51.1 (37.1 to 64.53) | 53.69 (32.69 to 71.25) | 48.41 (35.19 to 62.13) | 55.24 (42.36 to 70.97) | 53.4 (38.21 to 69.87) | 57.04 (44.3 to 73.41) | 8.1 (-13.2 to 37.2) | -0.6 (-24.5 to 36.4) | 17.8 (-11.2 to 53.4) |
|  | **YLLs** | 36.14 (23.56 to 46.61) | 39.03 (19.19 to 54.58) | 33.16 (21.68 to 44.82) | 25.62 (18.02 to 30.6) | 24.21 (10.55 to 30.71) | 26.97 (19.84 to 33.97) | -29.1 (-46.2 to -1.2) | -38 (-56.9 to -5.8) | -18.7 (-46 to 23.2) |
|  | **YLDs** | 14.96 (9.7 to 21.99) | 14.66 (9.51 to 21.68) | 15.25 (9.72 to 22.45) | 29.62 (19.17 to 42.59) | 29.19 (18.98 to 42.58) | 30.06 (19.21 to 43.14) | 98 (82.5 to 114.1) | 99.1 (77.3 to 122.5) | 97.2 (77 to 119.2) |
| **North Khorasan** | **Incidence** | 5.82 (4.66 to 7.19) | 5.68 (4.54 to 7.03) | 5.96 (4.76 to 7.33) | 10.85 (8.76 to 13.37) | 10.65 (8.58 to 13.15) | 11.04 (8.82 to 13.55) | 86.6 (77.9 to 98.1) | 87.5 (75.7 to 102.8) | 85.2 (74.6 to 97) |
|  | **Prevalence** | 198.45 (155.71 to 247.96) | 193.64 (150.55 to 243.96) | 203.13 (158.19 to 254.19) | 382.57 (300.65 to 476.32) | 374.03 (292.59 to 469.64) | 391.19 (305.04 to 484.19) | 92.8 (82.5 to 105.7) | 93.2 (79.5 to 111) | 92.6 (80.2 to 106.2) |
|  | **Deaths** | 1.37 (0.84 to 1.83) | 1.52 (0.82 to 2.22) | 1.22 (0.75 to 1.77) | 0.8 (0.67 to 0.96) | 0.9 (0.71 to 1.17) | 0.72 (0.57 to 0.92) | -41.4 (-59.1 to -1.1) | -41 (-64.3 to 14.3) | -40.8 (-62 to 1.4) |
|  | **DALYs** | 54.75 (42.22 to 68.13) | 58.6 (41.12 to 78.46) | 51.05 (38.53 to 65.74) | 51.68 (41.59 to 64.73) | 52.49 (41.88 to 66.23) | 50.99 (39.84 to 64.09) | -5.6 (-26.4 to 24.6) | -10.4 (-35.7 to 27) | -0.1 (-26.8 to 30.5) |
|  | **YLLs** | 40.12 (28.85 to 51.66) | 44.16 (28.45 to 62.64) | 36.25 (25.9 to 50.84) | 23.79 (20.22 to 27.71) | 24.93 (20.38 to 30.8) | 22.76 (18.44 to 28.5) | -40.7 (-57 to -9.9) | -43.6 (-63.2 to -6) | -37.2 (-58.6 to -3.2) |
|  | **YLDs** | 14.63 (9.51 to 21.27) | 14.43 (9.14 to 20.94) | 14.8 (9.6 to 22.01) | 27.89 (18.65 to 40.79) | 27.56 (18.17 to 39.98) | 28.23 (18.6 to 41.16) | 90.7 (74.6 to 108.7) | 91 (68.9 to 115.4) | 90.8 (71.7 to 114.3) |
| **Qazvin** | **Incidence** | 5.77 (4.64 to 7.12) | 5.62 (4.48 to 6.93) | 5.92 (4.76 to 7.28) | 10.94 (8.82 to 13.51) | 10.76 (8.65 to 13.34) | 11.1 (8.84 to 13.63) | 89.6 (81.7 to 98.2) | 91.5 (80.6 to 103.5) | 87.6 (77.9 to 99.3) |
|  | **Prevalence** | 198.15 (155.24 to 247.05) | 193.03 (150.77 to 241.21) | 202.98 (159.33 to 253.86) | 386.36 (303.49 to 476.72) | 378.62 (297.35 to 471.59) | 393.61 (307.89 to 488.17) | 95 (85.6 to 105.2) | 96.2 (83.3 to 110.4) | 93.9 (81.8 to 107.5) |
|  | **Deaths** | 1.02 (0.68 to 1.35) | 1.06 (0.6 to 1.54) | 0.99 (0.62 to 1.39) | 0.68 (0.56 to 0.81) | 0.61 (0.47 to 0.79) | 0.75 (0.58 to 0.95) | -33.5 (-52.3 to 3.6) | -41.9 (-62.7 to 2.1) | -24.1 (-50.5 to 24.4) |
|  | **DALYs** | 44.15 (34.68 to 55.02) | 45.15 (32.48 to 59.25) | 43.14 (32.42 to 55.61) | 47.43 (37.16 to 61.19) | 45.26 (34.97 to 58.84) | 49.58 (38.47 to 64.32) | 7.4 (-14.6 to 34.5) | 0.2 (-24.8 to 33.1) | 14.9 (-12.9 to 47.6) |
|  | **YLLs** | 29.41 (21.3 to 37.63) | 30.65 (19.81 to 43.47) | 28.19 (19.7 to 38.17) | 19.09 (15.81 to 22.75) | 17.18 (13.31 to 21.15) | 20.97 (16.6 to 26.53) | -35.1 (-52.9 to -5) | -43.9 (-62.8 to -11.2) | -25.6 (-50.3 to 15.3) |
|  | **YLDs** | 14.73 (9.71 to 21.35) | 14.5 (9.35 to 21.38) | 14.95 (9.71 to 21.82) | 28.34 (18.55 to 41.53) | 28.07 (18.25 to 41.05) | 28.61 (18.85 to 41.71) | 92.4 (76.7 to 108.6) | 93.6 (71.8 to 118.2) | 91.4 (72.3 to 114.8) |
| **Qom** | **Incidence** | 5.73 (4.64 to 7.11) | 5.58 (4.45 to 6.91) | 5.88 (4.74 to 7.22) | 10.76 (8.62 to 13.35) | 10.58 (8.5 to 13.09) | 10.95 (8.68 to 13.47) | 87.7 (77.4 to 95.9) | 89.5 (78.1 to 100.7) | 86.3 (75.8 to 97.7) |
|  | **Prevalence** | 197.12 (155.62 to 245.63) | 191.95 (150.56 to 242.95) | 201.83 (159.47 to 251.35) | 381.6 (298.04 to 474.79) | 373.52 (292.5 to 470.32) | 389.44 (301.79 to 483.51) | 93.6 (81.8 to 102.6) | 94.6 (80.9 to 107.9) | 93 (79.6 to 106) |
|  | **Deaths** | 1.38 (0.73 to 1.89) | 1.47 (0.71 to 2.21) | 1.27 (0.64 to 1.95) | 0.74 (0.54 to 0.9) | 0.78 (0.55 to 0.99) | 0.72 (0.49 to 0.96) | -46.7 (-62.7 to -6) | -47.4 (-67.6 to 2.5) | -43.5 (-64.9 to 9.6) |
|  | **DALYs** | 55.69 (36.43 to 71.7) | 57.78 (32.99 to 79.74) | 53.49 (36.07 to 72.04) | 49 (38.22 to 62.6) | 47.5 (36.92 to 61.51) | 50.59 (38.38 to 65.84) | -12 (-34.6 to 25.3) | -17.8 (-42.2 to 30.1) | -5.4 (-31.6 to 36.3) |
|  | **YLLs** | 41.16 (23.61 to 55.21) | 43.48 (19.47 to 62.59) | 38.76 (23 to 56.5) | 21.08 (15.79 to 25.66) | 19.83 (13.65 to 25.11) | 22.4 (16.01 to 29.53) | -48.8 (-63.8 to -12.7) | -54.4 (-71.4 to -13) | -42.2 (-63.2 to 5.6) |
|  | **YLDs** | 14.52 (9.54 to 21.42) | 14.3 (9.3 to 21.08) | 14.73 (9.47 to 21.78) | 27.93 (18.12 to 40.54) | 27.66 (17.95 to 40.27) | 28.19 (18.36 to 41.91) | 92.3 (75.6 to 107.9) | 93.4 (72.3 to 116.3) | 91.4 (71.4 to 113.7) |
| **Semnan** | **Incidence** | 6.05 (4.87 to 7.46) | 5.9 (4.69 to 7.29) | 6.18 (4.94 to 7.59) | 11.25 (9.1 to 13.88) | 11.06 (8.91 to 13.71) | 11.43 (9.15 to 14.06) | 86.1 (78.9 to 94.8) | 87.4 (76.8 to 99.3) | 84.9 (75.4 to 95.4) |
|  | **Prevalence** | 206.07 (160.79 to 256.75) | 201.17 (156.57 to 251.84) | 210.72 (165.26 to 262.71) | 395.71 (311.7 to 493.26) | 387.38 (303.74 to 486.84) | 403.61 (317.45 to 501.75) | 92 (84 to 102.4) | 92.6 (80.3 to 106.7) | 91.5 (80.3 to 104.6) |
|  | **Deaths** | 1.22 (0.85 to 1.58) | 1.17 (0.73 to 1.63) | 1.27 (0.81 to 1.76) | 0.73 (0.56 to 0.87) | 0.65 (0.41 to 0.82) | 0.82 (0.61 to 1.02) | -40.1 (-55.8 to -13.6) | -44.8 (-63 to -11.8) | -35.6 (-55.4 to 1.7) |
|  | **DALYs** | 50.62 (39.81 to 61.4) | 48.84 (36.58 to 62.82) | 52.39 (39.37 to 65.96) | 51.39 (39.56 to 65.93) | 48.31 (36.27 to 63.6) | 54.43 (41.63 to 69.67) | 1.5 (-18 to 26.8) | -1.1 (-24.7 to 28.2) | 3.9 (-20 to 34.3) |
|  | **YLLs** | 35.35 (26.44 to 44.24) | 33.77 (22.91 to 45.58) | 36.93 (25.8 to 48.76) | 22.39 (16.75 to 26.58) | 19.63 (10.74 to 24.53) | 25.11 (18.86 to 31.14) | -36.7 (-52.6 to -13) | -41.9 (-62.8 to -12.8) | -32 (-53.2 to 0.7) |
|  | **YLDs** | 15.27 (10 to 22.41) | 15.07 (9.77 to 22.06) | 15.46 (10.06 to 23.02) | 29 (19.07 to 42.61) | 28.68 (18.5 to 41.56) | 29.32 (19.26 to 43.21) | 90 (75.8 to 105.1) | 90.4 (70 to 112.8) | 89.7 (70.3 to 110.5) |
| **Sistan and Baluchistan** | **Incidence** | 5.63 (4.51 to 7) | 5.48 (4.37 to 6.79) | 5.79 (4.62 to 7.16) | 9.72 (7.82 to 12) | 9.53 (7.66 to 11.79) | 9.9 (7.91 to 12.15) | 72.7 (63.6 to 84) | 73.8 (63.2 to 86.9) | 71.1 (60.2 to 83.8) |
|  | **Prevalence** | 192.55 (150.72 to 241.77) | 187.51 (146.84 to 234.23) | 197.02 (155.13 to 247.15) | 349.15 (274.23 to 433.54) | 341.96 (268.68 to 428.55) | 356.28 (280.26 to 438.04) | 81.3 (69.7 to 94.5) | 82.4 (69.8 to 97.9) | 80.8 (66.8 to 96.4) |
|  | **Deaths** | 1.22 (0.74 to 1.62) | 1.16 (0.6 to 1.67) | 1.26 (0.69 to 1.82) | 0.91 (0.75 to 1.1) | 0.92 (0.7 to 1.22) | 0.91 (0.66 to 1.21) | -25.2 (-46.3 to 24.3) | -21.1 (-48.8 to 62) | -27.7 (-53.1 to 20.5) |
|  | **DALYs** | 49.56 (33.47 to 62.88) | 46.81 (31.33 to 63.99) | 51.74 (33.36 to 68.25) | 57.29 (46.93 to 69.99) | 56.1 (44.28 to 69.61) | 58.59 (45.91 to 73.49) | 15.6 (-8.8 to 64.4) | 19.8 (-13.2 to 77) | 13.2 (-15.7 to 65.2) |
|  | **YLLs** | 35.31 (19.5 to 46.32) | 32.78 (17.8 to 47.32) | 37.31 (19.65 to 51.38) | 31.92 (26.71 to 37.53) | 30.99 (24.29 to 39.19) | 32.94 (24.88 to 41.89) | -9.6 (-34.7 to 53.3) | -5.5 (-39.3 to 74.9) | -11.7 (-41.2 to 56.8) |
|  | **YLDs** | 14.25 (9.26 to 20.86) | 14.03 (9.25 to 20.62) | 14.42 (9.22 to 21.13) | 25.38 (16.49 to 36.88) | 25.11 (16.32 to 36.54) | 25.65 (16.63 to 37.74) | 78.1 (61.1 to 96.5) | 79 (59.2 to 102.4) | 77.8 (57.6 to 100.5) |
| **South Khorasan** | **Incidence** | 5.78 (4.67 to 7.14) | 5.64 (4.55 to 6.99) | 5.93 (4.75 to 7.29) | 10.8 (8.67 to 13.29) | 10.6 (8.59 to 13.01) | 10.97 (8.72 to 13.55) | 86.8 (78.7 to 96.1) | 88 (77.5 to 98.8) | 85 (74.5 to 97) |
|  | **Prevalence** | 198.34 (155.86 to 247.78) | 193.3 (152.1 to 245.1) | 203.18 (159.6 to 253.19) | 381.78 (298.98 to 475.9) | 373.79 (294.07 to 466.09) | 389.61 (304.44 to 486.36) | 92.5 (82.7 to 103) | 93.4 (81.1 to 106.2) | 91.8 (79.5 to 105.7) |
|  | **Deaths** | 1.17 (0.72 to 1.55) | 1.19 (0.65 to 1.73) | 1.15 (0.68 to 1.65) | 0.7 (0.58 to 0.84) | 0.68 (0.53 to 0.91) | 0.72 (0.53 to 0.91) | -40.4 (-57 to -2.2) | -42.7 (-63.7 to 12.3) | -37.7 (-60.4 to -0.3) |
|  | **DALYs** | 49.35 (37.64 to 61.17) | 49.01 (34.67 to 63.68) | 49.65 (36.75 to 64.44) | 48.05 (37.26 to 60.96) | 46.36 (36.09 to 59.61) | 49.79 (37.91 to 64.21) | -2.6 (-23.6 to 25.7) | -5.4 (-30.7 to 31.4) | 0.3 (-25.9 to 31.4) |
|  | **YLLs** | 34.73 (25.1 to 44.07) | 34.61 (21.78 to 48.4) | 34.84 (23.94 to 48.32) | 20.23 (17.23 to 23.94) | 18.84 (15.13 to 23.92) | 21.67 (17.34 to 26.89) | -41.8 (-56.2 to -14.7) | -45.6 (-64 to -8.3) | -37.8 (-59.7 to -5.7) |
|  | **YLDs** | 14.62 (9.45 to 21.53) | 14.4 (9.31 to 21.53) | 14.81 (9.52 to 21.8) | 27.82 (18.25 to 40.36) | 27.53 (17.78 to 40.01) | 28.13 (18.27 to 41.49) | 90.3 (75.5 to 105.5) | 91.2 (70.7 to 114.2) | 89.9 (69.8 to 112.3) |
| **Tehran** | **Incidence** | 6.21 (4.99 to 7.62) | 6.05 (4.86 to 7.43) | 6.34 (5.09 to 7.83) | 11.86 (9.56 to 14.51) | 11.67 (9.49 to 14.32) | 12.04 (9.66 to 14.76) | 91 (83.6 to 98.9) | 92.8 (83.6 to 103.4) | 89.8 (79.3 to 100.4) |
|  | **Prevalence** | 212.66 (167.27 to 264.56) | 207.11 (162.08 to 257.38) | 217.74 (171.2 to 272.65) | 414.44 (328.79 to 515.75) | 405.78 (320.59 to 506.66) | 423.06 (331.69 to 523.91) | 94.9 (85.9 to 104.5) | 95.9 (84.8 to 108.6) | 94.3 (82 to 107.5) |
|  | **Deaths** | 0.77 (0.44 to 1.01) | 0.84 (0.25 to 1.19) | 0.69 (0.44 to 1.01) | 0.46 (0.27 to 0.61) | 0.46 (0.16 to 0.58) | 0.45 (0.3 to 0.73) | -41.2 (-56.7 to -15.6) | -45.4 (-62.5 to -15.7) | -34.3 (-58.6 to 5.1) |
|  | **DALYs** | 47.82 (30.71 to 61.82) | 49.06 (26.34 to 66.68) | 46.41 (30.9 to 63.72) | 46.48 (33.91 to 60.52) | 44.37 (31.41 to 58.64) | 48.62 (35.27 to 64.7) | -2.8 (-25.5 to 30.7) | -9.6 (-33.5 to 38.1) | 4.8 (-23.9 to 39.6) |
|  | **YLLs** | 32.76 (16.89 to 44.72) | 34.37 (10.86 to 50.57) | 31.01 (17.18 to 46.72) | 17.34 (9.35 to 22.57) | 15.78 (5.69 to 20.68) | 18.94 (10.95 to 28.35) | -47.1 (-61.8 to -18.7) | -54.1 (-68.4 to -26.5) | -38.9 (-62.2 to -1.2) |
|  | **YLDs** | 15.05 (9.88 to 22.22) | 14.69 (9.55 to 21.6) | 15.4 (10.07 to 22.93) | 29.14 (19.22 to 42.65) | 28.58 (18.55 to 42.38) | 29.69 (19.57 to 44.29) | 93.6 (79.5 to 109.2) | 94.6 (75.1 to 115.6) | 92.8 (73 to 114.7) |
| **West Azarbayejan** | **Incidence** | 5.56 (4.48 to 6.86) | 5.42 (4.33 to 6.73) | 5.7 (4.55 to 7.05) | 10.05 (8.07 to 12.38) | 9.87 (7.93 to 12.13) | 10.22 (8.14 to 12.6) | 80.8 (73.2 to 89) | 81.9 (71.1 to 93.7) | 79.3 (69.3 to 89.8) |
|  | **Prevalence** | 191.91 (149.71 to 240.12) | 187.12 (146.2 to 236.2) | 196.46 (152.91 to 244.64) | 360.54 (282.79 to 448.63) | 353.26 (277.46 to 439.69) | 367.57 (286.33 to 457.4) | 87.9 (79 to 97.2) | 88.8 (76.7 to 103) | 87.1 (76 to 98.6) |
|  | **Deaths** | 1.2 (0.86 to 1.55) | 1.2 (0.76 to 1.73) | 1.19 (0.82 to 1.59) | 0.78 (0.66 to 0.95) | 0.77 (0.62 to 1.05) | 0.78 (0.61 to 0.98) | -35.4 (-52 to -6.6) | -36 (-57.2 to 7.3) | -34.3 (-56.1 to -2.5) |
|  | **DALYs** | 48.07 (39.4 to 59.08) | 48.03 (37.01 to 64.25) | 47.95 (37.75 to 60.08) | 49.06 (39.45 to 61.83) | 48.1 (37.96 to 61.9) | 50 (39.26 to 63.1) | 2 (-19.7 to 25) | 0.1 (-26 to 29.2) | 4.3 (-19.9 to 31.8) |
|  | **YLLs** | 33.83 (26.73 to 43.83) | 34.03 (23.69 to 48.04) | 33.49 (24.93 to 44.01) | 22.78 (19.27 to 27.21) | 22.08 (17.8 to 27.88) | 23.45 (18.73 to 29.15) | -32.7 (-49.7 to -10.4) | -35.1 (-57 to -3.1) | -30 (-51.5 to 1.7) |
|  | **YLDs** | 14.24 (9.32 to 21.09) | 14 (8.9 to 20.67) | 14.45 (9.36 to 21.14) | 26.28 (17.26 to 38) | 26.02 (16.75 to 38.2) | 26.55 (17.3 to 38.73) | 84.5 (69.1 to 100.1) | 85.8 (64.3 to 109.5) | 83.7 (63.9 to 105.8) |
| **Yazd** | **Incidence** | 5.92 (4.77 to 7.39) | 5.76 (4.6 to 7.19) | 6.05 (4.87 to 7.55) | 11.43 (9.19 to 14.11) | 11.23 (9.01 to 13.89) | 11.61 (9.31 to 14.2) | 92.9 (83.9 to 102.2) | 94.9 (82.2 to 106.1) | 91.7 (81.7 to 105.1) |
|  | **Prevalence** | 202.49 (158.67 to 254.42) | 197.13 (153.95 to 249.22) | 207.35 (163.47 to 261.63) | 401.21 (315.9 to 498.44) | 392.37 (307.65 to 486.23) | 409.29 (321.57 to 507.47) | 98.1 (87.3 to 108.1) | 99 (84.1 to 112.6) | 97.4 (85.2 to 112.1) |
|  | **Deaths** | 1.15 (0.82 to 1.48) | 1.18 (0.7 to 1.67) | 1.1 (0.75 to 1.47) | 0.72 (0.57 to 0.87) | 0.7 (0.4 to 0.89) | 0.73 (0.56 to 0.93) | -37.8 (-54.2 to -12.1) | -40.9 (-60.7 to -2.3) | -33.9 (-56 to 2.2) |
|  | **DALYs** | 48.59 (38.16 to 60.09) | 49.02 (35.05 to 64.34) | 47.99 (36.74 to 59.73) | 53 (41.1 to 67.44) | 51.09 (37.72 to 66.64) | 54.67 (42.51 to 69.94) | 9.1 (-11.8 to 34.8) | 4.2 (-21.6 to 37) | 13.9 (-11.4 to 44.8) |
|  | **YLLs** | 33.58 (25.82 to 41.8) | 34.22 (21.8 to 47.49) | 32.77 (24.1 to 43.03) | 23.56 (18.19 to 28.04) | 21.96 (11.18 to 28.39) | 24.95 (18.85 to 31.5) | -29.8 (-47.9 to -3.3) | -35.8 (-57.5 to -0.7) | -23.9 (-47.2 to 13) |
|  | **YLDs** | 15.01 (9.81 to 22.17) | 14.8 (9.68 to 21.98) | 15.21 (9.8 to 22.47) | 29.44 (19.06 to 43.11) | 29.14 (18.8 to 42.51) | 29.72 (19.13 to 43.53) | 96.1 (80.1 to 112.8) | 96.9 (75.4 to 119.1) | 95.3 (76.7 to 118.3) |
| **Zanjan** | **Incidence** | 5.65 (4.54 to 6.96) | 5.49 (4.39 to 6.82) | 5.8 (4.64 to 7.14) | 10.73 (8.64 to 13.19) | 10.54 (8.49 to 12.96) | 10.9 (8.74 to 13.48) | 90.1 (81.1 to 99.6) | 91.9 (79.2 to 105.2) | 87.9 (79.1 to 98.2) |
|  | **Prevalence** | 194.83 (152.47 to 243.59) | 189.46 (147.52 to 238.28) | 200.01 (155.84 to 249.32) | 380.18 (299.45 to 472.81) | 372.43 (291.43 to 465.45) | 387.66 (304.64 to 480.32) | 95.1 (84.3 to 106) | 96.6 (81.8 to 112) | 93.8 (83 to 106.1) |
|  | **Deaths** | 0.8 (0.59 to 1.25) | 0.77 (0.48 to 1.49) | 0.82 (0.58 to 1.12) | 0.52 (0.42 to 0.72) | 0.47 (0.36 to 0.79) | 0.57 (0.44 to 0.83) | -35 (-52.1 to -4.8) | -38.4 (-58.2 to -1.6) | -30.5 (-53.2 to 10.1) |
|  | **DALYs** | 36.72 (29.29 to 50.74) | 35.17 (26.45 to 54.43) | 38.1 (29.49 to 50.12) | 41.87 (31.61 to 54.96) | 39.85 (29.73 to 52.96) | 43.9 (32.89 to 57.85) | 14 (-14.8 to 37) | 13.3 (-21.3 to 41.7) | 15.2 (-10.7 to 46.3) |
|  | **YLLs** | 22.28 (16.66 to 35.36) | 20.94 (14.23 to 40.87) | 23.46 (16.47 to 34.75) | 14.04 (11.4 to 19.16) | 12.26 (9.5 to 18.3) | 15.81 (12.08 to 22.52) | -37 (-54.3 to -13.3) | -41.5 (-61.3 to -12.1) | -32.6 (-53.5 to 2.5) |
|  | **YLDs** | 14.45 (9.44 to 21.26) | 14.22 (9.24 to 21.32) | 14.64 (9.5 to 21.41) | 27.83 (18.55 to 40.85) | 27.59 (18.15 to 40.38) | 28.09 (18.56 to 41.23) | 92.7 (76.7 to 109.7) | 94 (72.6 to 117.7) | 91.8 (72.4 to 114.9) |

*Data in parentheses are 95% Uncertainty Intervals (95% UIs).
