# Supplementary material for: National and sub-national burden and trend of type 1 diabetes in 31 provinces of Iran, 1990–2019
Source: Sci Rep. 2023 Mar 14;13:4210. doi: 10.1038/s41598-023-31096-8 (PMC10014831; doi:10.1038/s41598-023-31096-8)
Supplement: Supplementary file 8 — Supplementary Information 8. [file 41598_2023_31096_MOESM8_ESM.docx]

| **Location** | **Measure** | **Age-standardized rate in**  **1990** | **Age-standardized rate in**  **2005** | **Age-standardized rate in**  **2019** | **% change**  **(1990 - 2005)** | **% change**  **(2005 - 2019)** |
| --- | --- | --- | --- | --- | --- | --- |
| Iran | Incidence | 5.8 (4.7 to 7.2) | 7.1 (5.7 to 8.7) | 11 (8.9 to 13.5) | 21.6% | 55.7% |
| Alborz | Incidence | 6.1 (4.9 to 7.6) | 7.5 (6 to 9.2) | 11.5 (9.3 to 14) | 21.5% | 54.1% |
| Ardebil | Incidence | 5.6 (4.5 to 6.9) | 6.7 (5.4 to 8.4) | 10.7 (8.6 to 13.3) | 21.0% | 59.2% |
| East Azarbayejan | Incidence | 5.7 (4.6 to 7) | 6.9 (5.5 to 8.5) | 10.7 (8.6 to 13.2) | 21.1% | 55.7% |
| West Azarbayejan | Incidence | 5.6 (4.5 to 6.9) | 6.6 (5.4 to 8.2) | 10.1 (8.1 to 12.4) | 19.5% | 51.3% |
| Bushehr | Incidence | 5.8 (4.6 to 7.1) | 7.1 (5.7 to 8.8) | 10.8 (8.7 to 13.4) | 23.8% | 52.0% |
| Chahar Mahaal and Bakhtiari | Incidence | 5.7 (4.5 to 7) | 6.9 (5.6 to 8.6) | 10.7 (8.7 to 13.2) | 22.5% | 55.0% |
| Fars | Incidence | 5.1 (4 to 6.5) | 6.4 (5.1 to 8.1) | 10.2 (7.9 to 12.6) | 25.2% | 57.6% |
| Gilan | Incidence | 6 (4.8 to 7.4) | 7.4 (5.9 to 9.1) | 11.5 (9.2 to 14) | 23.6% | 55.4% |
| Golestan | Incidence | 5.8 (4.7 to 7.1) | 7 (5.6 to 8.7) | 10.8 (8.6 to 13.2) | 20.9% | 54.1% |
| Hamadan | Incidence | 5.7 (4.6 to 7) | 6.9 (5.6 to 8.6) | 10.9 (8.8 to 13.6) | 21.8% | 58.0% |
| Hormozgan | Incidence | 5.7 (4.6 to 7.1) | 6.9 (5.5 to 8.6) | 10.7 (8.6 to 13.2) | 19.9% | 55.5% |
| Ilam | Incidence | 5.7 (4.5 to 7) | 7.2 (5.8 to 8.9) | 11.4 (9.2 to 14.1) | 26.5% | 60.0% |
| Isfahan | Incidence | 5.9 (4.7 to 7.2) | 7.2 (5.8 to 8.9) | 11.2 (9 to 13.8) | 23.4% | 54.7% |
| Kerman | Incidence | 5.9 (4.7 to 7.3) | 7.1 (5.7 to 8.8) | 10.9 (8.8 to 13.3) | 20.3% | 53.4% |
| Kermanshah | Incidence | 5.8 (4.6 to 7.1) | 6.9 (5.5 to 8.6) | 10.8 (8.7 to 13.3) | 19.7% | 56.7% |
| North Khorasan | Incidence | 5.8 (4.7 to 7.2) | 6.9 (5.6 to 8.5) | 10.9 (8.8 to 13.4) | 19.0% | 56.7% |
| Khorasan-e-Razavi | Incidence | 5.8 (4.7 to 7.2) | 7 (5.6 to 8.7) | 10.8 (8.7 to 13.4) | 20.6% | 55.0% |
| South Khorasan | Incidence | 5.8 (4.7 to 7.1) | 7 (5.6 to 8.7) | 10.8 (8.7 to 13.3) | 21.1% | 54.3% |
| Khuzestan | Incidence | 5.7 (4.6 to 7) | 6.8 (5.5 to 8.4) | 10.5 (8.4 to 12.9) | 20.0% | 53.8% |
| Kohgiluyeh and Boyer-Ahmad | Incidence | 5.6 (4.5 to 6.9) | 6.9 (5.5 to 8.5) | 10.6 (8.5 to 13.2) | 23.3% | 54.4% |
| Kurdistan | Incidence | 5.5 (4.4 to 6.8) | 6.6 (5.3 to 8.2) | 10.3 (8.3 to 12.8) | 19.5% | 56.5% |
| Lorestan | Incidence | 5.7 (4.5 to 7) | 6.9 (5.5 to 8.6) | 10.9 (8.8 to 13.5) | 22.4% | 58.0% |
| Markazi | Incidence | 5.8 (4.6 to 7.2) | 7.1 (5.7 to 8.7) | 11.1 (8.9 to 13.6) | 22.3% | 57.1% |
| Mazandaran | Incidence | 5.9 (4.7 to 7.2) | 7.3 (5.9 to 9) | 11.5 (9.3 to 14.2) | 24.7% | 57.5% |
| Qazvin | Incidence | 5.8 (4.6 to 7.1) | 7 (5.6 to 8.7) | 10.9 (8.8 to 13.5) | 21.8% | 55.7% |
| Qom | Incidence | 5.7 (4.6 to 7.1) | 7 (5.6 to 8.7) | 10.8 (8.6 to 13.3) | 21.5% | 54.5% |
| Semnan | Incidence | 6 (4.9 to 7.5) | 7.3 (5.9 to 9.1) | 11.3 (9.1 to 13.9) | 21.0% | 53.8% |
| Sistan and Baluchistan | Incidence | 5.6 (4.5 to 7) | 6.4 (5.1 to 8) | 9.7 (7.8 to 12) | 14.3% | 51.0% |
| Tehran | Incidence | 6.2 (5 to 7.6) | 7.5 (6 to 9.2) | 11.9 (9.6 to 14.5) | 21.2% | 57.6% |
| Yazd | Incidence | 5.9 (4.8 to 7.4) | 7.2 (5.8 to 8.9) | 11.4 (9.2 to 14.1) | 21.5% | 58.8% |
| Zanjan | Incidence | 5.6 (4.5 to 7) | 6.8 (5.5 to 8.4) | 10.7 (8.6 to 13.2) | 21.3% | 56.8% |

| **Location** | **Measure** | **Age-standardized rate in**  **1990** | **Age-standardized rate in**  **2005** | **Age-standardized rate in**  **2019** | **% change**  **(1990 - 2005)** | **% change**  **(2005 - 2019)** |
| --- | --- | --- | --- | --- | --- | --- |
| Iran | Prevalence | 199.6 (157.2 to 250) | 247.9 (193.6 to 312.2) | 388.9 (306.1 to 482.1) | 24.2% | 56.8% |
| Alborz | Prevalence | 210 (165.9 to 263.2) | 260.3 (203.4 to 325.3) | 404.1 (320.6 to 500.8) | 24.0% | 55.3% |
| Ardebil | Prevalence | 192.3 (150.9 to 242.8) | 238.3 (184.8 to 298.8) | 379.1 (296.7 to 473) | 23.9% | 59.1% |
| East Azarbayejan | Prevalence | 196.1 (154.4 to 245.5) | 242.6 (188.3 to 305.5) | 380.2 (299 to 468.6) | 23.7% | 56.7% |
| West Azarbayejan | Prevalence | 191.9 (149.7 to 240.1) | 235.2 (184.8 to 294.5) | 360.5 (282.8 to 448.6) | 22.6% | 53.3% |
| Bushehr | Prevalence | 198.4 (156 to 247.3) | 249.6 (194.5 to 313) | 383.9 (299.5 to 477.4) | 25.8% | 53.8% |
| Chahar Mahaal and Bakhtiari | Prevalence | 196.1 (153.1 to 245) | 245 (191 to 309.8) | 382.1 (299.1 to 473.5) | 24.9% | 55.9% |
| Fars | Prevalence | 179.8 (138.7 to 228.4) | 231.6 (177.6 to 293.2) | 366.3 (280.4 to 459.5) | 28.8% | 58.1% |
| Gilan | Prevalence | 204.8 (160.8 to 256.6) | 257.5 (201 to 323.4) | 402.3 (313.7 to 499) | 25.7% | 56.3% |
| Golestan | Prevalence | 197.7 (155.5 to 246.8) | 244.8 (192.4 to 305.8) | 380 (295.8 to 473.6) | 23.8% | 55.2% |
| Hamadan | Prevalence | 195.9 (153 to 244.6) | 243.9 (191.3 to 308) | 385.9 (302.6 to 480.4) | 24.5% | 58.2% |
| Hormozgan | Prevalence | 195.9 (153.5 to 247.1) | 241.1 (187.7 to 302.8) | 378.2 (295.7 to 472.9) | 23.1% | 56.8% |
| Ilam | Prevalence | 195.5 (152.9 to 244.4) | 250.6 (196.2 to 316.5) | 401 (316 to 496) | 28.2% | 60.0% |
| Isfahan | Prevalence | 202.3 (158.3 to 252) | 253.4 (197.7 to 319.9) | 395 (309.8 to 494.5) | 25.3% | 55.9% |
| Kerman | Prevalence | 202 (157.4 to 254.2) | 248.7 (194.9 to 311.7) | 384.6 (300.2 to 474.7) | 23.1% | 54.7% |
| Kermanshah | Prevalence | 197.7 (155.9 to 247.5) | 242.3 (188.3 to 305.4) | 381.9 (300.1 to 474.1) | 22.5% | 57.6% |
| North Khorasan | Prevalence | 198.5 (155.7 to 248) | 243 (190.9 to 303.5) | 382.6 (300.6 to 476.3) | 22.5% | 57.4% |
| Khorasan-e-Razavi | Prevalence | 198.4 (156.6 to 249.4) | 245 (191.9 to 307.8) | 382.9 (299.8 to 476.1) | 23.4% | 56.3% |
| South Khorasan | Prevalence | 198.3 (155.9 to 247.8) | 245.6 (192.1 to 310.3) | 381.8 (299 to 475.9) | 23.8% | 55.4% |
| Khuzestan | Prevalence | 195.7 (152.9 to 243.4) | 240 (187.8 to 299.2) | 373 (294 to 463.6) | 22.6% | 55.4% |
| Kohgiluyeh and Boyer-Ahmad | Prevalence | 192.8 (150.6 to 240.8) | 242.3 (188.7 to 303.8) | 377.1 (293.7 to 467.5) | 25.7% | 55.6% |
| Kurdistan | Prevalence | 191.2 (149.6 to 238.7) | 233.8 (181.9 to 291.4) | 368.4 (288.8 to 458.8) | 22.3% | 57.5% |
| Lorestan | Prevalence | 195.1 (152.7 to 244.7) | 243.7 (189.7 to 306.9) | 386.1 (304.2 to 478.5) | 24.9% | 58.5% |
| Markazi | Prevalence | 198.3 (155.4 to 248.4) | 247.3 (194.6 to 310.1) | 390.2 (307.9 to 486.2) | 24.7% | 57.8% |
| Mazandaran | Prevalence | 202.5 (158.1 to 253.9) | 256.2 (200.2 to 317.3) | 404.4 (317.7 to 502.2) | 26.5% | 57.9% |
| Qazvin | Prevalence | 198.2 (155.2 to 247.1) | 246.6 (192.5 to 310.9) | 386.4 (303.5 to 476.7) | 24.5% | 56.7% |
| Qom | Prevalence | 197.1 (155.6 to 245.6) | 244.8 (191.7 to 309.9) | 381.6 (298 to 474.8) | 24.2% | 55.9% |
| Semnan | Prevalence | 206.1 (160.8 to 256.7) | 254.9 (200.2 to 322) | 395.7 (311.7 to 493.3) | 23.7% | 55.2% |
| Sistan and Baluchistan | Prevalence | 192.6 (150.7 to 241.8) | 228.2 (178.1 to 287.1) | 349.2 (274.2 to 433.5) | 18.5% | 53.0% |
| Tehran | Prevalence | 212.7 (167.3 to 264.6) | 262.3 (205.6 to 328.4) | 414.4 (328.8 to 515.8) | 23.3% | 58.0% |
| Yazd | Prevalence | 202.5 (158.7 to 254.4) | 251.6 (197.6 to 314.4) | 401.2 (315.9 to 498.4) | 24.3% | 59.5% |
| Zanjan | Prevalence | 194.8 (152.5 to 243.6) | 240.9 (189.6 to 301.8) | 380.2 (299.5 to 472.8) | 23.7% | 57.8% |

| **Location** | **Measure** | **Age-standardized rate in**  **1990** | **Age-standardized rate in**  **2005** | **Age-standardized rate in**  **2019** | **% change**  **(1990 - 2005)** | **% change**  **(2005 - 2019)** |
| --- | --- | --- | --- | --- | --- | --- |
| Iran | Deaths | 1.1 (0.8 to 1.3) | 1 (0.8 to 1.1) | 0.7 (0.6 to 0.8) | -13.5% | -25.5% |
| Alborz | Deaths | 0.9 (0.6 to 1.2) | 0.8 (0.6 to 0.9) | 0.6 (0.5 to 0.8) | -17.2% | -17.7% |
| Ardebil | Deaths | 1.2 (0.7 to 1.6) | 0.9 (0.7 to 1.1) | 0.8 (0.6 to 0.9) | -22.3% | -18.4% |
| East Azarbayejan | Deaths | 1.1 (0.8 to 1.4) | 1 (0.9 to 1.2) | 0.7 (0.6 to 1) | -10.4% | -28.9% |
| West Azarbayejan | Deaths | 1.2 (0.9 to 1.5) | 1 (0.9 to 1.2) | 0.8 (0.7 to 1) | -15.8% | -23.3% |
| Bushehr | Deaths | 1.1 (0.7 to 1.5) | 1.1 (0.8 to 1.3) | 0.8 (0.6 to 0.9) | -2.1% | -29.2% |
| Chahar Mahaal and Bakhtiari | Deaths | 0.9 (0.6 to 1.2) | 0.7 (0.6 to 0.9) | 0.6 (0.5 to 0.7) | -15.1% | -22.4% |
| Fars | Deaths | 1.3 (0.8 to 1.6) | 1.2 (0.8 to 1.4) | 0.9 (0.7 to 1) | -8.5% | -26.4% |
| Gilan | Deaths | 1 (0.7 to 1.3) | 0.9 (0.8 to 1.1) | 0.7 (0.6 to 0.9) | -8.4% | -21.5% |
| Golestan | Deaths | 1.4 (0.9 to 1.8) | 1.3 (0.9 to 1.5) | 1.1 (0.8 to 1.2) | -4.1% | -19.6% |
| Hamadan | Deaths | 1 (0.8 to 1.4) | 0.8 (0.7 to 1.1) | 0.7 (0.6 to 0.9) | -20.0% | -15.9% |
| Hormozgan | Deaths | 1.3 (0.8 to 1.8) | 1.2 (0.8 to 1.4) | 0.8 (0.6 to 1) | -9.4% | -32.4% |
| Ilam | Deaths | 1.1 (0.7 to 1.4) | 1.1 (0.8 to 1.3) | 0.8 (0.7 to 1) | -2.7% | -20.6% |
| Isfahan | Deaths | 1.2 (0.7 to 1.7) | 1.1 (0.7 to 1.3) | 0.8 (0.5 to 1) | -10.3% | -23.4% |
| Kerman | Deaths | 1.5 (0.9 to 1.9) | 1.4 (0.9 to 1.6) | 1 (0.7 to 1.1) | -7.7% | -28.7% |
| Kermanshah | Deaths | 1.4 (0.9 to 1.8) | 1.3 (1 to 1.5) | 0.9 (0.7 to 1) | -9.3% | -31.8% |
| North Khorasan | Deaths | 1.4 (0.8 to 1.8) | 1.1 (0.8 to 1.2) | 0.8 (0.7 to 1) | -22.7% | -24.1% |
| Khorasan-e-Razavi | Deaths | 1.4 (0.9 to 1.9) | 1.2 (0.9 to 1.4) | 0.9 (0.7 to 1) | -15.0% | -27.5% |
| South Khorasan | Deaths | 1.2 (0.7 to 1.5) | 0.9 (0.7 to 1.1) | 0.7 (0.6 to 0.8) | -22.3% | -23.3% |
| Khuzestan | Deaths | 1.6 (0.8 to 2.2) | 1.5 (0.8 to 1.8) | 1.2 (0.6 to 1.5) | -7.2% | -22.2% |
| Kohgiluyeh and Boyer-Ahmad | Deaths | 1 (0.7 to 1.3) | 0.8 (0.6 to 1) | 0.6 (0.5 to 0.8) | -22.8% | -20.5% |
| Kurdistan | Deaths | 1 (0.8 to 1.4) | 0.9 (0.8 to 1.1) | 0.6 (0.5 to 0.8) | -16.9% | -31.8% |
| Lorestan | Deaths | 1 (0.7 to 1.2) | 0.8 (0.7 to 1) | 0.7 (0.6 to 0.9) | -14.9% | -15.5% |
| Markazi | Deaths | 1.3 (0.9 to 1.7) | 1.1 (0.9 to 1.3) | 0.7 (0.6 to 0.9) | -13.7% | -34.0% |
| Mazandaran | Deaths | 1.1 (0.7 to 1.5) | 0.9 (0.6 to 1.1) | 0.8 (0.6 to 0.9) | -16.9% | -16.1% |
| Qazvin | Deaths | 1 (0.7 to 1.4) | 1 (0.7 to 1.1) | 0.7 (0.6 to 0.8) | -6.5% | -28.8% |
| Qom | Deaths | 1.4 (0.7 to 1.9) | 1.3 (0.9 to 1.5) | 0.7 (0.5 to 0.9) | -5.5% | -43.6% |
| Semnan | Deaths | 1.2 (0.9 to 1.6) | 1.1 (0.8 to 1.3) | 0.7 (0.6 to 0.9) | -10.6% | -33.0% |
| Sistan and Baluchistan | Deaths | 1.2 (0.7 to 1.6) | 1.1 (0.8 to 1.2) | 0.9 (0.8 to 1.1) | -13.9% | -13.2% |
| Tehran | Deaths | 0.8 (0.4 to 1) | 0.6 (0.3 to 0.8) | 0.5 (0.3 to 0.6) | -18.8% | -27.6% |
| Yazd | Deaths | 1.2 (0.8 to 1.5) | 1 (0.8 to 1.2) | 0.7 (0.6 to 0.9) | -9.2% | -31.6% |
| Zanjan | Deaths | 0.8 (0.6 to 1.2) | 0.8 (0.7 to 1.1) | 0.5 (0.4 to 0.7) | -5.6% | -31.1% |

| **Location** | **Measure** | **Age-standardized rate in**  **1990** | **Age-standardized rate in**  **2005** | **Age-standardized rate in**  **2019** | **% change**  **(1990 - 2005)** | **% change**  **(2005 - 2019)** |
| --- | --- | --- | --- | --- | --- | --- |
| Iran | DALYs | 49.4 (40.1 to 58.1) | 49 (40.7 to 58.4) | 51.7 (40.9 to 65.1) | -0.7% | 5.4% |
| Alborz | DALYs | 45.8 (33.8 to 57.1) | 44.3 (35.9 to 55) | 49.7 (38.4 to 63.2) | -3.3% | 12.2% |
| Ardebil | DALYs | 53.7 (38.9 to 67.9) | 46.1 (37.7 to 55.9) | 50.1 (39.5 to 63.1) | -14.2% | 8.7% |
| East Azarbayejan | DALYs | 48 (38.5 to 60.3) | 49.3 (42 to 58.6) | 50.2 (40 to 63.4) | 2.7% | 1.8% |
| West Azarbayejan | DALYs | 48.1 (39.4 to 59.1) | 47 (40 to 56) | 49.1 (39.4 to 61.8) | -2.2% | 4.3% |
| Bushehr | DALYs | 46.9 (36.1 to 58.5) | 50.5 (41.3 to 60.8) | 51.1 (40.4 to 65.1) | 7.7% | 1.2% |
| Chahar Mahaal and Bakhtiari | DALYs | 39.9 (32 to 50.1) | 39.1 (31.9 to 48.8) | 44.4 (34.3 to 58.3) | -2.1% | 13.6% |
| Fars | DALYs | 48 (37.5 to 58.8) | 51.4 (42 to 60.9) | 53.8 (42.6 to 66.9) | 7.1% | 4.7% |
| Gilan | DALYs | 43 (34.3 to 55.7) | 45 (37.2 to 55.1) | 50.6 (39.5 to 65.5) | 4.7% | 12.4% |
| Golestan | DALYs | 54 (41.7 to 66.6) | 56 (45.9 to 66.7) | 60.8 (47.2 to 75.1) | 3.8% | 8.4% |
| Hamadan | DALYs | 44.5 (35.9 to 57.3) | 42.7 (35.5 to 52.8) | 48.7 (38.3 to 61.8) | -4.0% | 14.0% |
| Hormozgan | DALYs | 53.2 (37.7 to 67.3) | 51.6 (41.3 to 61.5) | 52.5 (41.2 to 66.1) | -3.0% | 1.7% |
| Ilam | DALYs | 44.6 (35 to 55.2) | 47.5 (38.9 to 57.1) | 53.6 (41.7 to 67.8) | 6.4% | 12.8% |
| Isfahan | DALYs | 48.2 (35.4 to 60.1) | 50.9 (39.1 to 61.5) | 54.7 (41.2 to 69.8) | 5.6% | 7.6% |
| Kerman | DALYs | 54.3 (41.8 to 67.7) | 55.8 (45.1 to 66.3) | 56 (43.9 to 70.9) | 2.7% | 0.4% |
| Kermanshah | DALYs | 53.2 (41 to 65.7) | 54.1 (45.3 to 64) | 54.2 (43.5 to 67) | 1.8% | 0.1% |
| North Khorasan | DALYs | 54.7 (42.2 to 68.1) | 48.4 (41.5 to 58.2) | 51.7 (41.6 to 64.7) | -11.6% | 6.8% |
| Khorasan-e-Razavi | DALYs | 54.5 (42.1 to 67.7) | 53.6 (45.6 to 63.6) | 54.1 (43.4 to 67.4) | -1.6% | 0.9% |
| South Khorasan | DALYs | 49.4 (37.6 to 61.2) | 44.3 (36.8 to 54) | 48.1 (37.3 to 61) | -10.2% | 8.4% |
| Khuzestan | DALYs | 64.8 (42.2 to 82.2) | 65.5 (44.1 to 78.9) | 66.2 (45.5 to 83) | 1.1% | 1.1% |
| Kohgiluyeh and Boyer-Ahmad | DALYs | 43.4 (34.7 to 53.6) | 41.6 (34 to 51.4) | 46.6 (36.2 to 59.9) | -4.3% | 12.1% |
| Kurdistan | DALYs | 45.5 (36.1 to 59.4) | 42.7 (35.4 to 52.7) | 45.1 (34.7 to 57.9) | -6.2% | 5.7% |
| Lorestan | DALYs | 44.6 (35.9 to 55.3) | 41.8 (34.8 to 50.6) | 47.8 (37.4 to 61.8) | -6.2% | 14.4% |
| Markazi | DALYs | 52 (41.7 to 63.1) | 52.6 (44 to 62.5) | 52 (41 to 65.8) | 1.0% | -1.0% |
| Mazandaran | DALYs | 51.1 (37.1 to 64.5) | 49.9 (38.8 to 61.5) | 55.2 (42.4 to 71) | -2.3% | 10.7% |
| Qazvin | DALYs | 44.1 (34.7 to 55) | 44.1 (36.2 to 53.5) | 47.4 (37.2 to 61.2) | 0% | 7.5% |
| Qom | DALYs | 55.7 (36.4 to 71.7) | 56.3 (44.1 to 67.4) | 49 (38.2 to 62.6) | 1.0% | -12.9% |
| Semnan | DALYs | 50.6 (39.8 to 61.4) | 50.5 (40.9 to 60.6) | 51.4 (39.6 to 65.9) | -0.3% | 1.8% |
| Sistan and Baluchistan | DALYs | 49.6 (33.5 to 62.9) | 49.6 (42.1 to 59.1) | 57.3 (46.9 to 70) | 0.2% | 15.4% |
| Tehran | DALYs | 47.8 (30.7 to 61.8) | 45.6 (30.6 to 57.7) | 46.5 (33.9 to 60.5) | -4.6% | 1.9% |
| Yazd | DALYs | 48.6 (38.2 to 60.1) | 50.8 (40.7 to 61) | 53 (41.1 to 67.4) | 4.5% | 4.3% |
| Zanjan | DALYs | 36.7 (29.3 to 50.7) | 38.3 (31.1 to 48.6) | 41.9 (31.6 to 55) | 4.3% | 9.4% |

| **Location** | **Measure** | **Age-standardized rate in**  **1990** | **Age-standardized rate in**  **2005** | **Age-standardized rate in**  **2019** | **% change**  **(1990 - 2005)** | **% change**  **(2005 - 2019)** |
| --- | --- | --- | --- | --- | --- | --- |
| Iran | YLLs | 34.7 (27.3 to 39.6) | 30.7 (25.5 to 33) | 23.5 (18.9 to 25.6) | -11.5% | -23.7% |
| Alborz | YLLs | 30.2 (20.3 to 39.1) | 24.8 (20 to 29) | 20 (16.1 to 23.6) | -17.8% | -19.5% |
| Ardebil | YLLs | 39.5 (26.5 to 52.2) | 28.3 (23.1 to 32) | 22.4 (18.8 to 26) | -28.2% | -21.1% |
| East Azarbayejan | YLLs | 33.4 (25.8 to 43.5) | 31.2 (27.7 to 35.5) | 22.3 (18.8 to 28) | -6.7% | -28.4% |
| West Azarbayejan | YLLs | 33.8 (26.7 to 43.8) | 29.6 (26.3 to 34.8) | 22.8 (19.3 to 27.2) | -12.6% | -23.0% |
| Bushehr | YLLs | 32.2 (22.2 to 41.1) | 31.9 (26.1 to 35.9) | 23 (17.8 to 27.1) | -0.8% | -27.9% |
| Chahar Mahaal and Bakhtiari | YLLs | 25.3 (19.6 to 34.6) | 20.8 (17.8 to 26) | 16.3 (13.3 to 20.3) | -18.0% | -21.5% |
| Fars | YLLs | 34.7 (25.9 to 44.2) | 34.3 (28.3 to 39.4) | 27.1 (21.8 to 32.2) | -1.3% | -20.9% |
| Gilan | YLLs | 27.8 (21.2 to 37) | 25.8 (22.7 to 30.9) | 21.1 (17.4 to 26.6) | -7.1% | -18.3% |
| Golestan | YLLs | 39.3 (28.6 to 50.2) | 37.8 (30.4 to 43.1) | 33 (25 to 38.7) | -3.8% | -12.8% |
| Hamadan | YLLs | 30.5 (23.6 to 41.7) | 25.1 (21.9 to 32.6) | 21.5 (17.9 to 26.4) | -17.8% | -14.6% |
| Hormozgan | YLLs | 38.7 (24.8 to 50.6) | 33.7 (25.8 to 38.3) | 24.8 (19.3 to 29.5) | -13.0% | -26.3% |
| Ilam | YLLs | 30.1 (22.1 to 38.9) | 28.7 (23.7 to 33.2) | 24.1 (20 to 28) | -4.4% | -16.2% |
| Isfahan | YLLs | 33.1 (22.6 to 43) | 31.9 (23.5 to 36.9) | 25.7 (17.3 to 30.9) | -3.6% | -19.5% |
| Kerman | YLLs | 39.3 (28.7 to 50.2) | 37.3 (29.7 to 42.6) | 27.9 (21.6 to 33.1) | -5.1% | -25.3% |
| Kermanshah | YLLs | 38.5 (28.3 to 49.3) | 36.1 (30.6 to 41.2) | 26.2 (22 to 30.9) | -6.3% | -27.3% |
| North Khorasan | YLLs | 40.1 (28.9 to 51.7) | 30.4 (26.8 to 34.4) | 23.8 (20.2 to 27.7) | -24.3% | -21.7% |
| Khorasan-e-Razavi | YLLs | 39.8 (29.5 to 50.5) | 35.3 (30.6 to 39.7) | 26.1 (21.7 to 30.4) | -11.1% | -26.1% |
| South Khorasan | YLLs | 34.7 (25.1 to 44.1) | 26.1 (22.4 to 29.6) | 20.2 (17.2 to 23.9) | -24.9% | -22.5% |
| Khuzestan | YLLs | 50.4 (29.1 to 66.1) | 47.7 (28.4 to 56.9) | 39.1 (21.7 to 48.4) | -5.3% | -18.0% |
| Kohgiluyeh and Boyer-Ahmad | YLLs | 29.2 (21.6 to 37.4) | 23.5 (19.7 to 29.4) | 19 (15.5 to 23.6) | -19.5% | -19.2% |
| Kurdistan | YLLs | 31.4 (23.8 to 44.3) | 25.3 (22.1 to 32.3) | 18.2 (15 to 23.6) | -19.3% | -28.1% |
| Lorestan | YLLs | 30.1 (23.3 to 38.3) | 23.7 (20.8 to 27.4) | 19.5 (15.9 to 24.1) | -21.5% | -17.7% |
| Markazi | YLLs | 37.3 (28.6 to 47.1) | 34.1 (28.8 to 38.4) | 23.4 (18.7 to 27.7) | -8.5% | -31.5% |
| Mazandaran | YLLs | 36.1 (23.6 to 46.6) | 30.8 (22.1 to 36.1) | 25.6 (18 to 30.6) | -14.7% | -16.9% |
| Qazvin | YLLs | 29.4 (21.3 to 37.6) | 25.7 (22.1 to 29.3) | 19.1 (15.8 to 22.8) | -12.6% | -25.7% |
| Qom | YLLs | 41.2 (23.6 to 55.2) | 38.1 (28.5 to 43.6) | 21.1 (15.8 to 25.7) | -7.5% | -44.7% |
| Semnan | YLLs | 35.3 (26.4 to 44.2) | 31.5 (24.3 to 35.9) | 22.4 (16.7 to 26.6) | -10.9% | -28.9% |
| Sistan and Baluchistan | YLLs | 35.3 (19.5 to 46.3) | 32.7 (28.2 to 38.3) | 31.9 (26.7 to 37.5) | -7.3% | -2.5% |
| Tehran | YLLs | 32.8 (16.9 to 44.7) | 27 (13.2 to 34.9) | 17.3 (9.3 to 22.6) | -17.6% | -35.8% |
| Yazd | YLLs | 33.6 (25.8 to 41.8) | 32 (24.9 to 36.7) | 23.6 (18.2 to 28) | -4.6% | -26.5% |
| Zanjan | YLLs | 22.3 (16.7 to 35.4) | 20.4 (17.6 to 28.7) | 14 (11.4 to 19.2) | -8.5% | -31.1% |

| **Location** | **Measure** | **Age-standardized rate in**  **1990** | **Age-standardized rate in**  **2005** | **Age-standardized rate in**  **2019** | **% change**  **(1990 - 2005)** | **% change**  **(2005 - 2019)** |
| --- | --- | --- | --- | --- | --- | --- |
| Iran | YLDs | 14.7 (9.5 to 21.5) | 18.3 (12.1 to 27.1) | 28.2 (18.6 to 41.4) | 24.7% | 54.4% |
| Alborz | YLDs | 15.6 (10.2 to 22.8) | 19.5 (12.8 to 28.5) | 29.7 (19.3 to 43.2) | 24.7% | 52.5% |
| Ardebil | YLDs | 14.2 (9.3 to 21) | 17.8 (11.6 to 26.4) | 27.7 (18.1 to 40.3) | 24.8% | 56.2% |
| East Azarbayejan | YLDs | 14.6 (9.5 to 21.4) | 18.1 (11.8 to 26.4) | 27.8 (18.2 to 40.3) | 24.1% | 53.9% |
| West Azarbayejan | YLDs | 14.2 (9.3 to 21.1) | 17.5 (11.4 to 25.6) | 26.3 (17.3 to 38) | 22.6% | 50.5% |
| Bushehr | YLDs | 14.7 (9.6 to 21.5) | 18.6 (12.2 to 27.4) | 28.1 (18.4 to 41.2) | 26.3% | 51.1% |
| Chahar Mahaal and Bakhtiari | YLDs | 14.6 (9.4 to 21.6) | 18.3 (12 to 27.2) | 28.1 (18.4 to 41.2) | 25.5% | 53.4% |
| Fars | YLDs | 13.3 (8.5 to 19.9) | 17.1 (11.2 to 25.6) | 26.7 (17 to 39.5) | 28.8% | 55.7% |
| Gilan | YLDs | 15.2 (10 to 22.4) | 19.2 (12.6 to 28.1) | 29.5 (19.4 to 43.5) | 26.0% | 53.7% |
| Golestan | YLDs | 14.7 (9.6 to 21.6) | 18.2 (11.9 to 26.8) | 27.8 (18.1 to 40.8) | 24.4% | 52.4% |
| Hamadan | YLDs | 14 (9 to 20.5) | 17.6 (11.5 to 25.8) | 27.2 (17.9 to 40.2) | 26.0% | 54.8% |
| Hormozgan | YLDs | 14.5 (9.4 to 21.4) | 17.9 (11.6 to 26.5) | 27.7 (18.3 to 40.3) | 23.7% | 54.1% |
| Ilam | YLDs | 14.6 (9.5 to 21.4) | 18.8 (12.3 to 27.7) | 29.5 (19.4 to 43.4) | 28.8% | 57.3% |
| Isfahan | YLDs | 15 (9.8 to 22.1) | 18.9 (12.5 to 27.7) | 29 (18.8 to 42.6) | 26.0% | 53.1% |
| Kerman | YLDs | 14.9 (9.8 to 22.1) | 18.4 (12.1 to 27.3) | 28.1 (18.1 to 41.5) | 23.2% | 52.4% |
| Kermanshah | YLDs | 14.7 (9.5 to 21.6) | 18 (11.9 to 26.7) | 28 (18.3 to 40.6) | 23.1% | 55.0% |
| North Khorasan | YLDs | 14.6 (9.5 to 21.3) | 18 (11.8 to 26.8) | 27.9 (18.7 to 40.8) | 23.3% | 54.7% |
| Khorasan-e-Razavi | YLDs | 14.7 (9.4 to 21.5) | 18.3 (12 to 26.8) | 28 (18.4 to 40.3) | 24.1% | 53.3% |
| South Khorasan | YLDs | 14.6 (9.5 to 21.5) | 18.2 (11.9 to 27.2) | 27.8 (18.3 to 40.4) | 24.6% | 52.7% |
| Khuzestan | YLDs | 14.4 (9.3 to 21.2) | 17.8 (11.7 to 26) | 27.1 (17.7 to 39.1) | 23.4% | 52.6% |
| Kohgiluyeh and Boyer-Ahmad | YLDs | 14.3 (9.2 to 21.2) | 18.1 (11.7 to 26.6) | 27.6 (18 to 40.3) | 26.8% | 52.8% |
| Kurdistan | YLDs | 14.1 (9.2 to 20.6) | 17.3 (11.4 to 25.6) | 26.9 (17.7 to 39.2) | 22.9% | 55.2% |
| Lorestan | YLDs | 14.5 (9.3 to 21.4) | 18.2 (11.8 to 26.8) | 28.4 (18.7 to 41.7) | 25.6% | 56.2% |
| Markazi | YLDs | 14.7 (9.6 to 21.6) | 18.4 (12.1 to 27.1) | 28.6 (18.7 to 42.2) | 25.1% | 55.5% |
| Mazandaran | YLDs | 15 (9.7 to 22) | 19.1 (12.6 to 27.8) | 29.6 (19.2 to 42.6) | 27.5% | 55.3% |
| Qazvin | YLDs | 14.7 (9.7 to 21.4) | 18.4 (12 to 26.9) | 28.3 (18.6 to 41.5) | 25.0% | 53.8% |
| Qom | YLDs | 14.5 (9.5 to 21.4) | 18.2 (11.9 to 26.7) | 27.9 (18.1 to 40.5) | 25.2% | 53.6% |
| Semnan | YLDs | 15.3 (10 to 22.4) | 19 (12.4 to 28.2) | 29 (19.1 to 42.6) | 24.5% | 52.6% |
| Sistan and Baluchistan | YLDs | 14.3 (9.3 to 20.9) | 16.9 (11.1 to 24.8) | 25.4 (16.5 to 36.9) | 18.7% | 50.0% |
| Tehran | YLDs | 15.1 (9.9 to 22.2) | 18.6 (12.4 to 27.5) | 29.1 (19.2 to 42.6) | 23.6% | 56.6% |
| Yazd | YLDs | 15 (9.8 to 22.2) | 18.8 (12.2 to 27.4) | 29.4 (19.1 to 43.1) | 24.9% | 57.0% |
| Zanjan | YLDs | 14.4 (9.4 to 21.3) | 17.9 (11.6 to 26.2) | 27.8 (18.5 to 40.8) | 24.0% | 55.4% |
