# Supplementary material for: National and sub-national burden and trend of type 1 diabetes in 31 provinces of Iran, 1990–2019
Source: Sci Rep. 2023 Mar 14;13:4210. doi: 10.1038/s41598-023-31096-8 (PMC10014831; doi:10.1038/s41598-023-31096-8)
Supplement: Supplementary file 9 — Supplementary Information 9. [file 41598_2023_31096_MOESM9_ESM.docx]

| Location | | Sex | New cases | | Expected new cases in 2019 | | % 1990 - 2019 new  cases change cause | | | % 1990 - 2019 new cases overall change |
| --- | --- | --- | --- | --- | --- | --- | --- | --- | --- | --- |
|  |  |  | 1990 | 2019 | Population  growth | Population  growth + Aging | Population growth | Age structure change | Incidence rate change |  |
| Iran (Islamic Republic of) | | Female | 1917 | 4205 | 2778 | 2224 | 44.9% | -28.9% | 103.3% | 119.4% |
|  |  | Male | 2103 | 4575 | 3010 | 2460 | 43.1% | -26.1% | 100.6% | 117.5% |
| Subnational | Alborz | Female | 52 | 150 | 103 | 81 | 99% | -42.7% | 135.2% | 191.5% |
|  |  | Male | 58 | 164 | 111 | 89 | 92% | -37.7% | 129% | 183.4% |
|  | Ardebil | Female | 38 | 62 | 42 | 32 | 10.9% | -27.4% | 77.6% | 61.1% |
|  |  | Male | 42 | 69 | 47 | 36 | 10.8% | -24.5% | 76.8% | 63.1% |
|  | Bushehr | Female | 24 | 63 | 40 | 33 | 64.7% | -26.3% | 123.5% | 161.9% |
|  |  | Male | 26 | 73 | 47 | 39 | 78% | -27.3% | 129.3% | 179.9% |
|  | Chahar Mahaal and Bakhtiari | Female | 25 | 50 | 33 | 26 | 36% | -28.2% | 97.1% | 104.8% |
|  |  | Male | 27 | 55 | 36 | 29 | 34.8% | -25.6% | 95.3% | 104.5% |
|  | East Azarbayejan | Female | 113 | 195 | 133 | 104 | 18.1% | -25.8% | 81% | 73.3% |
|  |  | Male | 122 | 215 | 145 | 116 | 18.2% | -23.5% | 80.9% | 75.6% |
|  | Fars | Female | 112 | 225 | 154 | 117 | 36.5% | -32.6% | 96.1% | 100% |
|  |  | Male | 122 | 247 | 166 | 127 | 35.4% | -31.5% | 98% | 101.8% |
|  | Gilan | Female | 78 | 128 | 87 | 67 | 11.7% | -26% | 78.4% | 64.1% |
|  |  | Male | 84 | 135 | 93 | 72 | 11.4% | -25% | 75% | 61.4% |
|  | Golestan | Female | 46 | 99 | 66 | 53 | 44.3% | -28.2% | 100.8% | 116.8% |
|  |  | Male | 49 | 107 | 70 | 58 | 43.5% | -24.7% | 100.1% | 118.9% |
|  | Hamadan | Female | 54 | 84 | 56 | 44 | 5.1% | -23.6% | 75.2% | 56.7% |
|  |  | Male | 61 | 92 | 62 | 49 | 1.8% | -21.6% | 71.3% | 51.5% |
|  | Hormozgan | Female | 31 | 99 | 63 | 53 | 105.1% | -32.3% | 151.3% | 224.1% |
|  |  | Male | 34 | 111 | 69 | 60 | 104.9% | -26.8% | 152% | 230% |
|  | Ilam | Female | 15 | 31 | 20 | 15 | 33.7% | -33.3% | 104.5% | 104.8% |
|  |  | Male | 17 | 34 | 22 | 17 | 30.4% | -27.7% | 101.8% | 104.6% |
|  | Isfahan | Female | 126 | 263 | 176 | 138 | 39.7% | -30.5% | 99% | 108.2% |
|  |  | Male | 142 | 284 | 192 | 152 | 34.6% | -28% | 92.7% | 99.3% |
|  | Kerman | Female | 65 | 174 | 114 | 94 | 76% | -30.3% | 123.7% | 169.4% |
|  |  | Male | 70 | 195 | 126 | 107 | 80.5% | -27.7% | 126.5% | 179.3% |
|  | Kermanshah | Female | 56 | 95 | 66 | 51 | 18.7% | -28.4% | 78.4% | 68.8% |
|  |  | Male | 63 | 104 | 72 | 57 | 14.1% | -24% | 75% | 65.1% |
|  | Khorasan-e-Razavi | Female | 161 | 347 | 228 | 186 | 41.7% | -26.2% | 100.8% | 116.3% |
|  |  | Male | 173 | 377 | 244 | 204 | 41.1% | -23% | 99.8% | 117.9% |
|  | Khuzestan | Female | 108 | 248 | 165 | 134 | 53.2% | -28.6% | 106% | 130.6% |
|  |  | Male | 119 | 273 | 180 | 149 | 51.3% | -26% | 104.1% | 129.3% |
|  | Kohgiluyeh and Boyer-Ahmad | Female | 16 | 37 | 25 | 20 | 51.9% | -31.6% | 109.8% | 130% |
|  |  | Male | 18 | 42 | 27 | 22 | 52% | -27.6% | 109.4% | 133.8% |
|  | Kurdistan | Female | 41 | 78 | 55 | 42 | 34.2% | -31.3% | 89.1% | 92% |
|  |  | Male | 44 | 86 | 59 | 47 | 33.5% | -27.7% | 87.4% | 93.2% |
|  | Lorestan | Female | 51 | 89 | 59 | 46 | 15.5% | -26% | 84% | 73.5% |
|  |  | Male | 57 | 98 | 64 | 51 | 13.2% | -22.3% | 82.3% | 73.2% |
|  | Markazi | Female | 40 | 71 | 48 | 37 | 18.5% | -26.9% | 84.2% | 75.9% |
|  |  | Male | 44 | 77 | 52 | 41 | 19.9% | -26.1% | 82.6% | 76.3% |
|  | Mazandaran | Female | 88 | 175 | 116 | 89 | 31.3% | -29.8% | 97.6% | 99.1% |
|  |  | Male | 94 | 186 | 124 | 97 | 31.6% | -28.9% | 94.8% | 97.6% |
|  | North Khorasan | Female | 21 | 45 | 29 | 24 | 37.6% | -22.4% | 100.6% | 115.8% |
|  |  | Male | 22 | 49 | 31 | 26 | 37.3% | -19.5% | 100.5% | 118.3% |
|  | Qazvin | Female | 32 | 65 | 44 | 34 | 40.4% | -31.8% | 97.6% | 106.1% |
|  |  | Male | 35 | 71 | 49 | 38 | 39.3% | -30% | 93.4% | 102.7% |
|  | Qom | Female | 25 | 70 | 46 | 37 | 85.8% | -36% | 132.9% | 182.7% |
|  |  | Male | 28 | 76 | 50 | 41 | 82.4% | -33.4% | 126.6% | 175.6% |
|  | Semnan | Female | 16 | 38 | 25 | 21 | 57.1% | -27.5% | 110% | 139.6% |
|  |  | Male | 18 | 42 | 28 | 23 | 52.9% | -25.7% | 104.2% | 131.4% |
|  | Sistan and Baluchistan | Female | 50 | 162 | 101 | 91 | 102% | -20.5% | 142.1% | 223.7% |
|  |  | Male | 53 | 177 | 107 | 100 | 101.9% | -13.1% | 144.9% | 233.6% |
|  | South Khorasan | Female | 22 | 44 | 28 | 23 | 24.1% | -19.4% | 91.7% | 96.4% |
|  |  | Male | 24 | 48 | 30 | 26 | 24.4% | -15.7% | 92.6% | 101.2% |
|  | Tehran | Female | 284 | 738 | 476 | 387 | 67.3% | -31.1% | 123.2% | 159.4% |
|  |  | Male | 317 | 783 | 509 | 420 | 60.5% | -28.1% | 114.6% | 147.1% |
|  | West Azarbayejan | Female | 76 | 163 | 112 | 90 | 46.8% | -28.4% | 95.4% | 113.8% |
|  |  | Male | 83 | 179 | 121 | 100 | 46.5% | -25.2% | 95% | 116.4% |
|  | Yazd | Female | 23 | 63 | 39 | 33 | 70.3% | -27.4% | 134.7% | 177.5% |
|  |  | Male | 26 | 70 | 43 | 36 | 68.1% | -26.9% | 128.8% | 170.1% |
|  | Zanjan | Female | 29 | 52 | 36 | 28 | 22.1% | -28.5% | 84.3% | 77.9% |
|  |  | Male | 32 | 57 | 39 | 31 | 21% | -26.1% | 81.6% | 76.4% |
